# Oxidative stress in the oral cavity is driven by individual-specific bacterial communities

**DOI:** 10.1038/s41522-018-0072-3

**Published:** 2018-11-27

**Authors:** Mária Džunková, Daniel Martinez-Martinez, Roman Gardlík, Michal Behuliak, Katarína Janšáková, Nuria Jiménez, Jorge F. Vázquez-Castellanos, Jose Manuel Martí, Giuseppe D’Auria, H. M. H. N. Bandara, Amparo Latorre, Peter Celec, Andrés Moya

**Affiliations:** 1 Department of Genomics and Health, Foundation for the Promotion of Health and Biomedical Research of Valencia Region (FISABIO-Public Health), Valencia, Spain; 2 CIBER in Epidemiology and Public Health (CIBEResp), Madrid, Spain; 3 Institute for Integrative Systems Biology (I2SysBio), The University of Valencia and The Spanish National Research Council (CSIC)-UVEG, Valencia, Spain; 4 Australian Centre for Ecogenomics, The University of Queensland, St Lucia, QLD Australia; 5 Institute of Molecular Biomedicine, Faculty of Medicine, Comenius University, Bratislava, Slovakia; 6 Institute of Physiology, Academy of Sciences of the Czech Republic, Praha, Czech Republic; 7 Institute of Physiology, Faculty of Medicine, Comenius University, Bratislava, Slovakia; 8 Sequencing and Bioinformatics Service of the Foundation for the Promotion of Health and Biomedical Research of Valencia Region (FISABIO-Public Health), Valencia, Spain; 9 School of Dentistry, The University of Queensland, Herston, QLD Australia

## Abstract

The term “bacterial dysbiosis” is being used quite extensively in metagenomic studies, however, the identification of harmful bacteria often fails due to large overlap between the bacterial species found in healthy volunteers and patients. We hypothesized that the pathogenic oral bacteria are individual-specific and they correlate with oxidative stress markers in saliva which reflect the inflammatory processes in the oral cavity. Temporally direct and lagged correlations between the markers and bacterial taxa were computed individually for 26 volunteers who provided saliva samples during one month (21.2 ± 2.7 samples/volunteer, 551 samples in total). The volunteers’ microbiomes differed significantly by their composition and also by their degree of microbiome temporal variability and oxidative stress markers fluctuation. The results showed that each of the marker-taxa pairs can have negative correlations in some volunteers while positive in others. *Streptococcus mutans*, which used to be associated with caries before the metagenomics era, had the most prominent correlations with the oxidative stress markers, however, these correlations were not confirmed in all volunteers. The importance of longitudinal samples collections in correlation studies was underlined by simulation of single sample collections in 1000 different combinations which produced contradictory results. In conclusion, the distinct intra-individual correlation patterns suggest that different bacterial consortia might be involved in the oxidative stress induction in each human subject. In the future, decreasing cost of DNA sequencing will allow to analyze multiple samples from each patient, which might help to explore potential diagnostic applications and understand pathogenesis of microbiome-associated oral diseases.

## Oral bacteria: The significance of difference

Repeatedly sampling oral bacterial species in 26 volunteers over one month revealed considerable personal differences in the bacteria associated with oral disease, and some significant variation in individuals at different times. The findings come from an international team of researchers led by Mária Džunková at the Foundation for the Promotion of Health and Biomedical Research of Valencia Region, Spain. They emphasize the importance of repeated sampling to understand the significance of changing and varying oral bacterial populations in both health and disease. The study used chemical markers of ‘oxidative stress’ due to inflammation as an indication of the presence of disease-causing bacteria. Different bacterial combinations seem associated with differing oxidative stress levels in different people. The increasing ease of identifying bacteria by DNA sequencing will improve understanding of individual variations, assisting diagnosis and treatment.

## Introduction

Recent metagenomic studies showed that the oral cavities affected by periodontitis, gingivitis, halitosis or dental caries, are colonized by a variety of microbial species, including those found in healthy oral microbiome.^1–3^ It suggests that the oral diseases are not caused by an overgrowth of a single pathogen as previously thought, such as *Streptococcus mutans* in dental caries,^4^ rather, they are caused by dysbiotic composition of the oral microbiome which has been revealed by comparison with healthy individuals.^5^ However, despite rigorous metagenomic sequencing efforts, there is no consensus about specific pathogens which cause these oral diseases.

When pathogens are being engulfed by human leukocytes, reactive oxygen species (ROS) are formed.^6–8^ Inflammation-related production of ROS might result in oxidative stress, which triggers structural and functional changes of proteins, lipids, and nucleic acids.^9^ The byproducts of these reactions are present in saliva and can be reliably quantified as marker of oxidative stress.^10^ The dynamic interactions between the immune system and the composition of the microbiome in an apparently healthy oral cavity is reflected in temporal variability of the oxidative stress marker levels in otherwise healthy subjects.^11–13^ The bacterial taxa likely differ in their ability to resist oxidative stress induced by immune cells. Even the presence of genes for antioxidant enzymes in bacterial genomes can be considered as an important virulence factor.^14,15^ However, responses to oxidative stress have been studied by a systematic approach only in *E. coli* so far^16^ and there are no detailed studies about consequences of the oxidative stress on oral microbiome.

The most commonly evaluated markers of oxidative stress reactions are associated with lipid peroxidation, protein oxidation, and the antioxidant status. As these markers are involved in different biochemical pathways in human tissues, they are likely to be independent and do not have to correlate necessarily with each other.^17,18^ For interpretation of the oxidative stress markers values, physiological role of ROS as signaling molecules should also be taken into account.^19^ Temporal variability of oxidative stress markers can be the consequence of unstable signaling that may or may not be related to the variability of the oral microbiome composition.

Although it is widely accepted that activation of immune cells during inflammation leads to increased production of ROS and oxidative stress, the interaction is far more complex. The degree of alteration of lipids and proteins by oxidative damage is dependent on the ability of tissues/cells to prevent this damage (antioxidative status or capacity).^20^ Neutrophils and macrophages are able to actively produce ROS to combat pathogens, while on the other hand, the microorganisms can also protect themselves with antioxidant enzymes.^14^ Some oral bacteria, such as Enterococci, are capable of endogenous production of ROS which makes interpretation of oxidative stress markers in saliva even more difficult.^21^

Several studies have reported that saliva from patients with periodontitis and/or dental caries exhibited elevated levels of oxidative stress markers and proteomic inflammatory markers.^22–26^ Thus, it suggests that focusing on the immune-, or host response related- markers is likely to result in precise identification of potential pathogenic bacterial species associated with oral diseases. It has been demonstrated that the levels of the oxidative stress markers rise temporally after consumption of meals containing ROS stimulating bacterial strains.^27^ The composition of the oral microbiome also shows intra-individual temporal variations.^28^ Herein, we hypothesized that the temporal variation of oxidative stress markers levels in saliva can correlate with the temporal variations of oral microbiota that are likely to stimulate ROS production. Hence, in the present study daily taxonomic composition of the oral microbiome and oxidative stress markers for 26 volunteers were evaluated for a period of one month. Subsequently, bacteria that might trigger inflammation in each volunteer were identified on individual-specific level.

## Results

### Volunteers’ microbiome had individual-specific composition

Twenty-six volunteers (13 women and 13 men) of age between 21 and 28 years participated in this study. On average 21 non-stimulated saliva samples (2 ml) were collected from each participant during the period of 30 days. DNA extracted from the saliva samples was amplified for the 16S ribosomal gene and sequenced. In total, 27,072,746 sequences from 551 samples collected from 26 volunteers have passed the quality filters and have been clustered into 18,146 operational taxonomic units (OTU). The final OTU names used in this study consisted from the assigned genus of the reference sequence and the number of the cluster.

The microbiome of all volunteers was dominated by the *Streptococcus*-OTU0 (highest similarity with *Streptococcus parasanguinis*) forming on average 37.5 ± 10.1% of the whole microbiome (Supplementary Figure S1) followed by taxon *Rothia*-OTU1 (20.9 ± 11.3%). The ratios of these two most dominant species differed significantly (*p* < 0.001, *t*-test) in 15% of pairwise comparisons among volunteers. For example, the proportion of *Rothia*-OTU1 could be as high as 35.3 ± 10.5% in volunteer F06 or as low as 5.4 ± 5.2% in volunteer M12. The overall microbiome composition of the volunteers differed significantly due to the less prevalent (average proportion 0.1–5.4%) individual-specific taxa (*p* < 0.001, “envfit” test, Supplementary Figure S2). Some volunteers (e.g., F18, F20, and M20) were characterized by high proportions of *Granulicatella*-OTU2 (8.7 ± 4.1%) and *Atopobium*-OTU3 (6.9 ± 3.7%), while other volunteers (e.g., M12, M21) had high proportions of *Gemella*-OTU6 (4.1 ± 3.1%). The volunteer F12 had the highest proportion of *Saccharibacteria*-OTU4 (8.9 ± 4.5%). Some volunteers did not possess any extreme proportions of the most detected prevalent OTUs (central part of the CCA plot in the Supplementary Figure S2). The indexes describing microbiome diversity, Shannon index (2.5 ± 0.4) and the evenness index (0.5 ± 0.1), also differed significantly (*p* < 0.001, *t*-test) in 11% of all pairwise volunteers combinations (Supplementary Figure S3).

Before commencing with the following analyses which may require larger computational memory, we used Procrust test to check whether reduction of the OTUs list would provide the same results as including all 18,146 detected OTUs (many of them may actually include OTUs corresponding to sequencing errors). The test showed that OTUs with proportions of above 0.1% (50 OTUs) result in the same ordination of samples as all detected OTUs (Supplementary Figure S4).

### Volunteers differed by degree of microbiome temporal variability

Changes of the OTUs relative abundances over time was assessed by Taylor’s equation *σ* = *V* × *μβ*, where *V* and *β* are the parameters of the model, and *σ* and *μ* are the dispersion (in standard deviation) and the mean of the measurements. The variability parameter *V* was the direct estimator of the amplitude of fluctuations and therefore of the general stability, while the *β* parameter, the scaling index, described the statistical behavior of the ecosystem, always between 0.5 (behaving as a Poisson process) and 1 (behaving as an exponential distribution).^29^

**Fig. 1:**
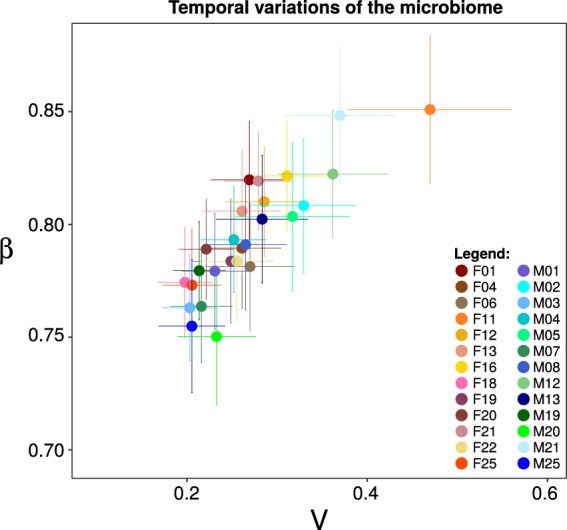
Temporal variability of the microbiome. Taylor’s Parameter space for the 26 volunteers of the study. *V* represents the *y*-intercept of the linear fit, and *β* to the slope of the line. Each individual has been placed in this plot according to its *V* and *β* value, where the error bars correspond to the standard error of the mean

All the values of the Taylor’s parameter *β* were between 0.5 and 1 which means that the most prevalent OTUs in all samples showed less relative variability over time than the less abundant OTUs (Fig. 1). The Taylor’s variability parameter *V* (average 0.27 ± 0.06) significantly varied in 26% of pairwise comparisons among volunteers (*p* < 0.001, *t*-test). The volunteer F11 had the least stable microbiome of our cohort (*V* = 0.47 ± 0.09), while the microbiome of F18 was the most stable (*V* = 0.20 ± 0.03).

**Fig. 2:**
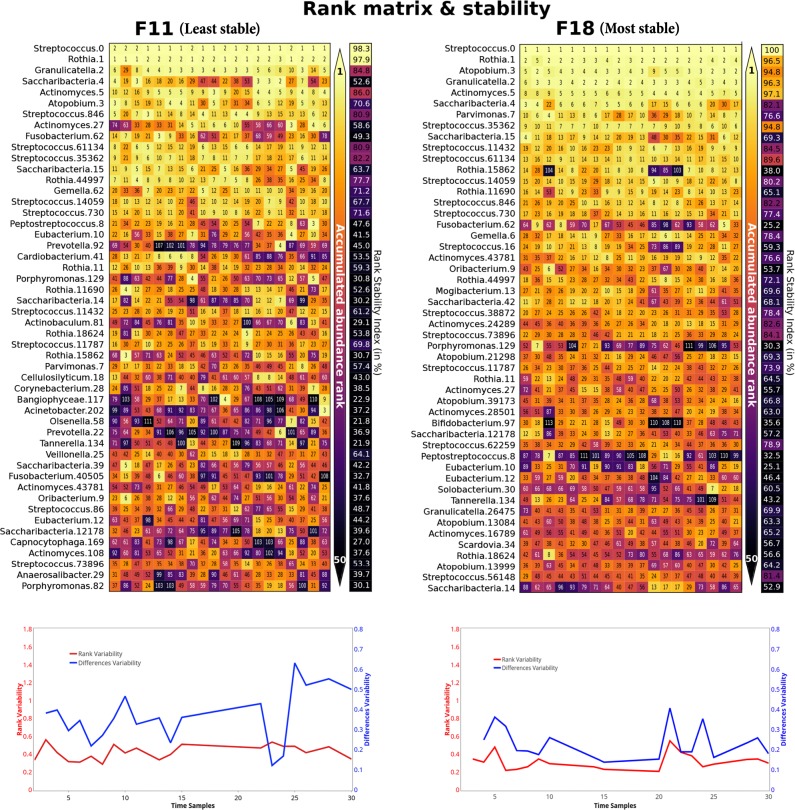
Rank matrix for the 50 most abundant OTUs for F11 and F18. Rank matrix corresponding to the most and less time-variable volunteers, F11 and F18 respectively. Both plots represent the 50 most abundant OTUs, and heat-map colors corresponds to the abundance of each OTU at each time, ranging from light-yellow for rank 1, to black, representing very low ranks. Alongside, the Rank Stability Index (RSI) is colored by the percentage of rank stability, following the same color-code as before. Below, both Rank and Difference Variability (DV) is plotted in red and blue colors for each time point

In addition, we calculated the difference variability (DV) and rank variability (RV) to detect time-points with highest variability on intra-individual level (Supplementary Figure S5). DV expresses an absolute difference between every OTU’s rank (proportion) at a specific time point compared to the previous time point. The DV was generally higher in the least stable volunteer F11 when compared to the most stable volunteer F18 (Fig. 2). This is expected because there are more rank differences in subjects with higher Taylor’s parameter V (F11) than in subjects with lower fluctuations (F18). In general, DV and RV are good estimators of community shifts on intra-individual level. For example, the volunteer F04 had a short community composition shift during the days 20–24, but later on the day 25 came back to the previous bacterial composition (Supplementary Figure S5).

The most prevalent OTUs *Rothia*-OTU1 and *Streptococcus*-OTU0 had the rank stability index (RSI) over 96% in both volunteers F11 and F18 (Fig. 2) which indicated that the most prevalent OTUs were very stable in the volunteer with the most stable microbiome as well as in the volunteer with the least stable microbiome. The microbiome temporal variability of the volunteers F11 and F18 differed due to the changing proportions of the less prevalent OTUs. The average RSI of all 50 OTUs in volunteers F11 (the least stable) and F18 (the most stable) were 52.3 ± 20.1% and 67.7 ± 19.2%, respectively. The volunteer F11 had 26 OTUs with RSI below 50%, while the volunteer F18 had only seven such highly unstable OTUs. When the OTUs were sorted according to their prevalence, the last OTU with an RSI above 70% in the least stable volunteer F11 was placed in the 16th position, while in the most stable volunteer F18 it was placed in the 49th position (Fig. 2). The OTUs found in lower proportions were in general less stable than the highly abundant OTUs (Supplementary Figure S5). However, the most prevalent OTUs were not always the most stable. For example, *Saccharibacteria*-OTU15 and *Rothia*-OTU15862 were in high proportion in F11 and F18, but it possessed a low RSI in the both volunteers (Fig. 2).

### Large inter-individual and intra-individual difference of the oxidative stress markers levels

Oxidative stress can be measured by estimating oxidative damage to lipids (lipid peroxidation) and proteins (protein oxidation), or by quantifying the capacity to resist oxidative damage (antioxidant capacity).^30^ The lipid peroxidation was quantified by measuring thiobarbituric acid reacting substances (TBARS, average 0.10 ± 0.18 μmol/l, Supplementary Figure S6). Advanced glycation end products (AGEs, 0.27 ± 0.19 g/l) and advanced oxidation protein products (AOPP, 37.6 ± 21.8 μmol/l) were herein quantified as carbonyl and oxidative stress markers respectively, to express oxidative protein damage. The capacity to resist oxidative damage was measured by total antioxidant capacity (TAC, 578.6 ± 149.4 μmol/l) and ferric reducing ability of saliva (FRAS, 396.7 ± 183.6 μmol/l). The values of the five markers possessed intra-individual temporal variations, and they also differed significantly (*p* < 0.001, *t*-test) among volunteers—in 44–73% of volunteers pairwise combinations (Supplementary Figure S6).

**Fig. 3:**
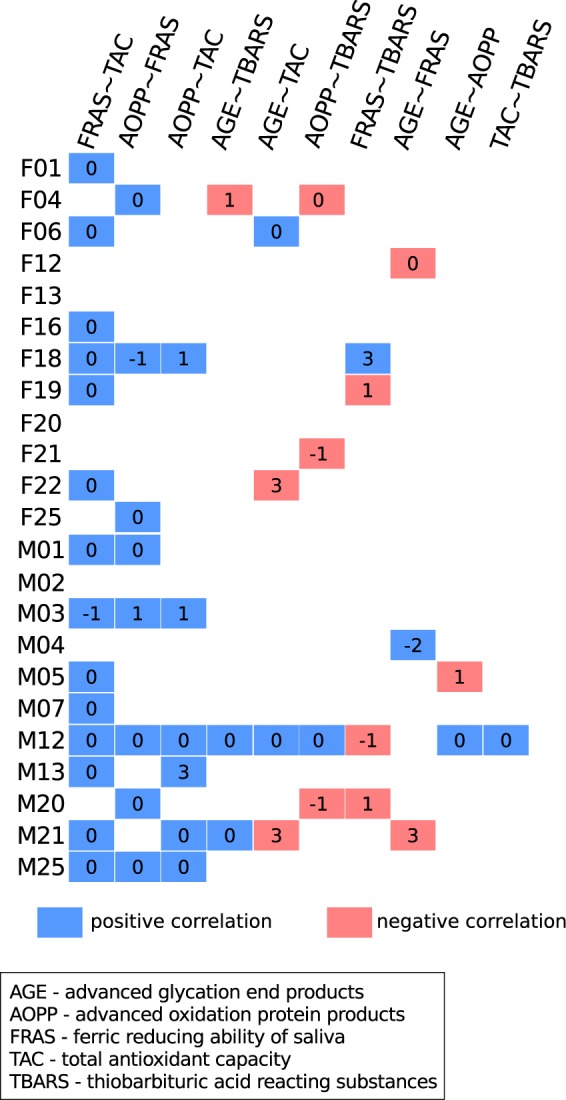
Correlations between the oxidative stress markers. The summary of the Pearson’s intra-individual correlations. The color of square indicates either a positive or a negative correlation (blue or red) of a pair of oxidative stress markers in a volunteer. The number in square indicates the days by which the correlation was lagged. Empty white squares indicate no significant correlation. The majority of volunteers possessed a positive correlation between FRAS and TAC. Several marker pairs had positive correlations in some volunteers while negative correlations in others

In addition, the five oxidative stress markers were tested for their pairwise correlations on intra-individual level. FRAS correlated positively with TAC in 14 out of the 26 volunteers, but the majority of the salivary markers pairwise combinations resulted in non-uniform correlation patterns on intra-individual level among the 26 volunteers (Fig. 3). Correlations lagged by 1–3 days accounted for 36.7% of all significant correlations detected, while the remaining 63.3% were temporally direct correlations.

### Correlations between bacterial taxa and oxidative stress markers are individual-specific

**Fig. 4:**
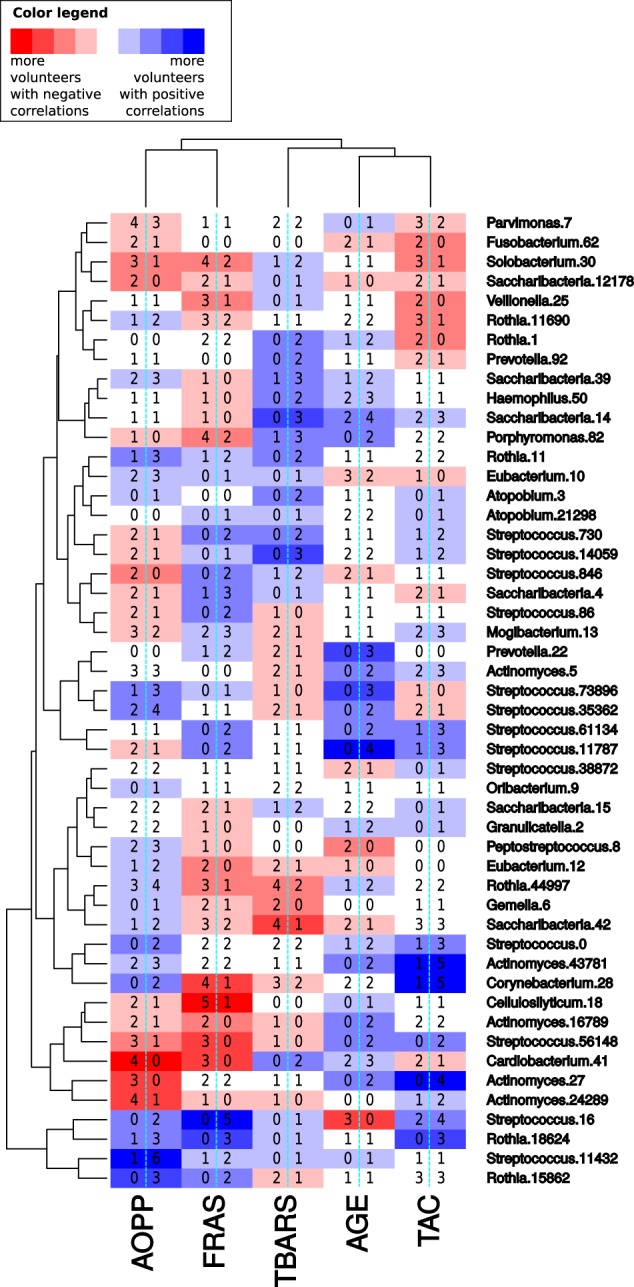
Marker-OTU correlations on intra-individual level. The summary of the Pearson’s correlation plot in the Supplementary Figure S7. The graduated color in tones from blue to white to red indicate whether the correlations found in the volunteers on intra-individual levels were mostly positive or negative. The number on the left in each rectangle indicates the number of volunteers with a negative correlation, while the number on the right in each rectangle indicates the number of volunteers with a positive correlation. The oxidative stress markers and the OTUs have been ordered according to their correlation tendency by hierarchical clustering using Euclidean distances. The OTUs with the strongest tendency of either positive or negative correlations with some of the oxidative stress markers were e.g. *Streptococcus*-OTU16 (*Streptococcus mutans*), *Actinomyces*-OTU27 and *Cardiobacterium*-OTU41

As much as 94% of the 250 marker-OTU pairs (5 markers × 50 most prevalent OTUs) had a significant correlation on intra-individual level in some of the 26 volunteers (Fig. 4, details in the Supplementary Figure S7). These correlations were either temporally direct or lagged by 1–3 days. The remaining 6% of the marker-OTU pairs did not show any significant correlation in any of the volunteers. The possible delay in the correlations between our variables was measured for all possible combinations, and the significant results were only found on a single lagged day as Fig. 3 shows.

The results of the Pearson’s correlations showed that a particular marker-OTU pair can have positive correlations in some volunteers, while negative correlations in others (Fig. 4, details in the Supplementary Figure S7). This result is consistent with Spearman correlation coefficients, a non-parametric correlation (Supplementary Figure S7). The volunteers with contradictory correlation results did not possess any extreme values of a given marker-OTU pair.

Importantly, there were some marker-OTU pairs that produced mostly non-significant correlations, but when they were found to be significant in some of the volunteers, they were exclusively either positive or negative in these volunteers (Fig. 4, details in the Supplementary Figure S7). The most prominent exclusive correlations with oxidative stress markers had *Streptococcus*-OTU16 (*S. mutans*), a well established causative agent of dental caries.^4^ *S. mutans* correlated positively with AOPP (in two volunteers), with FRAS (in five volunteers) and with TBARS (in one volunteer) and correlated negatively with AGE (in three volunteers). The proportion of *S. mutans* in the salivary microbiome was as low as 0.19 ± 0.47% in the studied cohort indicating that its significant correlations with the oxidative stress markers were independent of its proportion in the microbiome.

**Fig. 5:**
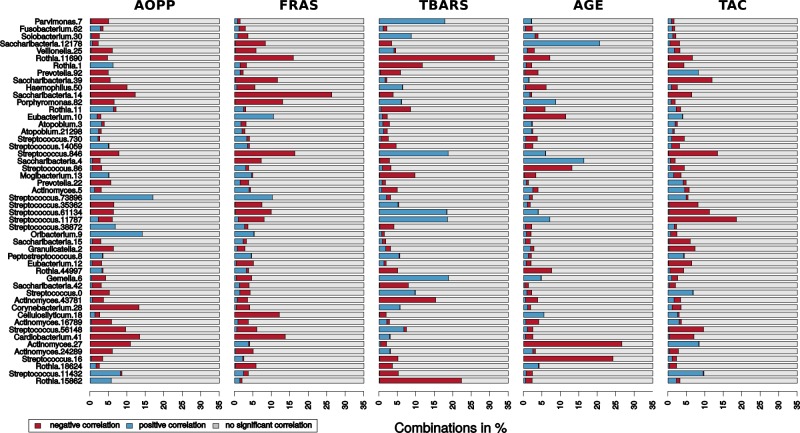
Marker-OTU correlations obtained by the simulation of collection of only one sample per volunteer. The bar-plots illustrate which portion (in %) of the 1000 combinations in the one-sample-one-volunteer approach simulation resulted in a positive correlation (blue), in a negative correlation (red) or no significant correlation (*p* > 0.05, grey). Both positive and negative correlations occurred in the majority of the marker-OTU pairs in low proportion. However, there were also some marker-OTU pairs with an explicitly positive or an explicitly negative correlation (no contradictory correlations were detected). The bacterial OTUs in this figure are arranged according to the Fig. 4, to make them easily comparable

Among other marker-OTU pairs with exclusively negative correlations found in a higher number of volunteers (3–6) were e.g., AOPP—*Cardiobacterium*-OTU41, AOPP—*Actinomyces*-OTU27, FRAS—*Streptococcus*-OTU56148 and FRAS—*Cardiobacterium*-OTU41. The marker-OTU pairs resulting in exclusive positive correlations were e.g. AOPP—*Rothia*-OTU15862, FRAS—*Rothia*-OTU18624, AGE—*Streptococcus*-OTU11787 and TAC—*Actinomyces*-OTU27 (Fig. 5).

### Correlations and interactions between bacterial OTUs are also individual-specific

Similar to the marker-OTU pairs, the significant intra-individual correlations found among the OTU–OTU pairs did not show any notable generalized pattern (Supplementary Figure S7). There were some particular OTU–OTU pairs resulting in consistent correlations among most of the volunteers, for example a negative correlation was found between *Streptococcus*-OTU0 and *Rothia*-OTU1 in 17 out of 26 volunteers, while in the remaining 9 volunteers no significant correlation was detected.

Also, we analysed the interactions of the 15 most abundant OTUs from each volunteer using the generalized Lotka-Volterra model, a system of equations that has been often used for the inference of bacterial interactions in complex ecosystems such as the human microbiome.^31^ These models allow us, when a temporal series is available, to make better predictions of bacterial interactions rather than the use of classic correlation coefficients that could be filled by false positives and false negatives due to compositional effects.^31^ The results from the Lotka-Volterra model are more accurate than using simple correlations in terms of microbial interactions,^32,33^ but it did not yield any generalized interaction pattern either (Supplementary Figure S8).

### Inconsistent results of the single sample collection simulation

In order to demonstrate the utility of the longitudinal sample collections in correlation studies, we simulated 1000 different single-samples combinations. Single samples from the 26 sample-sets were selected and combined to form a set of 26 samples in 1000 repetitions. Each of the 1000 simulated one-sample-one-volunteer sets was tested for correlations of oxidative stress markers with OTUs. The majority (82.4%) of the 250 marker—OTU pairs (5 markers x 50 OTUs) produced contradictory results. For example, a negative correlation was found in 22.2% of these single sample combinations for the TBARS—*Rothia*-OTU15862 pair, but still 0.1% of combinations for this marker—OTU pair resulted in a positive correlation, while 77.7% of the combinations did not yield any significant correlation (Fig. 5).

The results of the correlation analyses performed on this simulated one-sample-one-volunteer sets (Fig. 5) were not consistent with the results obtained on the intra-individual level (Fig. 4). For example, AGE—*Actinomyces*-OTU27 pair exhibited exclusively negative correlations in 26.6% of the one-sample-one-volunteer combinations, however, it showed positive correlations in two volunteers (F04, M01) on intra-individual level. Furthermore, the exclusive positive correlation of *S. mutans* (*Streptococcus*-OTU16) with FRAS observed in five volunteers on intra-individual level, was not confirmed by this one-sample-one-volunteer approach (only 2.2% of positive correlations and 0.3% of negative correlations in the 1000 combinations).

In contrast, in some cases the correlation results obtained by the two approaches were quite consistent; e.g., negative correlations between AGE—*S. mutans*, AOPP—*Actinomyces*-OTU27, AOPP—*Cardiobacterium*-OTU41, and FRAS—*Cardiobacterium*-OTU41.

## Discussion

*Streptococcus mutans* used to be associated with caries before the metagenomics era, while recent metagenomics studies have assumed that the oral diseases are of polymicrobial origin.^5,34–36^ Nevertheless, metagenomic studies based on comparing oral microbiome composition of infected and healthy subjects often lead to inconsistent results.^37–40^

We aimed to identify bacteria with pathogenic potential on individual-specific level by correlation analysis with oxidative stress markers that may reflect the inflammatory processes in the oral cavity sampled on daily basis. Salivary markers of oxidative stress were found to be associated with several oral diseases including periodontitis, caries and oral precancerose.^41–43^ However, their usage for diagnostics of oral diseases is limited due to their large temporal variability in healthy subjects.^10,13^ The factors that determine this variability are largely unknown although microbes have been postulated as their modulators several years ago.^44,45^ The association of microbes with oxidative stress can only be tested in interventional experiments including long temporal sampling, because both, the microbes and immune cells, can induce the production of ROS and oxidative stress.^18,46^ In addition, potential systemic causes for the variability of both, oxidative stress markers in saliva and oral microbiome should not be omitted.^47^

In order to assess association of bacterial taxa with oxidative stress markers, we needed to detect temporal variability of the oral microbiome composition on the intra-individual level. In general, the temporal variability of the microbiome detected in our cohort of healthy volunteers (as measured by the Taylor’s parameter *V*) was lower than the variability detected in perturbed microbiomes previously studied using similar method.^29^ However, even slight temporal variations in the oral microbiome composition provided important data for intra-individual correlation analysis in this study. The design of the vast majority of microbiome studies does not take into account intra-individual temporal variability, which can be in fact very informative on the health status of the host.^29^ Our results are in accordance to the study of Gonze et al.^48^, which demonstrated that a microbiome may adopt one or another distinct state in the same environmental conditions, meaning that intra-individual temporal variability does not have to correlate necessarily with the changes of the environmental conditions. Interestingly, the intra-individual variability of the microbiome has been identified as the major cause of contradictory results reported by different biomedical studies.^49,50^ As shown in this study, an individual can have an unstable microbiome composition (expressed by the variability parameter *V*, Fig. 1), even being considered a healthy subject, thus a realistic picture of subject’s microbiome cannot be captured by collecting only one sample per individual. The computational simulation revealed that contradictory correlation analysis results may be obtained, if only one sample per volunteer is collected. For example, if single samples from the 26 volunteers are collected in 1000 different studies and analysed for correlations between *Rothia*.OTU15862 and TBARS, 22.2% of the studies would report a negative correlation, while 0.1% would report a positive correlation, and 77.7% would report no significant correlation. In comparison, our intra-individual correlation analysis found negative correlations in two volunteers and a positive correlation in one volunteer, while in the remaining volunteers the *Rothia*.OTU15862-TBARS pair resulted in no significant correlations. Another advantage of using correlation analysis on intra-individual level is in the possibility of identifying lagged correlations. The identification of lagged correlations was very important in the present study, as increased production of ROS may not necessarily start at the moment of overgrowth of a pathobiont, but it may be delayed by several days.

The detected correlation patterns between oxidative stress markers, between bacterial species and between markers and bacteria were unique for each volunteer. However, we also found some correlations which were highly consistent on intra-individual level in our cohort, e.g., negative correlation between *Streptococcus*-OTU0 and *Rothia*-OTU1, positive correlation between FRAS and TAC, etc. In addition, the Lotka-Volterra set of equations has proved the uniqueness of each volunteer interaction matrix. Interaction-based models, such as Lotka-Volterra equations, are more robust to extract biologically relevant interactions in the ecosystem than correlation models.^31^

The set of low-abundance species (0.1–5.4% average proportion), which were mostly individual-specific, contributed to the most of the microbiome temporal variability and correlated with the oxidative stress markers more often than the more prevalent species (such as *Rothia* and *Streptotoccus parasanguinis*). For example, *S. mutans* was one of the less prevalent species and it had significant correlations with the oxidative stress markers in the highest number of volunteers in our cohort. However, these correlations were not confirmed in all volunteers. Though, *S. mutans* is capable of producing ROS in vitro,^51,52^ its activity may be hampered by other species in the oral microbial community. It is possible that other bacterial species may inhibit *S. mutans* ROS production, particularly in the samples in which *S. mutans* did not correlate with oxidative stress markers. In addition, these volunteers are likely to contain other bacterial species that stimulate ROS production or benefit from ROS production to increase their biological niche in anoxic regions.^53,54^ Nevertheless, *S. mutans* was the bacterial species with one of the most pronounced correlations with the oxidative stress markers which supports its important role in caries pathogenesis.

Correlation patterns of bacterial species with oxidative stress markers differed among volunteers which may be explained by numerous factors. First of all, cells belonging to the same bacterial species distributed in different compartments of the oral cavity differ by their activity—some cells are actively metabolizing nutrients, while others are waiting for optimal growth conditions in a dormant stage.^55,56^ However, differential activity of bacterial cells is not taken into account when total DNA from saliva is sequenced which might explain why intra-individual correlation patterns of bacterial OTUs with oxidative stress markers were non-uniform in this study. Laboratory experiments performed with pure cultures containing uniformly growing bacterial cells might not fully mimic the real in vivo ROS production due to the differential activity of bacterial cells in the oral cavity. Furthermore, it was demonstrated that only a portion of bacterial cells belonging to the same bacterial species stimulate human immune reactions.^57,58^ Therefore, only a portion of each bacterial species is involved in the induction of the ROS production and these portions are likely inconsistent among different individuals. Although highly active bacterial cells and those which interact with the human immune system could be quantified using flow cytometry,^59,60^ it is very difficult to determine the exact number of variables that may influence the actual proportion of bacterial cells stimulating the ROS production in vivo. Having absolute number of cells belonging to each bacterial species, rather than compositional data, would help to address correlation patterns in each volunteer with more details.

In our study, many bacterial OTUs correlated either positively or negatively with some of the measured oxidative stress markers in some of the volunteers, which suggests that each volunteer has different homeostatic mechanisms. Therefore, there is no universal answer to which specific bacterial species are associated to the ROS production in the oral cavity in all humans. Integrating microbiome composition data with proteomics and metabolomics may help in determining which bacteria are associated to the ROS production in the oral cavity, as different bacterial species are likely to be responsible for the same metabolic function in different individuals.^61,62^ In such studies, information on microbiome temporal variability would be also of very high importance. In addition, future experimental studies should focus on the origin and the consequences of oxidative stress in the oral cavity in relation to the microbiome composition and its modulation or transplantation.

In conclusion, longitudinal sample collections allows to capture a realistic picture of subject’s microbiome, which may be relatively unstable even in good health conditions. Correlation analyses may produce contradictory results when performed with combinations of single samples collected from the same volunteers on different days. In contrast, longitudinal sample collections allow to calculate temporally direct and lagged correlation of bacterial taxa with distinct biochemical markers on intra-individual level. Not only marker-bacteria correlations but also the species-species correlations patterns can be unique for each volunteer. In general, the less prevalent species are mostly individual-specific and they contribute to the most of the microbiome temporal variability.

Correlation analysis showed that there are different species associated with oxidative stress in each human individual, rather than a universal single bacterial organism. Despite some promising correlations (e.g., the positive correlation between FRAS and *Streptococcus mutans* and negative correlations in the pairs AGE-*Streptococcus mutans*, AOPP-*Actinomyces*, AOPP-*Cardiobacterium* and FRAS-*Cardiobacterium*), the findings were not universal to all individuals. ROS production in the oral cavity should be further investigated in more details, e.g., including animal models, because correlation analyses do not provide direct evidence that a species induces inflammation.

The results of this study highlight the importance of longitudinal sample collection, especially in studies where correlations with oral microbiota composition are the key outcomes. In the future, the decreasing cost of DNA sequencing will allow to analyze multiple samples from each patient, extending potential diagnostic applications, and also understanding of microbiome-associated oral diseases.

## Methods

### Ethics approval and consent to participate

The ethical clearance was obtained from The Ethical Committee of the Faculty of Medicine, Comenius University in Bratislava, Slovakia. An informed written consent was obtained from each participant prior to the study.

### Subjects and sampling

Unstimulated whole mouth saliva was collected from 26 volunteers (13 males and 13 females) recruited from students of the Comenius University in Bratislava, Slovakia. Exclusion criteria included active periodontitis, untreated caries, oral pain or any known oral diseases, but also smoking and major chronic systemic diseases. Subjects taking any medications including over the counter available antioxidants were excluded as well. At least 2 ml of saliva was taken by spitting into sterile tubes daily for 30 days at the same time between 06:00 and 08:00 at least 30 min after toothbrushing. The volunteers were instructed not to eat in the morning before saliva collection. All samples were immediately frozen after collection and stored at −20 °C until further processing.

### 16S rRNA gene sequencing

DNA extracted from all salivary samples was extracted using a modified phenol chloroform protocol as described previously,^63,64^ and V3 and V4 regions of the 16S rRNA gene were amplified using following primers: forward primer TCG TCG GCA GCG TCA GAT GTG TAT AAG AGA CAG CCT ACG GGN GGC WGC AG, reverse primer GTC TCG TGG GCT CGG AGA TGT GTA TAA GAG ACA GGA CTA CHV GGG TAT CTA ATC C^65^) and Kapa HiFi Hot Sart polymerase (Ref. 7958935001). The PCR conditions were the following: 95 °C for 30 s, 55 °C for 30 s, 72 °C for 30 s repeated in 25 cycles. The samples were multiplexed by 96 index combination and sequenced on the Illumina platform with MiSeq Reagent Kit v3 (600-cycle, Ref. MS-102-3003). Quality assessment of raw paired ends data has been carried out using *prinseq-lite* program: sequences shorter than 50 bp were removed and then we applied a 3′ (right-hand) trimming up to a minimum quality mean value of 30 in a sliding window of 20 nucleotides.^66^ The obtained trimmed paired-end reads were merged using *fastq-join* program from *ea-utils* package^67^ and chimeric amplicons were removed by *usearch* program.^68^ In addition, the samples with less than 2500 sequences were removed from the analysis.

In the first step, we determined which are the most suitable settings for clustering of sequences into operational taxonomic units (OTUs) for this study. Clustering on 97% sequence similarity level is widely used for obtaining OTUs on species-like level, however, the 97% threshold is not universal for all species. Some species can be split into multiple OTUs, while hybrid OTUs containing multiple species can be formed as well.^69^ The testing of the most optimal clustering threshold in this study was crucial for distinguishing between caries-associated *Streptococcus mutans*^4^ and commensal *Streptococcus* species not associated with caries.^70^ For this purpose, all 16S rDNA sequences belonging to the genus *Streptococcus* were downloaded from the Ribosomal Database Project website (RDP, January 2016), clipped for the region V3 and V4 (regions which are used for sequencing in this study) and the clustering on different similarity levels (0.95, 0.96, 0.97, 0.98, 0.99) was tested using the *usearch* program. The similarity level 0.96 produced homogeneous clusters (OTUs) corresponding to separate *Streptococcus* species.

Afterwards, all obtained sequences from the 26 volunteers have been clustered together on 0.96 similarity level by the *usearch* program and the reference sequences of each OTU cluster have been taxonomically assigned by RDP classifier;^71^ the annotation was accepted if the bootstrap confidence estimation value was over 0.8.

### Analysis of the bacterial composition

The resulting bacterial composition was analysed in the R programming environment using packages “vegan”^72^ and “ade4”.^73^ As the clustering of large datasets into OTUs often results in formation of artificial low abundant OTUs corresponding to sequencing errors,^74^ the dataset containing all detected OTUs was compared to the datasets containing only OTUs with average proportion >0.001, and >0.1%. In the datasets with reduced OTUs numbers, the low prevalent OTUs were discarded. The Procrustes test (R library vegan, function “protest”) was performed to test whether the datasets with different OTU numbers produce the same ordination of samples in the nonmetric multidimensional scaling (NMDS) with Bray-Curtis dissimilarity.

The Shannon diversity index, Pielou’s evenness index values were compared among volunteers using t-test with Holm correction of p-values using the R package “vegan”.

In the next step, the “envfit” function from “vegan” R package was used for testing of the bacterial composition (OTUs with average proportion >0.1%) for fitting on variable “volunteer” in the canonical correspondence analysis (CCA). This analysis was used to test whether each volunteer has its own characteristic microbiome and whether the microbiomes differ significantly between volunteers.

### Temporal variations of the microbiome

The changes in the relative abundance of the OTUs were fitted to a power law with two parameters that described the microbiome stability over time, *σ* = *V* × *μβ*, where *V* and *β* are the parameters of the model, and *σ* and *μ* being the dispersion (in standard deviation) and the mean of the measurements.

The variability *V* was a direct estimator of the amplitude of fluctuations and therefore of the general stability, while the variable *β* described the statistical behavior of the ecosystem.^29^

Rank stability index (RSI) was calculated, per element, as 1 less the quotient of the number of true rank hops taken between the number of maximum possible rank hops, all powered to *p*:

1 $${\mathrm{RSI}} = \left( {1 - \frac{{{\mathrm{truerank}}\,{\mathrm{hops}}}}{{{\mathrm{possiblerank}}\,{\mathrm{hops}}}}} \right)^p = \left( {1 - \frac{D}{{\left( {N - 1} \right)\left( {t - 1} \right)}}} \right)^p,$$

where *D* is the total of rank hops taken by the studied element, *N* is the number of elements that have been ranked, and *t* is the number of time samples. p is arbitrarily chosen to increase the resolution in the desired region, for example the stable region.^29^

In addition, the rank variability (RV) and the differences variability (DV) were also calculated and plotted below RSI. The RV is the absolute difference between every taxon’s rank at a specific time point, and its accumulated abundance rank averaged for all the taxa shown. The DV is the absolute difference between every taxon’s rank at a specific time point compared to the previous time point. RV is a direct estimator of global changes, and DV is informing about local changes in the rank stability. All the analyses concerning the temporal variability of intra-individual microbiome are described in the work of Marti et al.^29^

### Analysis of oxidative stress and antioxidative status

The marker of lipid peroxidation, thiobarbituric acid reactive substances (TBARS), was assessed according to Behuliak et al.^10^ Twenty microliter of samples and standards (1, 1, 3, 3—tetraethoxypropane) were added into a 96-well plate. Thereafter, 30 µl of water, 20 µl of thiobarbituric acid together with 20 µl of glacial acetic acid were added and whole mixture was incubated at 95 °C for 45 min. Afterwards, 100 µl of n–butanol was added, and plate was centrifuged at 2000×*g*, at 4 °C for 10 min. Seventy microliter of the upper organic phase was transferred into a new microtiter plate and, subsequently, the fluorescence was measured at ex = 515 nm and em = 535 nm.

Advanced oxidation protein products (AOPP) were measured as a marker of protein damage.^75^ Chloramine T mixed with potassium iodide was used for preparation of the calibration curve. Two hundred µl of samples or standard were transferred onto a 96 well plate for analysis. Later, 20 µl glacial acetic acid was added to both standards and samples and incubated on a plate shaker (500 rpm) for 2 min. Then absorbance was measured at 340 nm.

Advanced glycation end products (AGEs) were assessed as a marker of carbonyl stress.^76^ Twenty microliter of samples together with standards (AGE-BSA) were placed into a 96-well plate and diluted with phosphate buffer saline (pH = 7.2). The fluorescence was measured at ex. = 370 nm and em. = 440 nm.

The analysis of antioxidant status was represented by the assessment of the total antioxidant capacity (TAC) according to Erel et al.^77^ Twenty microliter of samples and standards (Trolox) were mixed with 200 µl of acetate buffer (pH = 5.8) and measured at 660 nm as a blank. Thereafter, 20 µl of 2, 2 azino—bis (ethylbenzthiazoline—6—sulfonic acid) was added. Plate was incubated at room temperature for 5 min and the absorbance at 660 nm was measured.

Ferric reducing antioxidant power of saliva (FRAS) was the next marker of antioxidant status. Ferrous sulfate was used as a standard for the construction of the calibration curve.^78^ Two hundred µl of prewarmed (37 °C) FRAP reagent, composed of tripyridyl—s—triazine, FeCl_3_ × 6H_2_O, acetate buffer (pH = 3.6) and water, was added to the assay plate. Initial absorbance at 593 nm was measured as a blank. Thereafter, the samples and standards were added and the absorbance was measured at 593 nm.

### Correlations between salivary markers and microbiota

Pearson’s and Spearman’s temporally direct correlations and local similarity analysis for lagged correlations were used to assess correlations between bacterial OTUs in oral cavity of each volunteer individually taking into account the temporal dimension. Local Similarity Analysis checks the existence of a delay in the correlations between all possible combinations of variables. Pearson’s, Spearman’s, and LSA correlation coefficients were computed using eLSA Python package,^79^ with default parameters. This package computes permutation tests that are helpful when multiple hypothesis tests are being studied in this kind of problems. The correlation values > 0.3 and < −0.3 were then filtered by their *p*-value at a level of 0.05 for statistical significance. Correlations in this study have been plotted using R packages “corrplot”^80^ and “beeswarm”.^81^

In addition, interactions between the 15 most abundant OTUs per sample were calculated with LIMITS algorithm^31^ in Wolfram Mathematica software (version 11.0). This algorithm looks for interactions under the generalized Lotka-Volterra model of ecological interactions, as they are a robust manner of looking for interdependence between species that has biological implications. One of the strengths of this algorithm is that it forces sparsity in the generated matrices, looking for interaction matrices that are similar to the real biological communities. We only used the 15 most abundant species per sample as one of the caveats of this kind of analysis is that it needs a great number of time-points for studying the interaction of only a few species, due to overfitting problems.

### Longitudinal sample collection vs. single sample collection

In most of the biomedical studies, only one sample per volunteer is collected and tested for correlations with its corresponding biomedical data. Such an approach ignores the temporal intra-individual variability of the microbiome composition and may lead to contradictory results obtained by different research groups.^48^ To test our data, we simulated such an one-sample-one-volunteer approach and compared it with the result of the intra-individual correlation analysis based on our longitudinal sample collection described above. For that purpose, only one sample per each volunteer was selected randomly for a total 1000 combinations and then tested for Pearson’s correlations between salivary markers and OTUs proportions. The correlation values > 0.3 and < −0.3 were also filtered by their *p*-value at a level of 0.05 for statistical significance.

## Electronic supplementary material

Supplemental Material

## Data Availability

The obtained sequences were deposited to the EBI database with the study accession number: PRJEB20213 (ERP022351).

## Electronic supplementary material

**Supplementary information** accompanies the paper on the *npj Biofilms and Microbiomes* website (10.1038/s41522-018-0072-3).
