# Supplementary material for: Oxidative stress in the oral cavity is driven by individual-specific bacterial communities
Source: NPJ Biofilms Microbiomes. 2018 Nov 27;4:29. doi: 10.1038/s41522-018-0072-3 (PMC6258756; doi:10.1038/s41522-018-0072-3)
Supplement: Supplementary file 1 — Supplemental Material [file 41522_2018_72_MOESM1_ESM.pdf]

Supplementary Information for:

Oxidative stress in the oral cavity is driven by individual-specific bacterial communities

Authors:

Mária Džunková, Daniel Martinez-Martinez, Roman Gardlík , Michal Behuliak, Katarína Janšáková, Nuria Jiménez, Jorge F. Vázquez-Castellanos, Jose Manuel Martí, Giuseppe D'Auria, H.M.H.N. Bandara, Amparo Latorre, Peter Celec, Andrés Moya

This Additional Files supplement includes:

Supplementary Figure S1...page 2

Supplementary Figure S2...page 3

Supplementary Figure S3...page 4

Supplementary Figure S4...page 4

Supplementary Figure S5...pages 4-28

Supplementary Figure S6...page 29

Supplementary Figure S7..pages 30

Supplementary Figure S8..pages 31-35

Supplementary Figure S1:

Composition of the salivary microbiome of the 26 volunteers. Boxplots show the proportion of the eight most prevalent bacterial OTUs. The box represents middle 50% of scores which fall within the inter-quartile range, while median marks the mid-point.

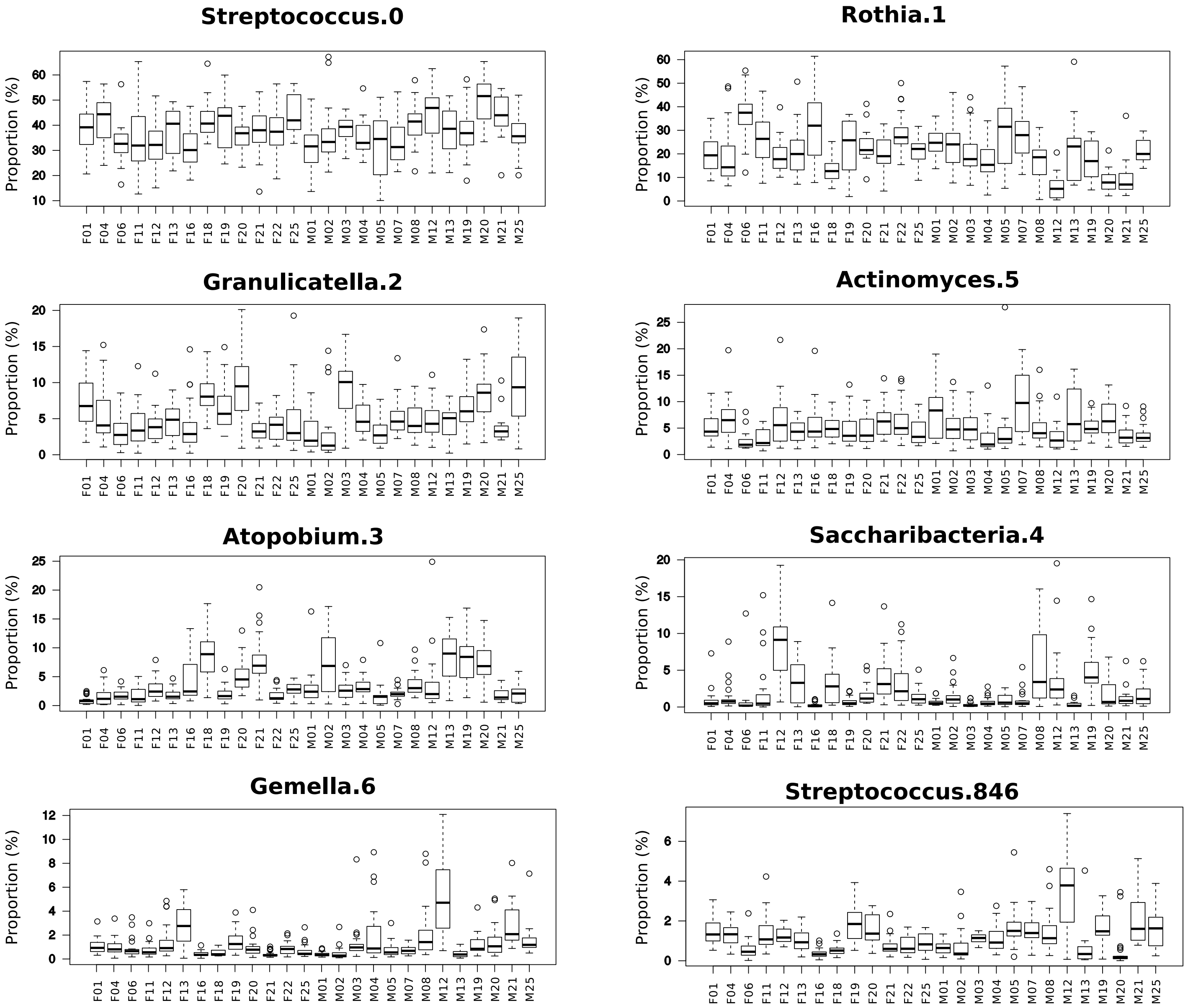

Supplementary Figure S2:

In the canonical correspondence analysis plot, the bacterial composition of the 551 samples was tested to assess the fit with the “volunteer individuality” variable. All OTUs with average proportion > 0.001 % were included in the analysis. Samples from each volunteer are colored as indicated the figure legend. The graphics shows only those of the 50 most prevalent OTUs which had significant influence (p > 0.001) on the ordination of samples.

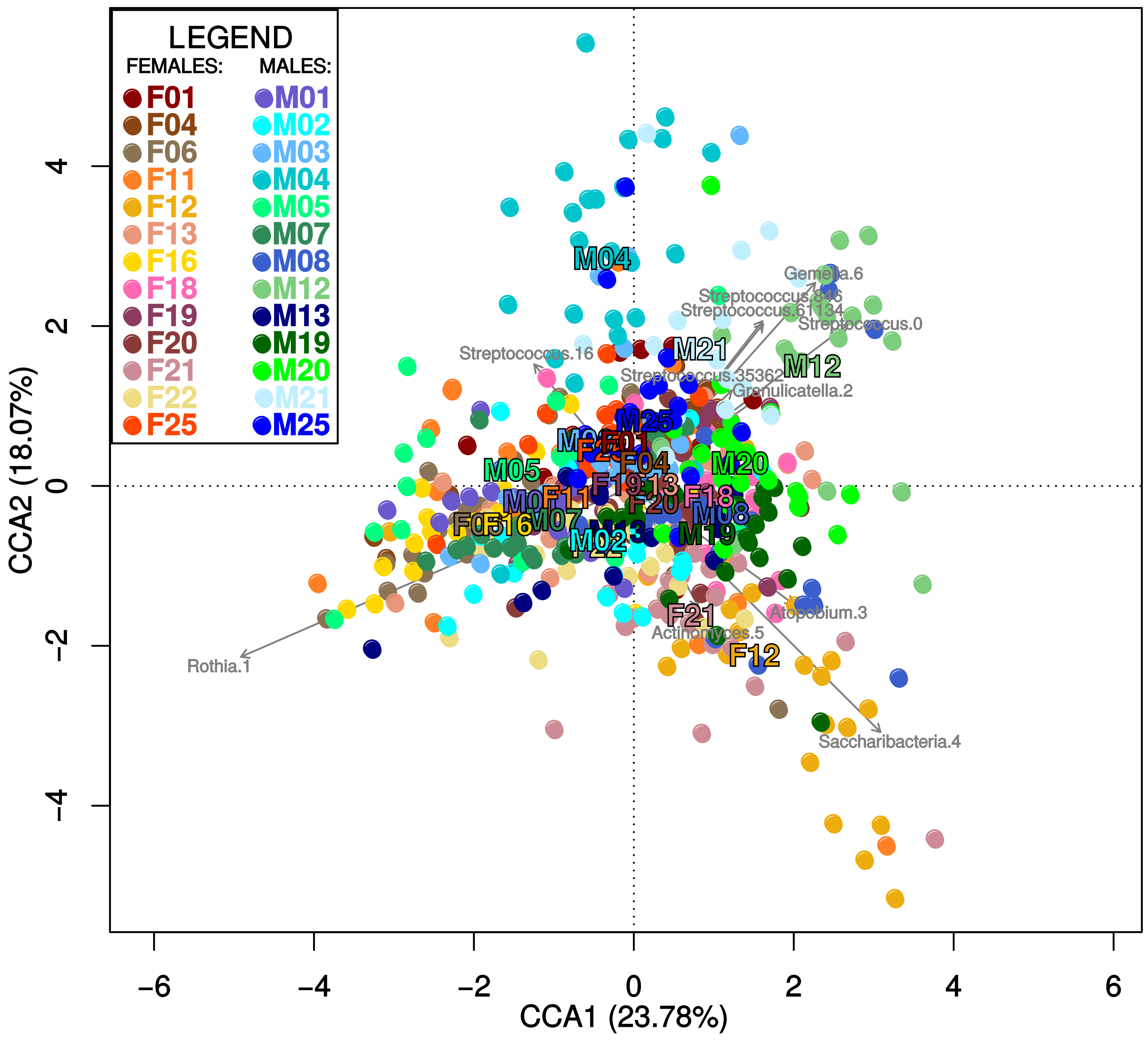

Supplementary Figure S3:

Bixplots showing Shannon diveristy index and evenness index for each of the volunteers. The box represents middle 50% of scores which fall within the inter-quartile range, while median marks the mid-point.

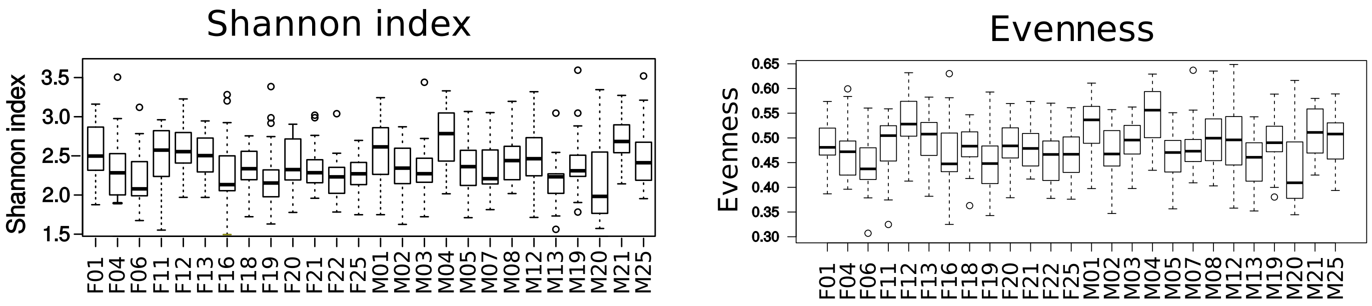

Supplementary Figure S4:

Comparison of NMDS ordinations of the 551 saliva samples including all 18,146 OTUs, the OTUs with average proportion > 0.001 % (687 OTUs) and the OTUs with average proportion > 0.1 % (50 OTUs). These three ordination did not result to be significantly different (p<0.001).

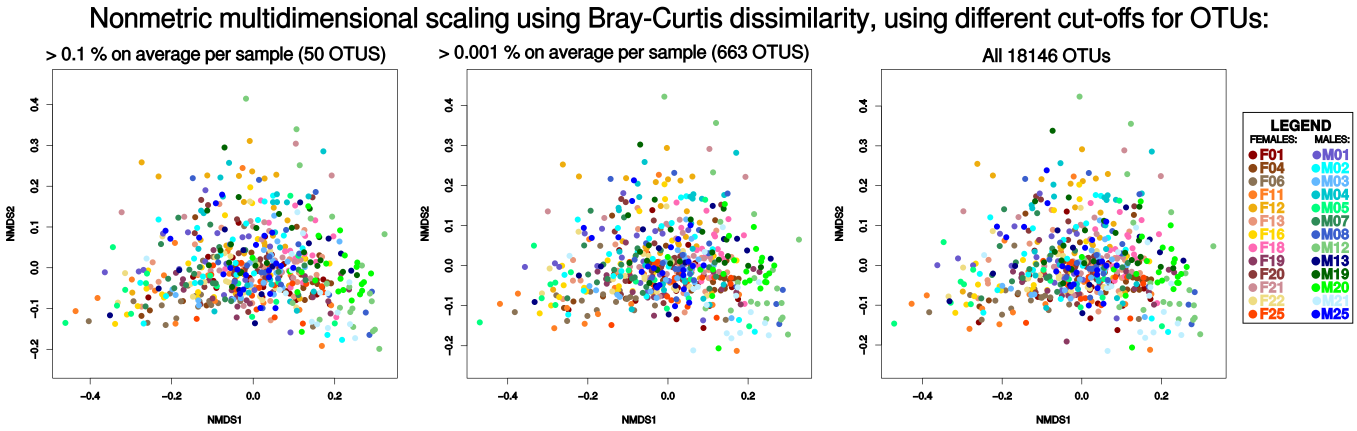

Supplementary Figure S5:

Temporal variability of 50 most prevalent OTUs in the 24 volunteers. The volunteers F18 and F11 (the most and the less stable) are shown in the Figure 2.

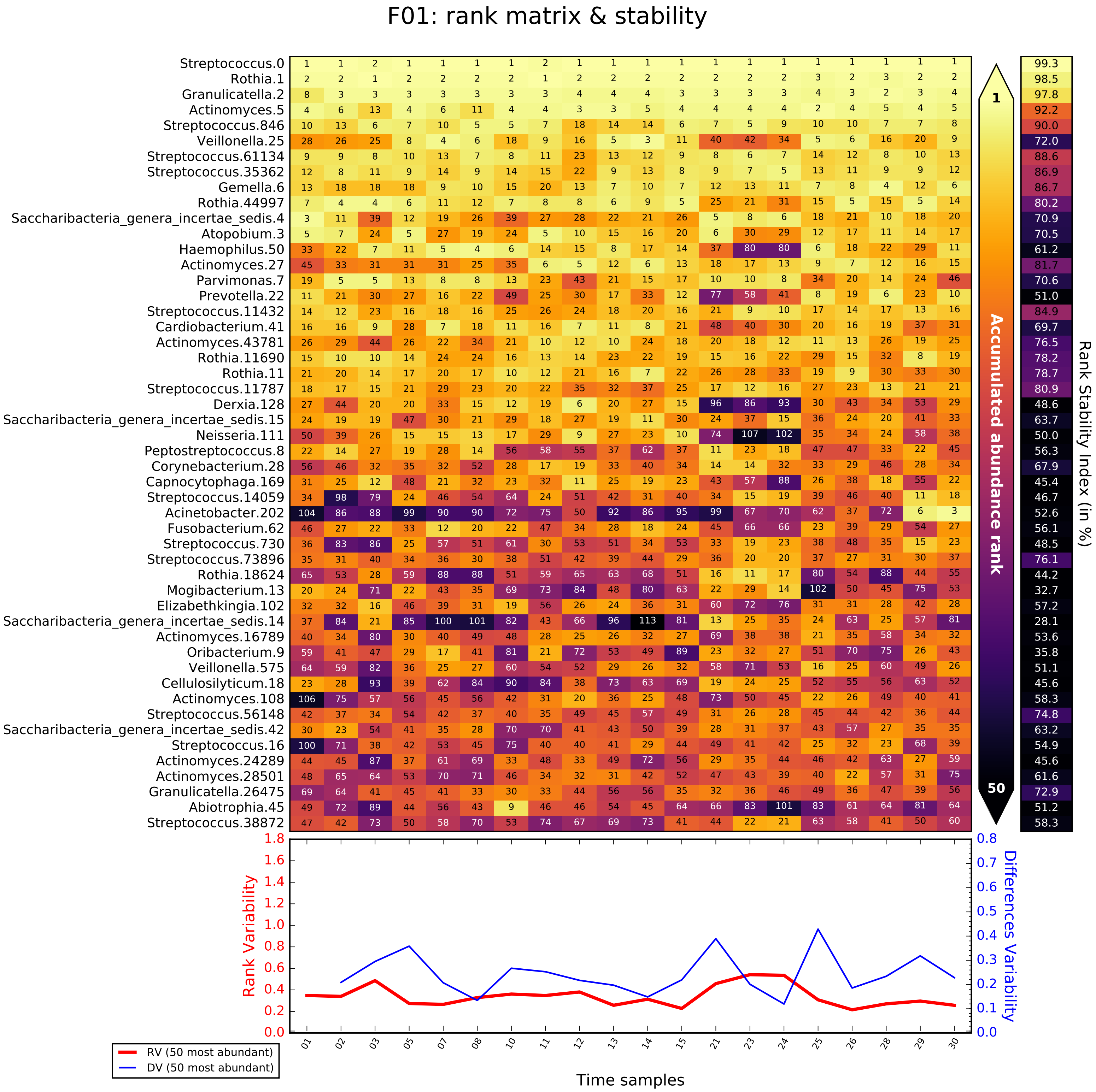
F01: rank matrix & stability
Streptococcus.0
Rothia.1
Granulicatella.2
Actinomyces.5
Streptococcus.846
Veillonella.25
Streptococcus.61134
Streptococcus.35362
Gemella.6
Rothia.44997
Saccharibacteria_genera_incertae_sedis.4
Atopobium.3
Haemophilus.50
Actinomyces.27
Parvimonas.7
Prevotella.22
Streptococcus.11432
Cardiobacterium.41
Actinomyces.43781
Rothia.11690
Rothia.11
Streptococcus.11787
Derxia.128
Saccharibacteria_genera_incertae_sedis.15
Neisseria.111
Peptostreptococcus.8
Corynebacterium.28
Capnocytophaga.169
Streptococcus.14059
Acinetobacter.202
Fusobacterium.62
Streptococcus.730
Streptococcus.73896
Rothia.18624
Mogibacterium.13
Elizabethkingia.102
Saccharibacteria_genera_incertae_sedis.14
Actinomyces.16789
Oribacterium.9
Veillonella.575
Cellulosilyticum.18
Actinomyces.108
Streptococcus.56148
Saccharibacteria_genera_incertae_sedis.42
Streptococcus.16
Actinomyces.24289
Actinomyces.28501
Granulicatella.26475
Abiotrophia.45
Streptococcus.38872
Accumulated abundance rank
1
50
Rank Stability Index (in %)
99.3
98.5
97.8
92.2
90.0
72.0
88.6
86.9
86.7
80.2
70.9
70.5
61.2
81.7
70.6
51.0
84.9
69.7
76.5
78.2
78.7
80.9
48.6
63.7
50.0
56.3
67.9
45.4
46.7
52.6
56.1
48.5
76.1
44.2
32.7
57.2
28.1
53.6
35.8
51.1
45.6
58.3
74.8
63.2
54.9
45.6
61.6
72.9
51.2
58.3
Rank Variability
Differences Variability
RV (50 most abundant)
DV (50 most abundant)
01
02
03
05
07
08
10
11
12
13
14
15
21
23
24
25
26
28
29
30
Time samples

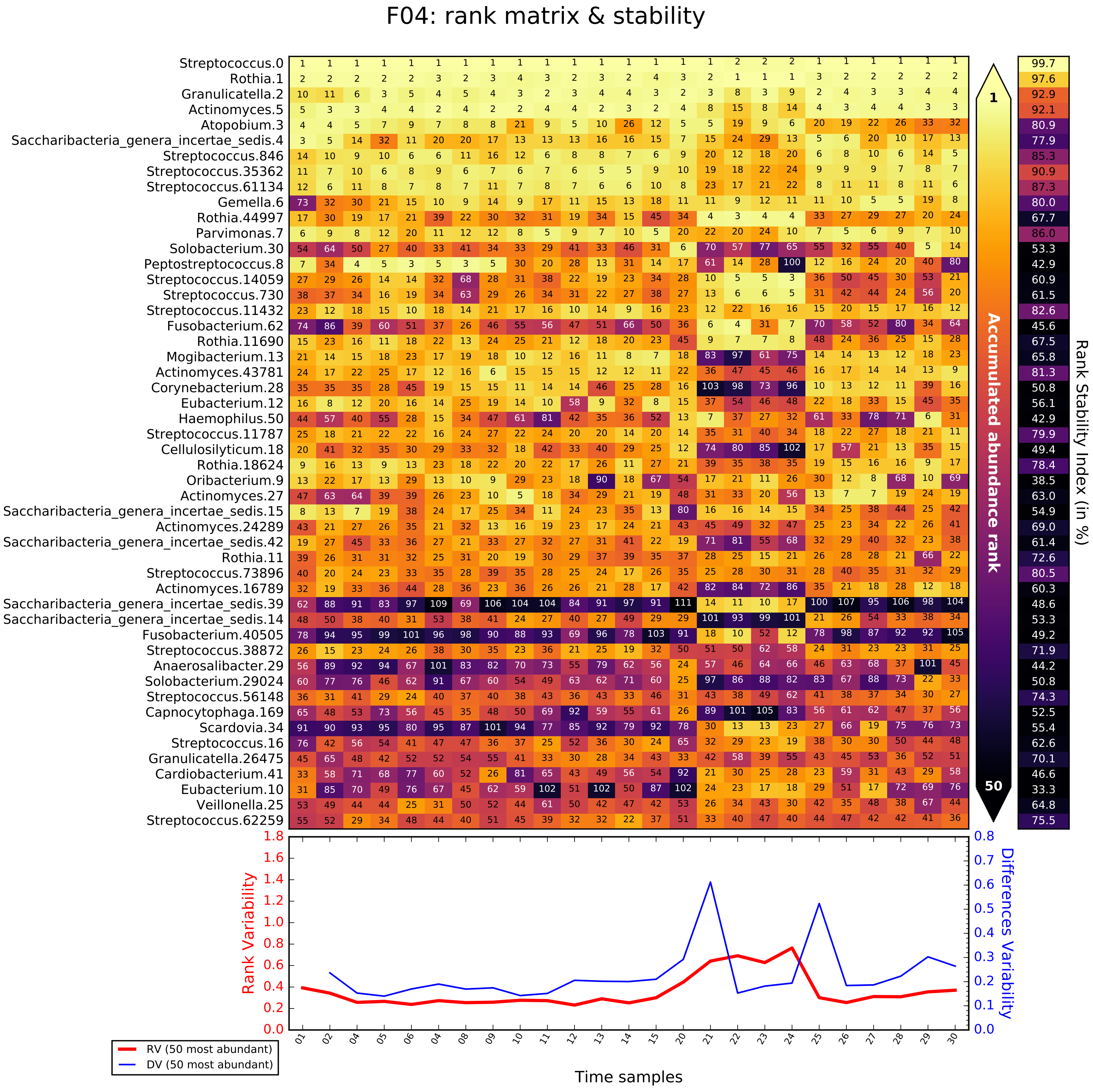
F04: rank matrix & stability
Streptococcus.0
Rothia.1
Granulicatella.2
Actinomyces.5
Atopobium.3
Saccharibacteria_genera_incertae_sedis.4
Streptococcus.846
Streptococcus.35362
Streptococcus.61134
Gemella.6
Rothia.44997
Parvimonas.7
Solobacterium.30
Peptostreptococcus.8
Streptococcus.14059
Streptococcus.730
Streptococcus.11432
Fusobacterium.62
Rothia.11690
Mogibacterium.13
Actinomyces.43781
Corynebacterium.28
Eubacterium.12
Haemophilus.50
Streptococcus.11787
Cellulosilyticum.18
Rothia.18624
Oribacterium.9
Actinomyces.27
Saccharibacteria_genera_incertae_sedis.15
Actinomyces.24289
Saccharibacteria_genera_incertae_sedis.42
Rothia.11
Streptococcus.73896
Actinomyces.16789
Saccharibacteria_genera_incertae_sedis.39
Saccharibacteria_genera_incertae_sedis.14
Fusobacterium.40505
Streptococcus.38872
Anaerosalibacter.29
Solobacterium.29024
Streptococcus.56148
Capnocytophaga.169
Scardovia.34
Streptococcus.16
Granulicatella.26475
Cardiobacterium.41
Eubacterium.10
Veillonella.25
Streptococcus.62259
Accumulated abundance rank
1
50
Rank Stability Index (in %)
Rank Variability
Differences Variability
RV (50 most abundant)
DV (50 most abundant)
Time samples

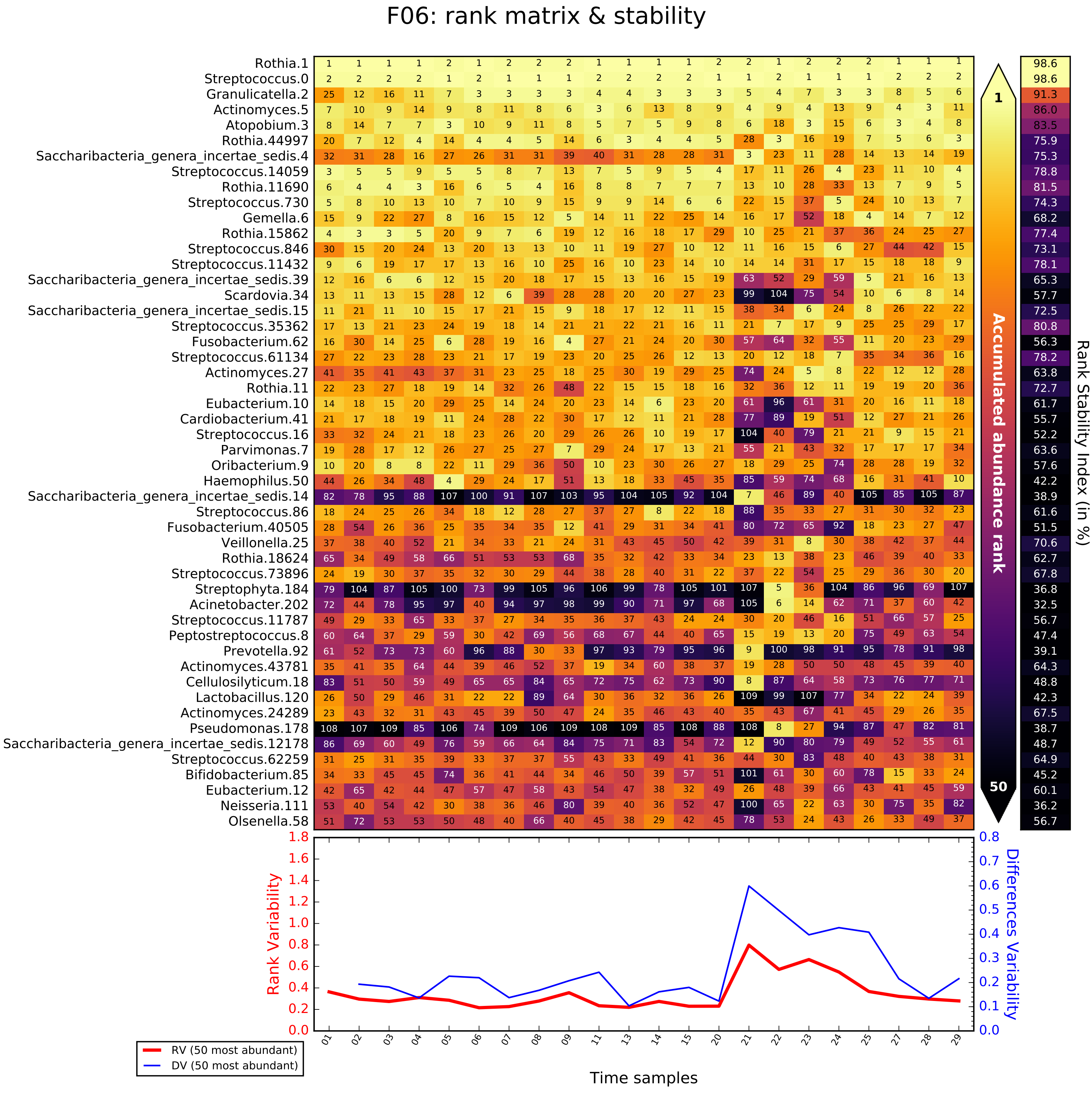

F06: rank matrix & stability
Rothia.1
Streptococcus.0
Granulicatella.2
Actinomyces.5
Atopobium.3
Rothia.44997
Saccharibacteria_genera_incertae_sedis.4
Streptococcus.14059
Rothia.11690
Streptococcus.730
Gemella.6
Rothia.15862
Streptococcus.846
Streptococcus.11432
Saccharibacteria_genera_incertae_sedis.39
Scardovia.34
Saccharibacteria_genera_incertae_sedis.15
Streptococcus.35362
Fusobacterium.62
Streptococcus.61134
Actinomyces.27
Rothia.11
Eubacterium.10
Cardiobacterium.41
Streptococcus.16
Parvimonas.7
Oribacterium.9
Haemophilus.50
Saccharibacteria_genera_incertae_sedis.14
Streptococcus.86
Fusobacterium.40505
Veillonella.25
Rothia.18624
Streptococcus.73896
Streptophyta.184
Acinetobacter.202
Streptococcus.11787
Peptostreptococcus.8
Prevotella.92
Actinomyces.43781
Cellulosilyticum.18
Lactobacillus.120
Actinomyces.24289
Pseudomonas.178
Saccharibacteria_genera_incertae_sedis.12178
Streptococcus.62259
Bifidobacterium.85
Eubacterium.12
Neisseria.111
Olsenella.58
Accumulated abundance rank
1
50
Rank Stability Index (in %)
98.6
98.6
91.3
86.0
83.5
75.9
75.3
78.8
81.5
74.3
68.2
77.4
73.1
78.1
65.3
57.7
72.5
80.8
56.3
78.2
63.8
72.7
61.7
55.7
52.2
63.6
57.6
42.2
38.9
61.6
51.5
70.6
62.7
67.8
36.8
32.5
56.7
47.4
39.1
64.3
48.8
42.3
67.5
38.7
48.7
64.9
45.2
60.1
36.2
56.7
Rank Variability
Differences Variability
Time samples
01 02 03 04 05 06 07 08 09 11 13 14 15 20 21 22 23 24 25 27 28 29
RV (50 most abundant)
DV (50 most abundant)

F12: rank matrix & stability

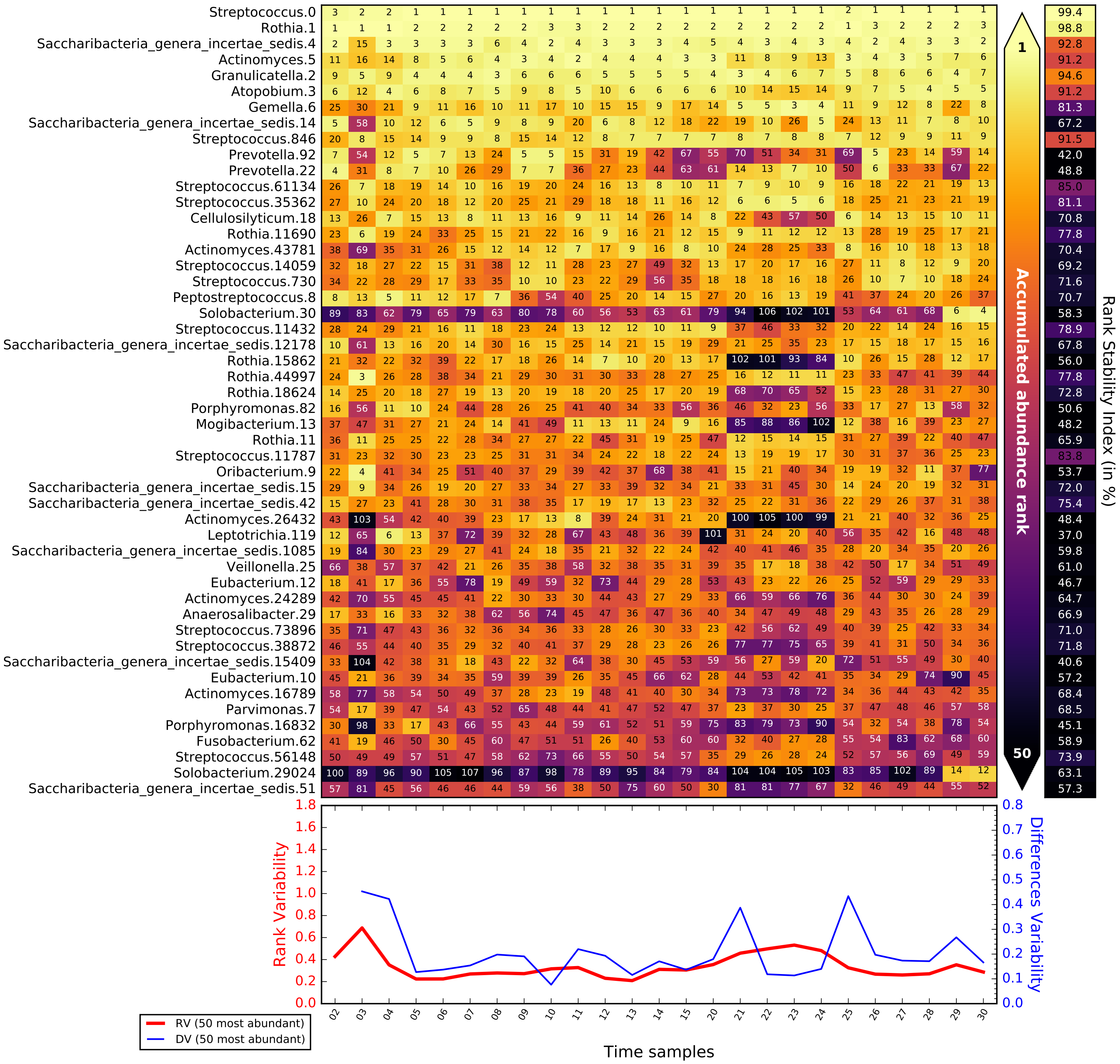

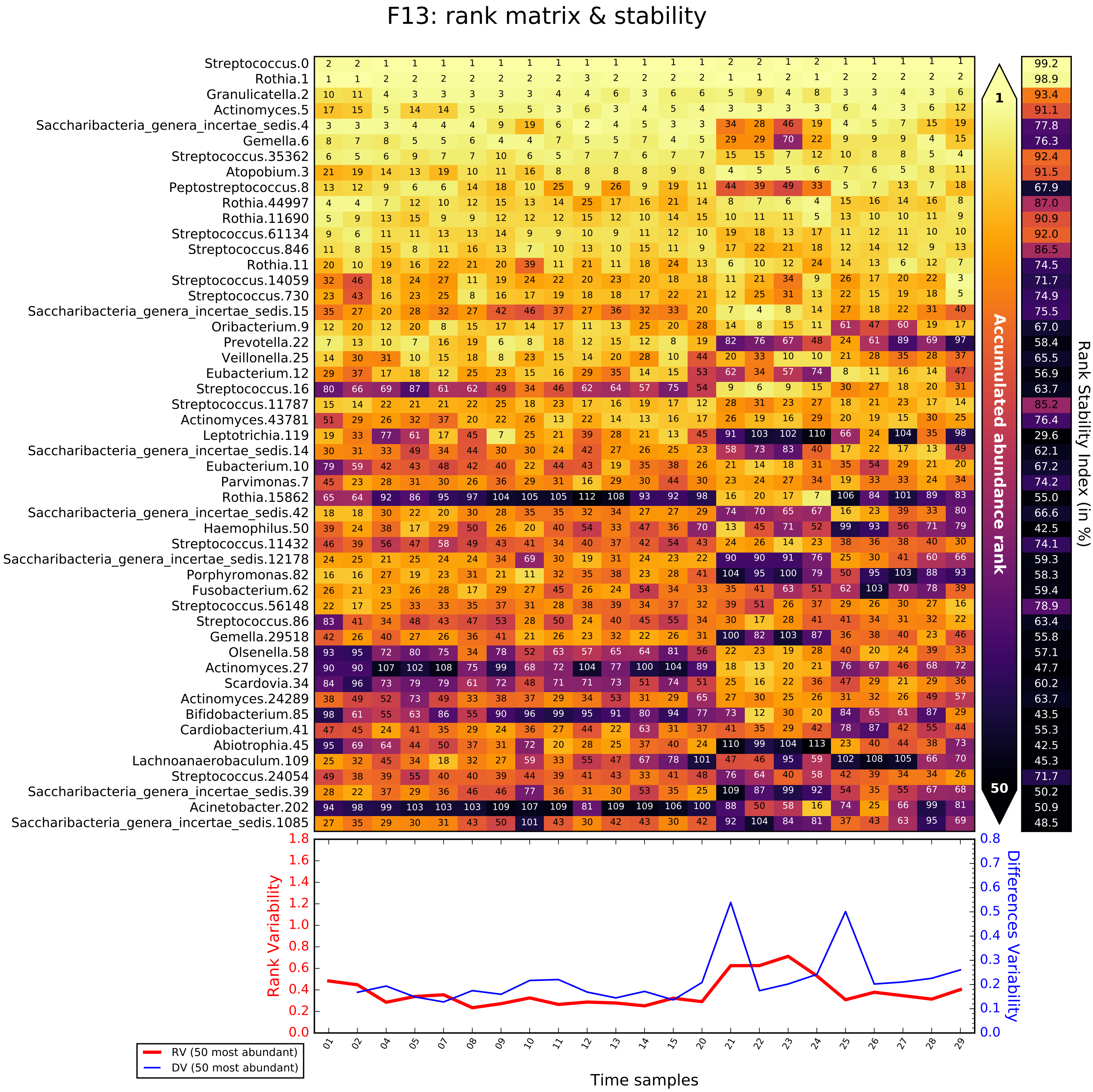

F13: rank matrix & stability
Streptococcus.0 2 2 1 1 1 1 1 1 1 1 1 1 1 1 2 2 1 2 1 1 1 1 1 99.2
Rothia.1 1 1 2 2 2 2 2 2 2 3 2 2 2 2 1 1 2 1 2 2 2 2 2 98.9
Granulicatella.2 10 11 4 3 3 3 3 3 4 4 6 3 6 6 5 9 4 8 3 3 4 3 6 93.4
Actinomyces.5 17 15 5 14 14 5 5 5 3 6 3 4 5 4 3 3 3 3 6 4 3 6 12 91.1
Saccharibacteria_genera_incertae_sedis.4 3 3 3 4 4 4 9 19 6 2 4 5 3 3 34 28 46 19 4 5 7 15 19 77.8
Gemella.6 8 7 8 5 5 6 4 4 7 5 5 7 4 5 29 29 70 22 9 9 9 4 15 76.3
Streptococcus.35362 6 5 6 9 7 7 10 6 5 7 7 6 7 7 15 15 7 12 10 8 8 5 4 92.4
Atopobium.3 21 19 14 13 19 10 11 16 8 8 8 8 9 8 4 5 5 6 7 6 5 8 11 91.5
Peptostreptococcus.8 13 12 9 6 6 14 18 10 25 9 26 9 19 11 44 39 49 33 5 7 13 7 18 67.9
Rothia.44997 4 4 7 12 10 12 15 13 14 25 17 16 21 14 8 7 6 4 15 16 14 16 8 87.0
Rothia.11690 5 9 13 15 9 9 12 12 12 15 12 10 14 15 10 11 11 5 13 10 10 11 9 90.9
Streptococcus.61134 9 6 11 11 13 13 14 9 9 10 9 11 12 10 19 18 13 17 11 12 11 10 10 92.0
Streptococcus.846 11 8 15 8 11 16 13 7 10 13 10 15 11 9 17 22 21 18 12 14 12 9 13 86.5
Rothia.11 20 10 19 16 22 21 20 39 11 21 11 18 24 13 6 10 12 24 14 13 6 12 7 74.5
Streptococcus.14059 32 46 18 24 27 11 19 24 22 20 23 20 18 18 11 21 34 9 26 17 20 22 3 71.7
Streptococcus.730 23 43 16 23 25 8 16 17 19 18 18 17 22 21 12 25 31 13 22 15 19 18 5 74.9
Saccharibacteria_genera_incertae_sedis.15 35 27 20 28 32 27 42 46 37 27 36 32 33 20 7 4 8 14 27 18 22 31 40 75.5
Oribacterium.9 12 20 12 20 8 15 17 14 17 11 13 25 20 28 14 8 15 11 61 47 60 19 17 67.0
Prevotella.22 7 13 10 7 16 19 6 8 18 12 15 12 8 19 82 76 67 48 24 61 89 69 97 58.4
Veillonella.25 14 30 31 10 15 18 8 23 15 14 20 28 10 44 20 33 10 10 21 28 35 28 37 65.5
Eubacterium.12 29 37 17 18 12 25 23 15 16 29 35 14 15 53 62 34 57 74 8 11 16 14 47 56.9
Streptococcus.16 80 66 69 87 61 62 49 34 46 62 64 57 75 54 9 6 9 15 30 27 18 20 31 63.7
Streptococcus.11787 15 14 22 21 21 22 25 18 23 17 16 19 17 12 28 31 23 27 18 21 23 17 14 85.2
Actinomyces.43781 51 29 26 32 37 20 22 26 13 22 14 13 16 17 26 19 16 29 20 19 15 30 25 76.4
Leptotrichia.119 19 33 77 61 17 45 7 25 21 39 28 21 13 45 91 103 102 110 66 24 104 35 98 29.6
Saccharibacteria_genera_incertae_sedis.14 30 31 33 49 34 44 30 30 24 42 27 26 25 23 58 73 83 40 17 22 17 13 49 62.1
Eubacterium.10 79 59 42 43 48 42 40 22 44 43 19 35 38 26 21 14 18 31 35 54 29 21 20 67.2
Parvimonas.7 45 23 28 31 30 26 36 29 31 16 29 30 44 30 23 24 27 34 19 33 33 24 34 74.2
Rothia.15862 65 64 92 86 95 97 104 105 105 112 108 93 92 98 16 20 17 7 106 84 101 89 83 55.0
Saccharibacteria_genera_incertae_sedis.42 18 18 30 22 20 30 28 35 35 32 34 27 27 29 74 70 65 67 16 23 39 33 80 66.6
Haemophilus.50 39 24 38 17 29 50 26 20 40 54 33 47 36 70 13 45 71 52 99 93 56 71 79 42.5
Streptococcus.11432 46 39 56 47 58 49 43 41 34 40 37 42 54 43 24 26 14 23 38 36 38 40 30 74.1
Saccharibacteria_genera_incertae_sedis.12178 24 25 21 25 24 24 34 69 30 19 31 24 23 22 90 90 91 76 25 30 41 60 66 59.3
Porphyromonas.82 16 16 27 19 23 31 21 11 32 35 38 23 28 41 104 95 100 79 50 95 103 88 93 58.3
Fusobacterium.62 26 21 23 26 28 17 29 27 45 26 24 54 34 33 35 41 63 51 62 103 70 78 39 59.4
Streptococcus.56148 22 17 25 33 33 35 37 31 28 38 39 34 37 32 39 51 26 37 29 26 30 27 16 78.9
Streptococcus.86 83 41 34 48 43 47 53 28 50 24 40 45 55 34 30 17 28 41 41 34 31 32 22 63.4
Gemella.29518 42 26 40 27 26 36 41 21 26 23 32 22 26 31 100 82 103 87 36 38 40 23 46 55.8
Olsenella.58 93 95 72 80 75 34 78 52 63 57 65 64 81 56 22 23 19 28 40 20 24 39 33 57.1
Actinomyces.27 90 90 107 102 108 75 99 68 72 104 77 100 104 89 18 13 20 21 76 67 46 68 72 47.7
Scardovia.34 84 96 73 79 79 61 72 48 71 71 73 51 74 51 25 16 22 36 47 29 21 29 36 60.2
Actinomyces.24289 38 49 52 73 49 33 38 37 29 34 53 31 29 65 27 30 25 26 31 32 26 49 57 63.7
Bifidobacterium.85 98 61 55 63 86 55 90 96 99 95 91 80 94 77 73 12 30 20 84 65 61 87 29 43.5
Cardiobacterium.41 47 45 24 41 35 29 24 36 27 44 22 63 31 37 41 35 29 42 78 87 42 55 44 55.3
Abiotrophia.45 95 69 64 44 50 37 31 72 20 28 25 37 40 24 110 99 104 113 23 40 44 38 73 42.5
Lachnoanaerobaculum.109 25 32 45 34 18 32 27 59 33 55 47 67 78 101 47 46 95 59 102 108 105 66 70 45.3
Streptococcus.24054 49 38 39 55 40 40 39 44 39 41 43 33 41 48 76 64 40 58 42 39 34 34 26 71.7
Saccharibacteria_genera_incertae_sedis.39 28 22 37 29 36 46 46 77 36 31 30 53 35 25 109 87 99 92 54 35 55 67 68 50.2
Acinetobacter.202 94 98 99 103 103 103 109 107 109 81 109 109 106 100 88 50 58 16 74 25 66 99 81 50.9
Saccharibacteria_genera_incertae_sedis.1085 27 35 29 30 31 43 50 101 43 30 42 43 30 42 92 104 84 81 37 43 63 95 69 48.5
Accumulated abundance rank
1
50
Rank Stability Index (in %)
Rank Variability
Differences Variability
1.8 1.6 1.4 1.2 1.0 0.8 0.6 0.4 0.2 0.0
0.8 0.7 0.6 0.5 0.4 0.3 0.2 0.1 0.0
01 02 04 05 07 08 09 10 11 12 13 14 15 20 21 22 23 24 25 26 27 28 29
RV (50 most abundant)
DV (50 most abundant)
Time samples

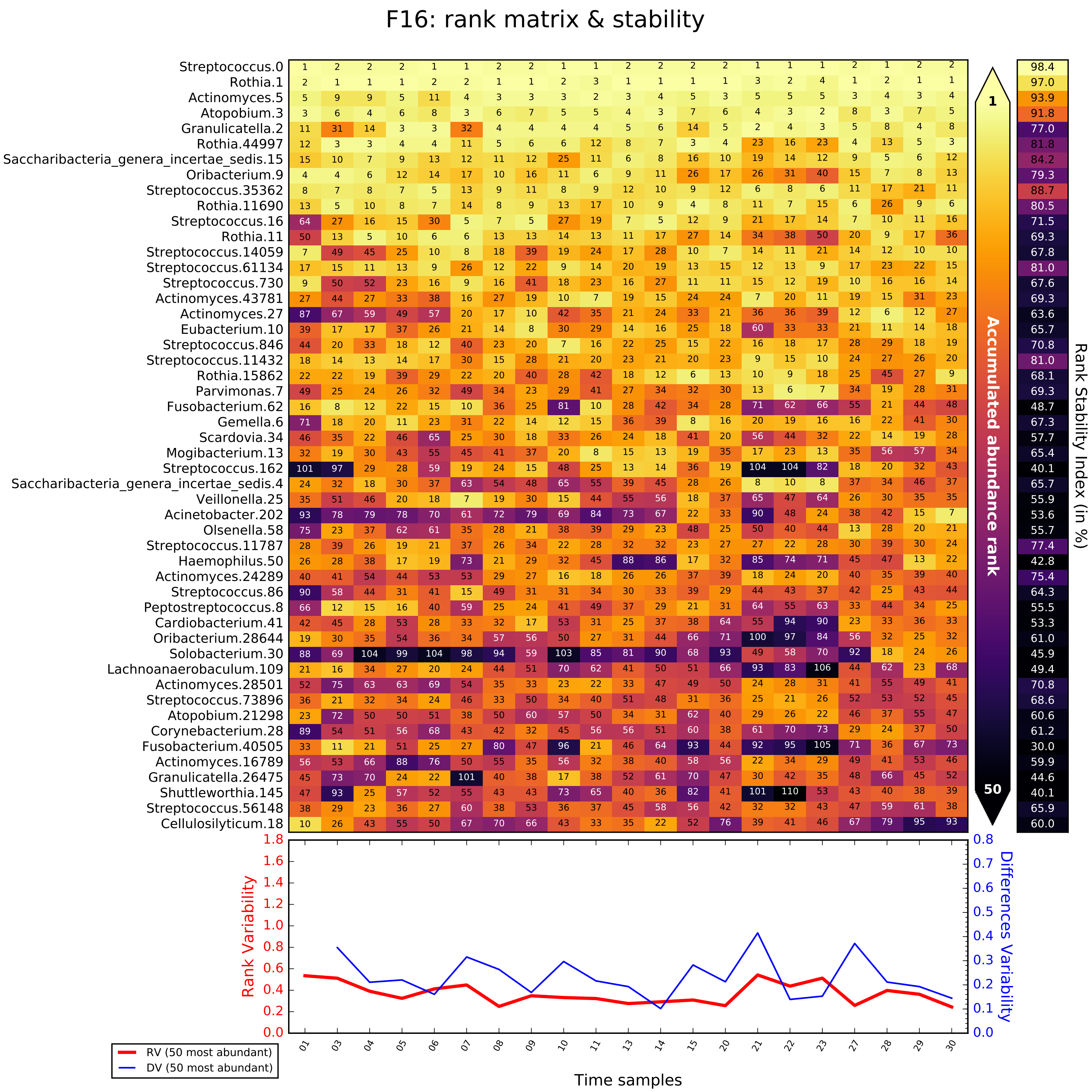
F16: rank matrix & stability
Streptococcus.0
Rothia.1
Actinomyces.5
Atopobium.3
Granulicatella.2
Rothia.44997
Saccharibacteria_genera_incertae_sedis.15
Oribacterium.9
Streptococcus.35362
Rothia.11690
Streptococcus.16
Rothia.11
Streptococcus.14059
Streptococcus.61134
Streptococcus.730
Actinomyces.43781
Actinomyces.27
Eubacterium.10
Streptococcus.846
Streptococcus.11432
Rothia.15862
Parvimonas.7
Fusobacterium.62
Gemella.6
Scardovia.34
Mogibacterium.13
Streptococcus.162
Saccharibacteria_genera_incertae_sedis.4
Veillonella.25
Acinetobacter.202
Olsenella.58
Streptococcus.11787
Haemophilus.50
Actinomyces.24289
Streptococcus.86
Peptostreptococcus.8
Cardiobacterium.41
Oribacterium.28644
Solobacterium.30
Lachnoanaerobaculum.109
Actinomyces.28501
Streptococcus.73896
Atopobium.21298
Corynebacterium.28
Fusobacterium.40505
Actinomyces.16789
Granulicatella.26475
Shuttleworthia.145
Streptococcus.56148
Cellulosilyticum.18
Accumulated abundance rank
1
50
Rank Stability Index (in %)
98.4
97.0
93.9
91.8
77.0
81.8
84.2
79.3
88.7
80.5
71.5
69.3
67.8
81.0
67.6
69.3
63.6
65.7
70.8
81.0
68.1
69.3
48.7
67.3
57.7
65.4
40.1
65.7
55.9
53.6
55.7
77.4
42.8
75.4
64.3
55.5
53.3
61.0
45.9
49.4
70.8
68.6
60.6
61.2
30.0
59.9
44.6
40.1
65.9
60.0
Rank Variability
Differences Variability
RV (50 most abundant)
DV (50 most abundant)
01 03 04 05 06 07 08 09 10 11 13 14 15 20 21 22 23 27 28 29 30
Time samples

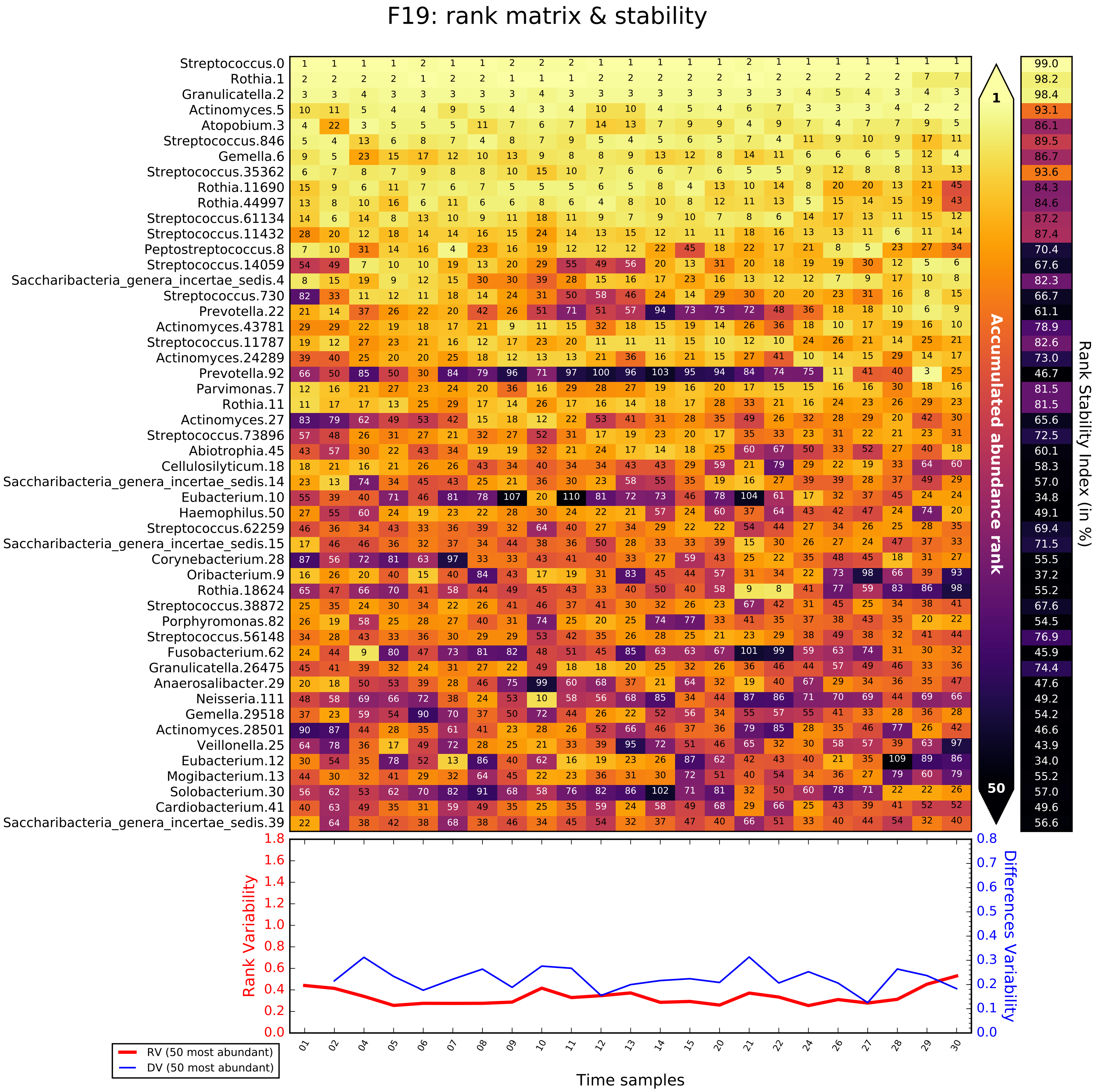
F19: rank matrix & stability
Streptococcus.0
Rothia.1
Granulicatella.2
Actinomyces.5
Atopobium.3
Streptococcus.846
Gemella.6
Streptococcus.35362
Rothia.11690
Rothia.44997
Streptococcus.61134
Streptococcus.11432
Peptostreptococcus.8
Streptococcus.14059
Saccharibacteria_genera_incertae_sedis.4
Streptococcus.730
Prevotella.22
Actinomyces.43781
Streptococcus.11787
Actinomyces.24289
Prevotella.92
Parvimonas.7
Rothia.11
Actinomyces.27
Streptococcus.73896
Abiotrophia.45
Cellulosilyticum.18
Saccharibacteria_genera_incertae_sedis.14
Eubacterium.10
Haemophilus.50
Streptococcus.62259
Saccharibacteria_genera_incertae_sedis.15
Corynebacterium.28
Oribacterium.9
Rothia.18624
Streptococcus.38872
Porphyromonas.82
Streptococcus.56148
Fusobacterium.62
Granulicatella.26475
Anaerosalibacter.29
Neisseria.111
Gemella.29518
Actinomyces.28501
Veillonella.25
Eubacterium.12
Mogibacterium.13
Solobacterium.30
Cardiobacterium.41
Saccharibacteria_genera_incertae_sedis.39
Accumulated abundance rank
1
50
Rank Stability Index (in %)
99.0
98.2
98.4
93.1
86.1
89.5
86.7
93.6
84.3
84.6
87.2
87.4
70.4
67.6
82.3
66.7
61.1
78.9
82.6
73.0
46.7
81.5
81.5
65.6
72.5
60.1
58.3
57.0
34.8
49.1
69.4
71.5
55.5
37.2
55.2
67.6
54.5
76.9
45.9
74.4
47.6
49.2
54.2
46.6
43.9
34.0
55.2
57.0
49.6
56.6
Rank Variability
Differences Variability
RV (50 most abundant)
DV (50 most abundant)
Time samples
01
02
04
05
06
07
08
09
10
11
12
13
14
15
20
21
22
24
26
27
28
29
30

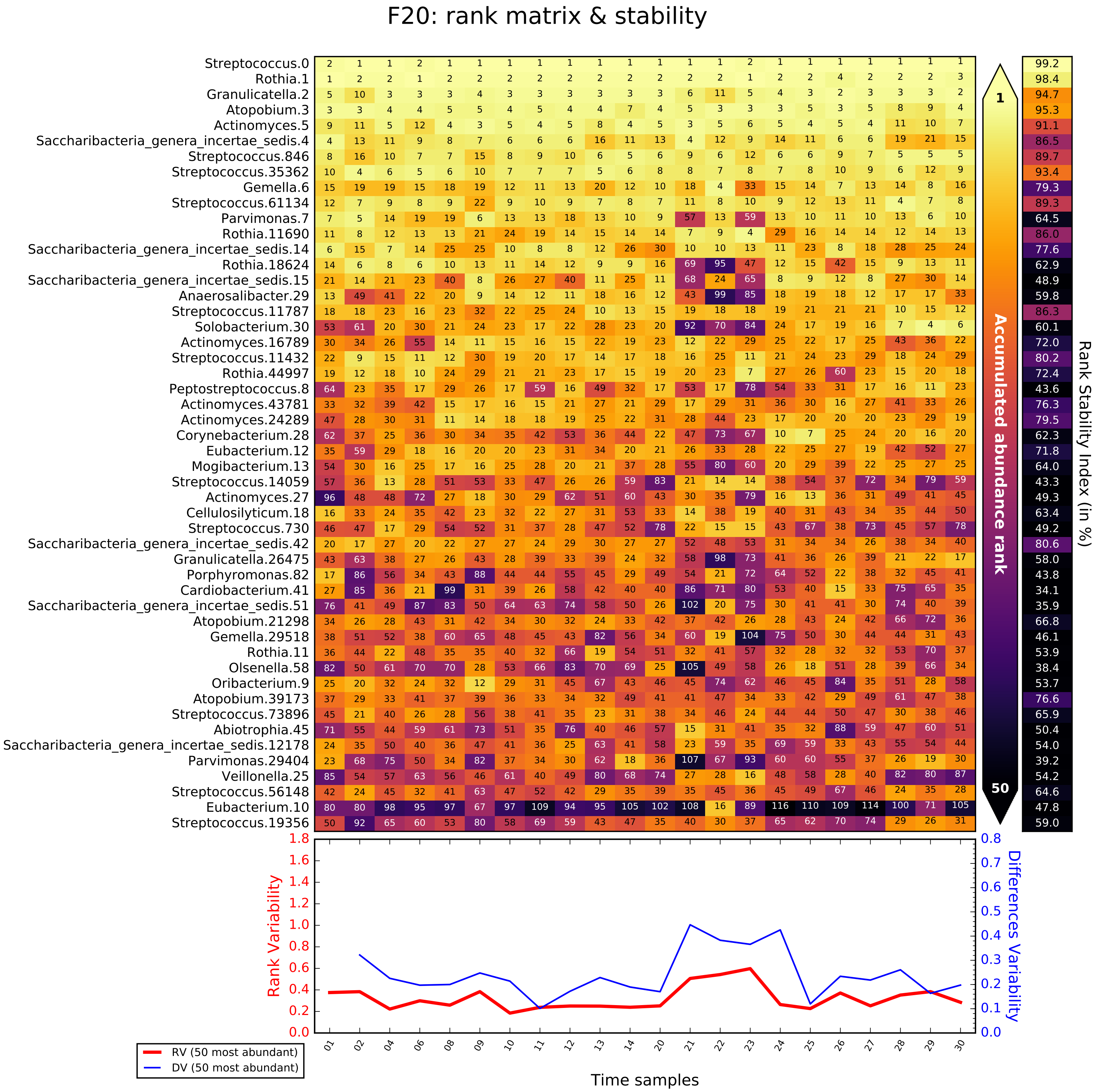
F20: rank matrix & stability
Streptococcus.0
Rothia.1
Granulicatella.2
Atopobium.3
Actinomyces.5
Saccharibacteria_genera_incertae_sedis.4
Streptococcus.846
Streptococcus.35362
Gemella.6
Streptococcus.61134
Parvimonas.7
Rothia.11690
Saccharibacteria_genera_incertae_sedis.14
Rothia.18624
Saccharibacteria_genera_incertae_sedis.15
Anaerosalibacter.29
Streptococcus.11787
Solobacterium.30
Actinomyces.16789
Streptococcus.11432
Rothia.44997
Peptostreptococcus.8
Actinomyces.43781
Actinomyces.24289
Corynebacterium.28
Eubacterium.12
Mogibacterium.13
Streptococcus.14059
Actinomyces.27
Cellulosilyticum.18
Streptococcus.730
Saccharibacteria_genera_incertae_sedis.42
Granulicatella.26475
Porphyromonas.82
Cardiobacterium.41
Saccharibacteria_genera_incertae_sedis.51
Atopobium.21298
Gemella.29518
Rothia.11
Olsenella.58
Oribacterium.9
Atopobium.39173
Streptococcus.73896
Abiotrophia.45
Saccharibacteria_genera_incertae_sedis.12178
Parvimonas.29404
Veillonella.25
Streptococcus.56148
Eubacterium.10
Streptococcus.19356
Accumulated abundance rank
1
50
Rank Stability Index (in %)
99.2
98.4
94.7
95.3
91.1
86.5
89.7
93.4
79.3
89.3
64.5
86.0
77.6
62.9
48.9
59.8
86.3
60.1
72.0
80.2
72.4
43.6
76.3
79.5
62.3
71.8
64.0
43.3
49.3
63.4
49.2
80.6
58.0
43.8
34.1
35.9
66.8
46.1
53.9
38.4
53.7
76.6
65.9
50.4
54.0
39.2
54.2
64.6
47.8
59.0
Rank Variability
Differences Variability
RV (50 most abundant)
DV (50 most abundant)
Time samples
01 02 04 06 08 09 10 11 12 13 14 20 21 22 23 24 25 26 27 28 29 30

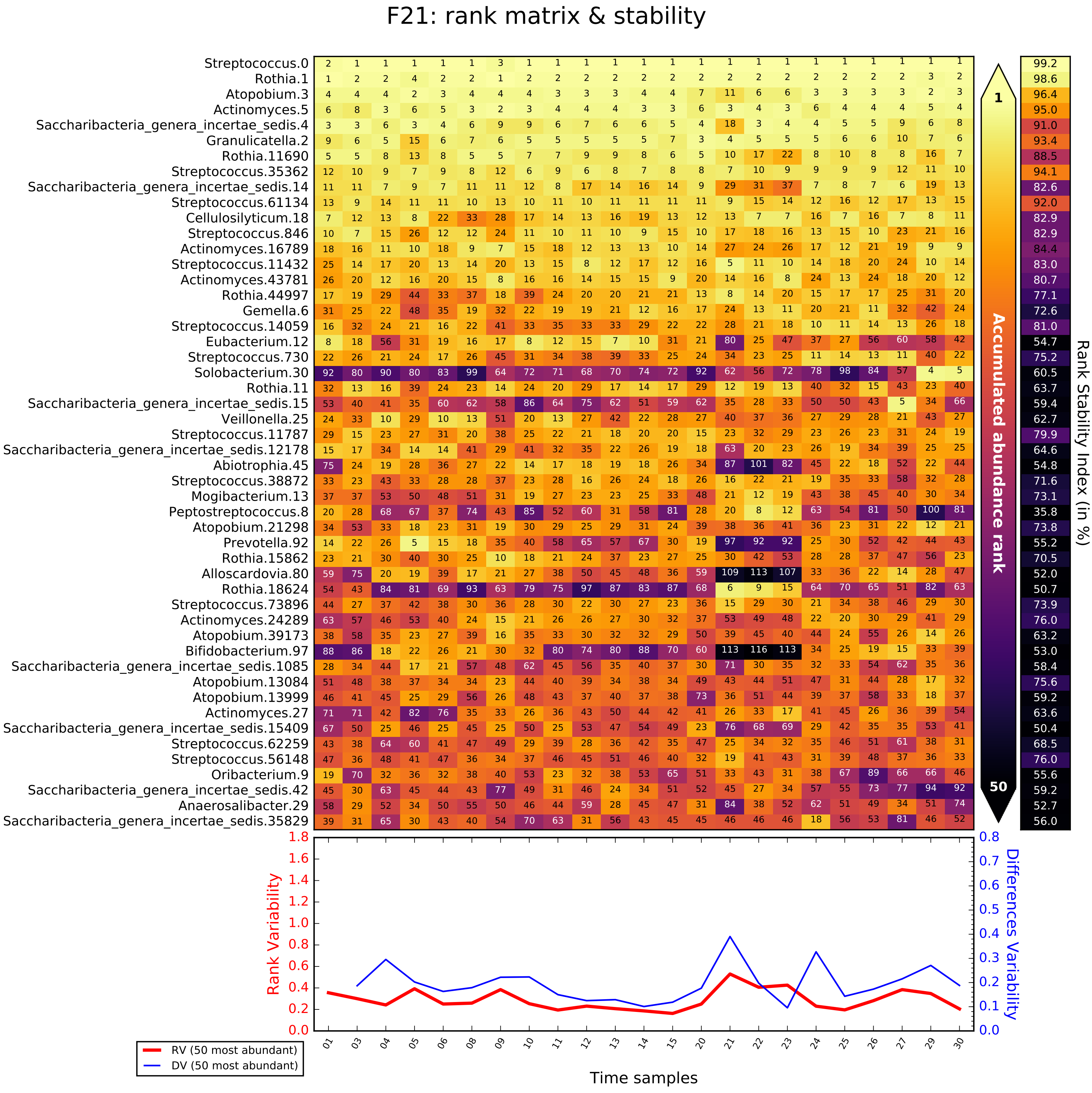
F21: rank matrix & stability
Streptococcus.0
Rothia.1
Atopobium.3
Actinomyces.5
Saccharibacteria_genera_incertae_sedis.4
Granulicatella.2
Rothia.11690
Streptococcus.35362
Saccharibacteria_genera_incertae_sedis.14
Streptococcus.61134
Cellulosilyticum.18
Streptococcus.846
Actinomyces.16789
Streptococcus.11432
Actinomyces.43781
Rothia.44997
Gemella.6
Streptococcus.14059
Eubacterium.12
Streptococcus.730
Solobacterium.30
Rothia.11
Saccharibacteria_genera_incertae_sedis.15
Veillonella.25
Streptococcus.11787
Saccharibacteria_genera_incertae_sedis.12178
Abiotrophia.45
Streptococcus.38872
Mogibacterium.13
Peptostreptococcus.8
Atopobium.21298
Prevotella.92
Rothia.15862
Alloscardovia.80
Rothia.18624
Streptococcus.73896
Actinomyces.24289
Atopobium.39173
Bifidobacterium.97
Saccharibacteria_genera_incertae_sedis.1085
Atopobium.13084
Atopobium.13999
Actinomyces.27
Saccharibacteria_genera_incertae_sedis.15409
Streptococcus.62259
Streptococcus.56148
Oribacterium.9
Saccharibacteria_genera_incertae_sedis.42
Anaerosalibacter.29
Saccharibacteria_genera_incertae_sedis.35829
Accumulated abundance rank
1
50
Rank Stability Index (in %)
Rank Variability
Differences Variability
RV (50 most abundant)
DV (50 most abundant)
Time samples
01 03 04 05 06 08 09 10 11 12 13 14 15 20 21 22 23 24 25 26 27 29 30

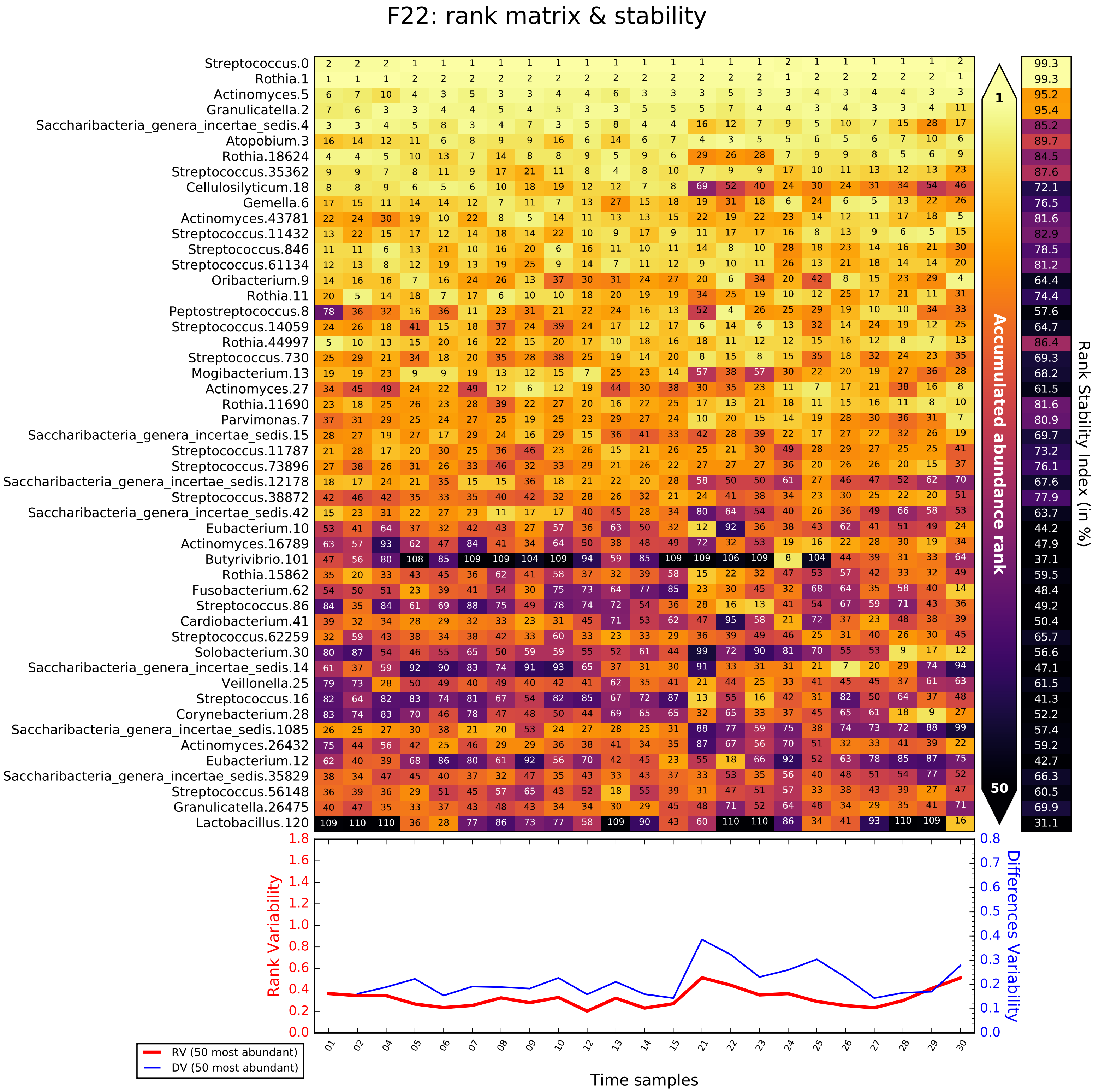
F22: rank matrix & stability
Streptococcus.0
Rothia.1
Actinomyces.5
Granulicatella.2
Saccharibacteria_genera_incertae_sedis.4
Atopobium.3
Rothia.18624
Streptococcus.35362
Cellulosilyticum.18
Gemella.6
Actinomyces.43781
Streptococcus.11432
Streptococcus.846
Streptococcus.61134
Oribacterium.9
Rothia.11
Peptostreptococcus.8
Streptococcus.14059
Rothia.44997
Streptococcus.730
Mogibacterium.13
Actinomyces.27
Rothia.11690
Parvimonas.7
Saccharibacteria_genera_incertae_sedis.15
Streptococcus.11787
Streptococcus.73896
Saccharibacteria_genera_incertae_sedis.12178
Streptococcus.38872
Saccharibacteria_genera_incertae_sedis.42
Eubacterium.10
Actinomyces.16789
Butyrivibrio.101
Rothia.15862
Fusobacterium.62
Streptococcus.86
Cardiobacterium.41
Streptococcus.62259
Solobacterium.30
Saccharibacteria_genera_incertae_sedis.14
Veillonella.25
Streptococcus.16
Corynebacterium.28
Saccharibacteria_genera_incertae_sedis.1085
Actinomyces.26432
Eubacterium.12
Saccharibacteria_genera_incertae_sedis.35829
Streptococcus.56148
Granulicatella.26475
Lactobacillus.120
Accumulated abundance rank
1
50
Rank Stability Index (in %)
Rank Variability
Differences Variability
RV (50 most abundant)
DV (50 most abundant)
Time samples
01 02 04 05 06 07 08 09 10 12 13 14 15 21 22 23 24 25 26 27 28 29 30

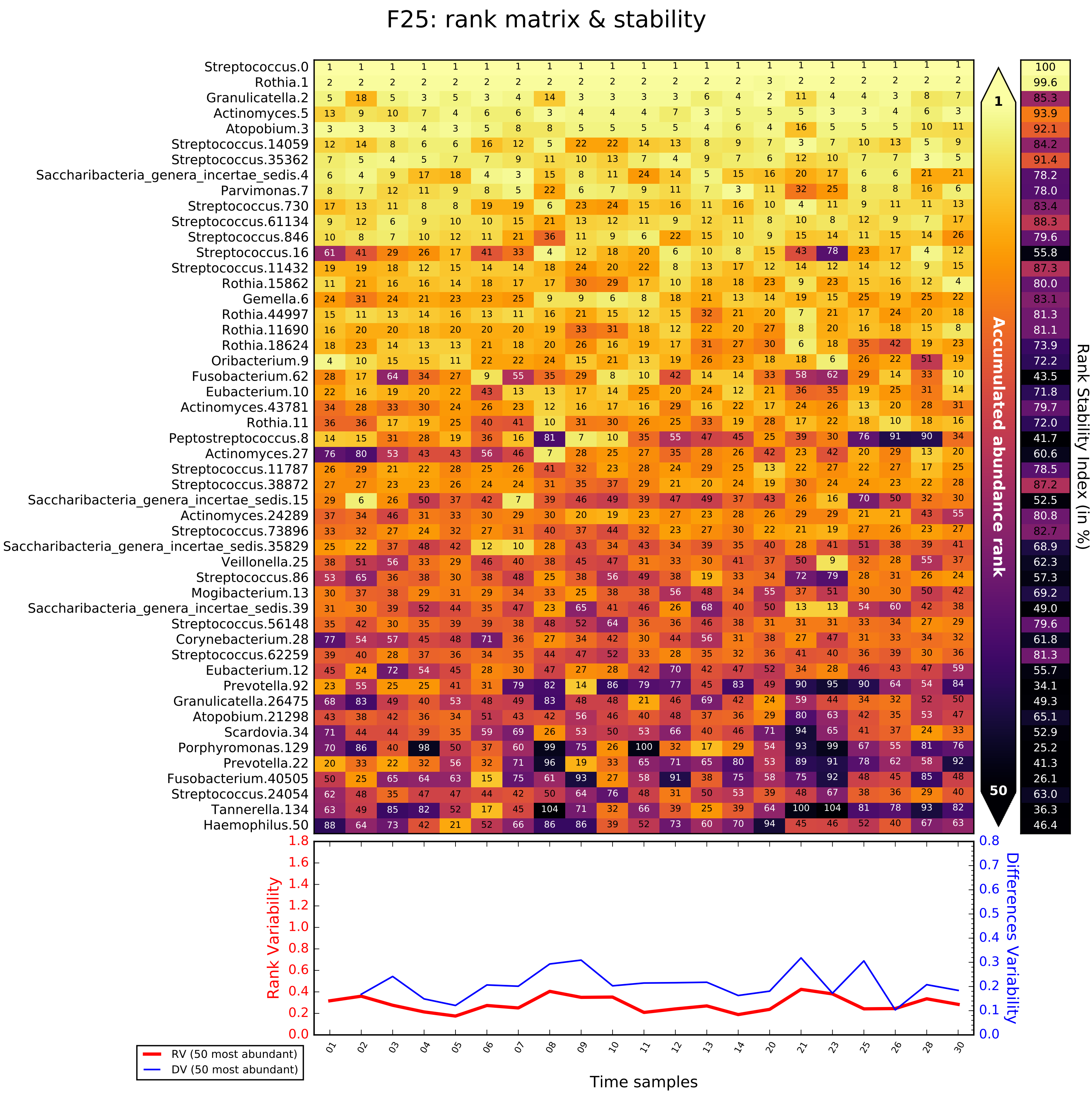
F25: rank matrix & stability
Streptococcus.0
Rothia.1
Granulicatella.2
Actinomyces.5
Atopobium.3
Streptococcus.14059
Streptococcus.35362
Saccharibacteria_genera_incertae_sedis.4
Parvimonas.7
Streptococcus.730
Streptococcus.61134
Streptococcus.846
Streptococcus.16
Streptococcus.11432
Rothia.15862
Gemella.6
Rothia.44997
Rothia.11690
Rothia.18624
Oribacterium.9
Fusobacterium.62
Eubacterium.10
Actinomyces.43781
Rothia.11
Peptostreptococcus.8
Actinomyces.27
Streptococcus.11787
Streptococcus.38872
Saccharibacteria_genera_incertae_sedis.15
Actinomyces.24289
Streptococcus.73896
Saccharibacteria_genera_incertae_sedis.35829
Veillonella.25
Streptococcus.86
Mogibacterium.13
Saccharibacteria_genera_incertae_sedis.39
Streptococcus.56148
Corynebacterium.28
Streptococcus.62259
Eubacterium.12
Prevotella.92
Granulicatella.26475
Atopobium.21298
Scardovia.34
Porphyromonas.129
Prevotella.22
Fusobacterium.40505
Streptococcus.24054
Tannerella.134
Haemophilus.50
Accumulated abundance rank
Rank Stability Index (in %)
Rank Variability
Differences Variability
RV (50 most abundant)
DV (50 most abundant)
Time samples

M01: rank matrix & stability

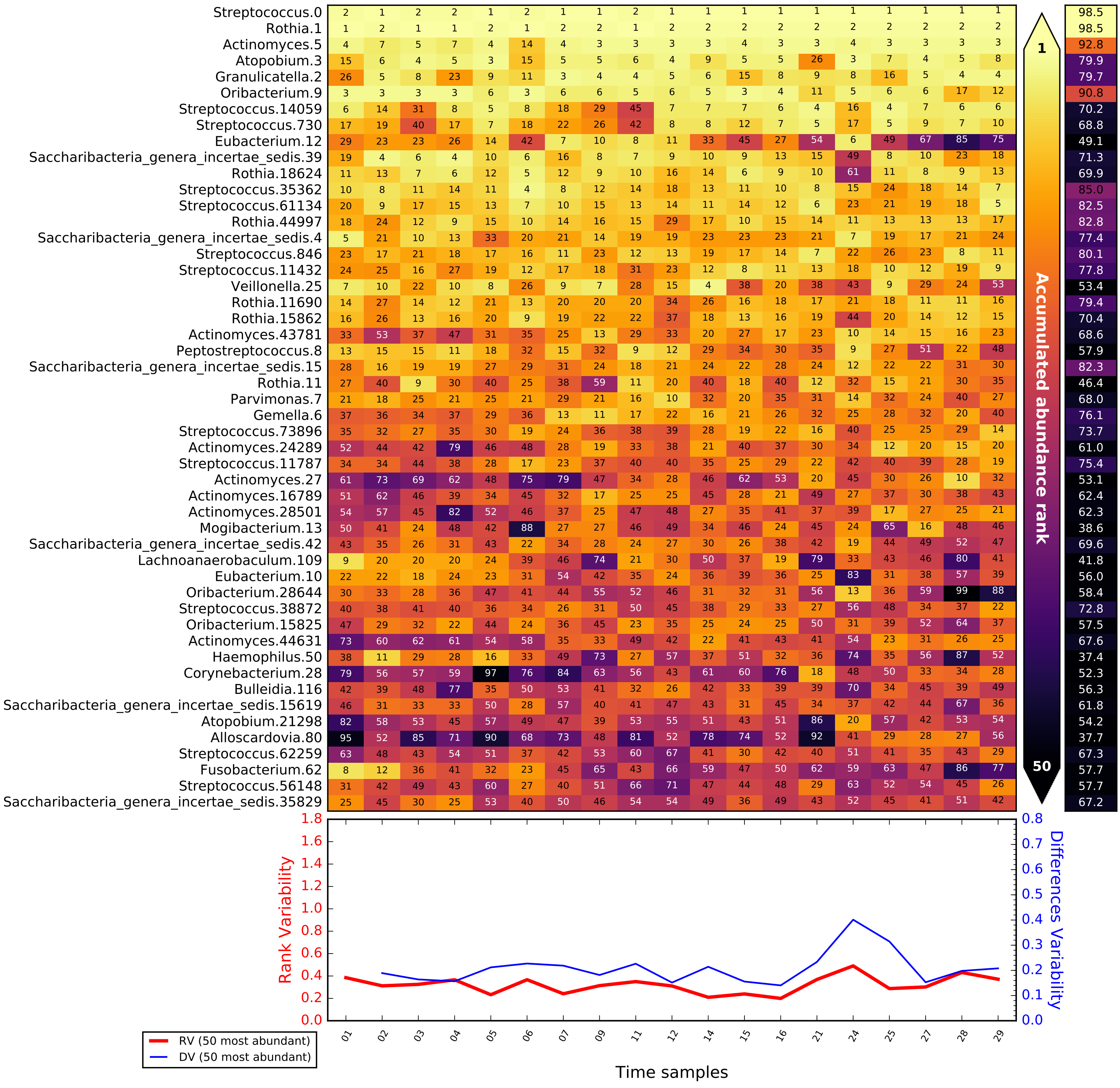

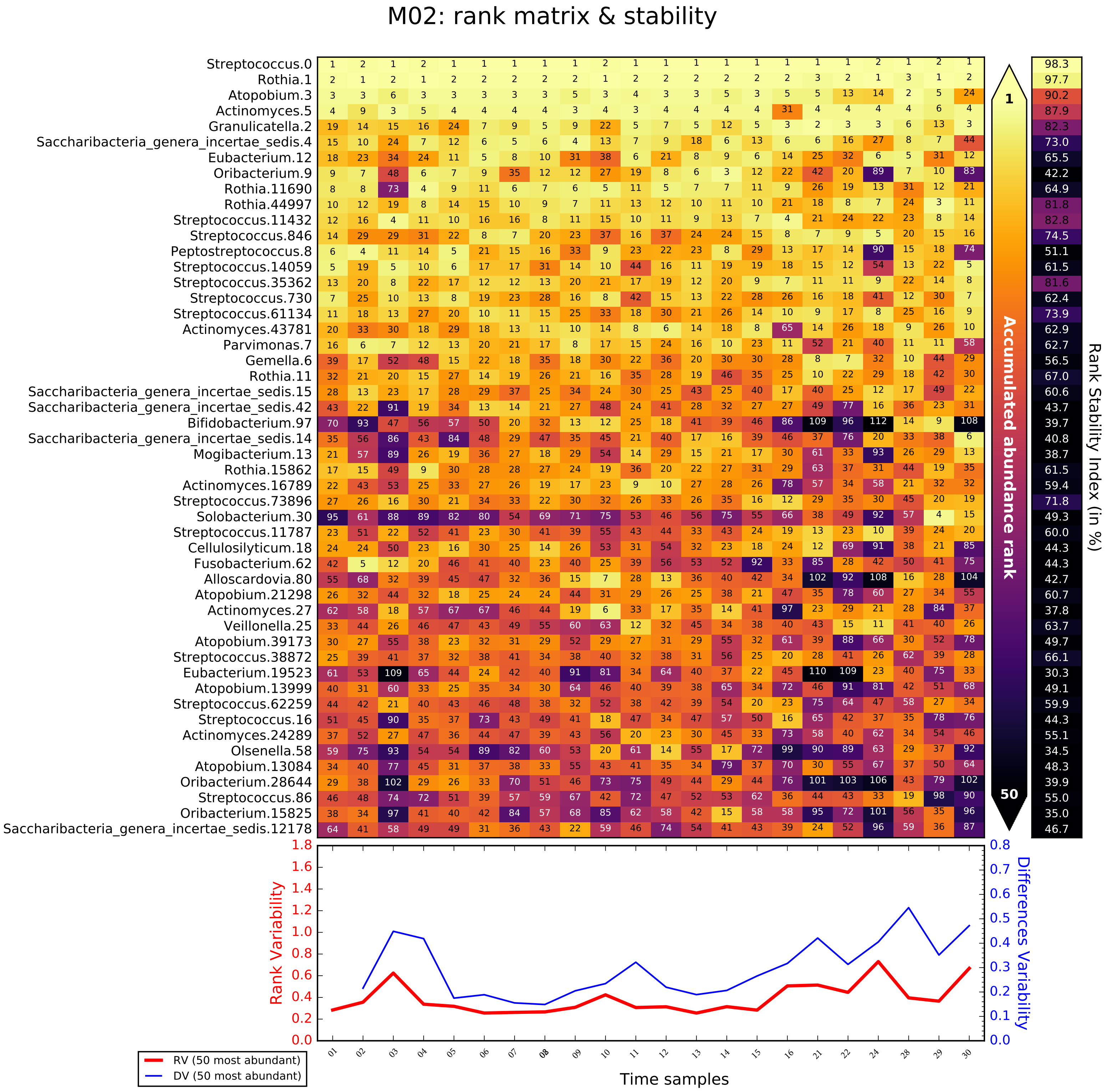
M02: rank matrix & stability
Streptococcus.0
Rothia.1
Atopobium.3
Actinomyces.5
Granulicatella.2
Saccharibacteria_genera_incertae_sedis.4
Eubacterium.12
Oribacterium.9
Rothia.11690
Rothia.44997
Streptococcus.11432
Streptococcus.846
Peptostreptococcus.8
Streptococcus.14059
Streptococcus.35362
Streptococcus.730
Streptococcus.61134
Actinomyces.43781
Parvimonas.7
Gemella.6
Rothia.11
Saccharibacteria_genera_incertae_sedis.15
Saccharibacteria_genera_incertae_sedis.42
Bifidobacterium.97
Saccharibacteria_genera_incertae_sedis.14
Mogibacterium.13
Rothia.15862
Actinomyces.16789
Streptococcus.73896
Solobacterium.30
Streptococcus.11787
Cellulosilyticum.18
Fusobacterium.62
Alloscardovia.80
Atopobium.21298
Actinomyces.27
Veillonella.25
Atopobium.39173
Streptococcus.38872
Eubacterium.19523
Atopobium.13999
Streptococcus.62259
Streptococcus.16
Actinomyces.24289
Olsenella.58
Atopobium.13084
Oribacterium.28644
Streptococcus.86
Oribacterium.15825
Saccharibacteria_genera_incertae_sedis.12178
Accumulated abundance rank
1
50
Rank Stability Index (in %)
98.3
97.7
90.2
87.9
82.3
73.0
65.5
42.2
64.9
81.8
82.8
74.5
51.1
61.5
81.6
62.4
73.9
62.9
62.7
56.5
67.0
60.6
43.7
39.7
40.8
38.7
61.5
59.4
71.8
49.3
60.0
44.3
44.3
42.7
60.7
37.8
63.7
49.7
66.1
30.3
49.1
59.9
44.3
55.1
34.5
48.3
39.9
55.0
35.0
46.7
Rank Variability
Differences Variability
RV (50 most abundant)
DV (50 most abundant)
Time samples
01
02
03
04
05
06
07
08
09
10
11
12
13
14
15
16
21
22
24
28
29
30

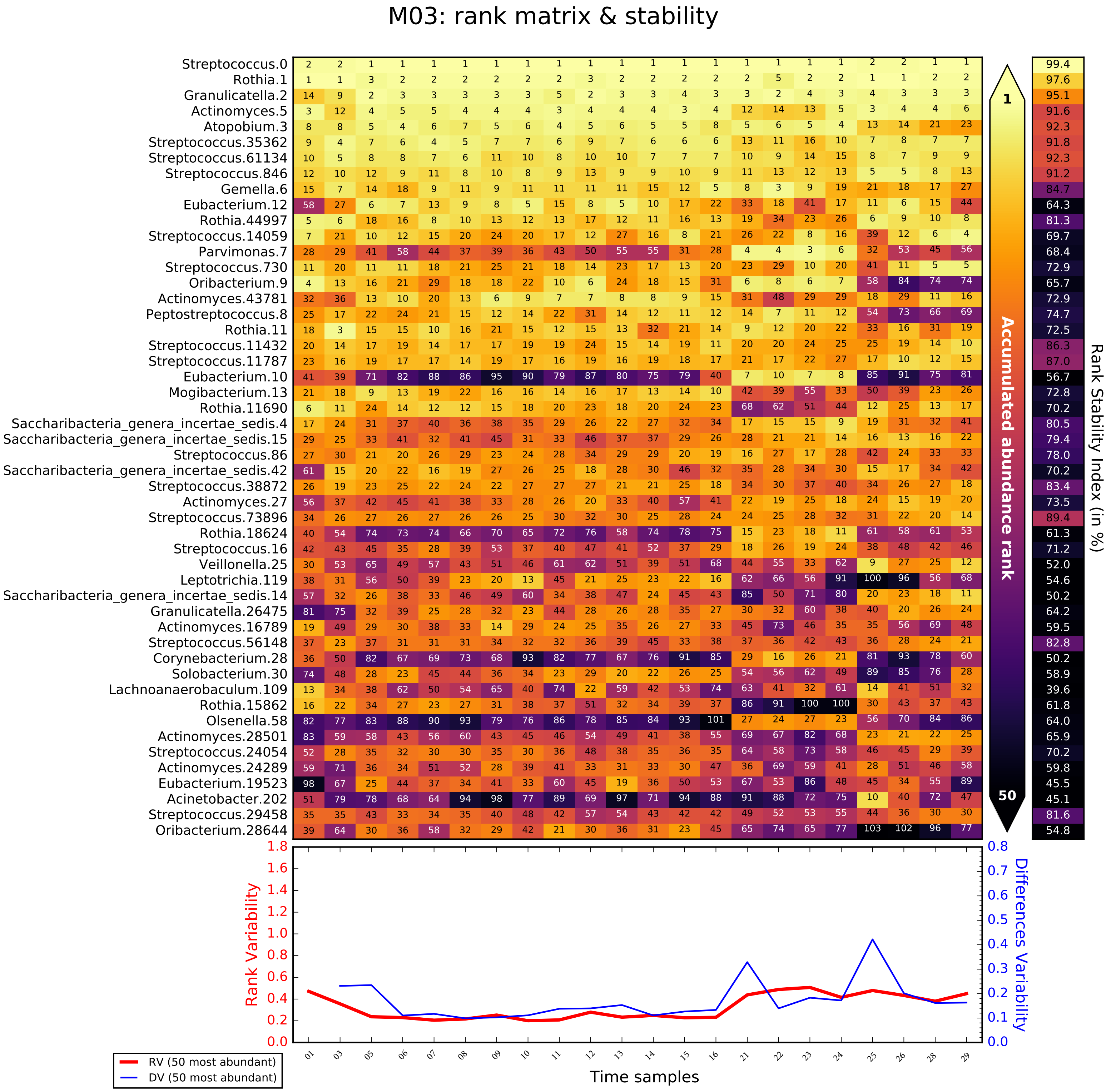
M03: rank matrix & stability
Streptococcus.0
Rothia.1
Granulicatella.2
Actinomyces.5
Atopobium.3
Streptococcus.35362
Streptococcus.61134
Streptococcus.846
Gemella.6
Eubacterium.12
Rothia.44997
Streptococcus.14059
Parvimonas.7
Streptococcus.730
Oribacterium.9
Actinomyces.43781
Peptostreptococcus.8
Rothia.11
Streptococcus.11432
Streptococcus.11787
Eubacterium.10
Mogibacterium.13
Rothia.11690
Saccharibacteria_genera_incertae_sedis.4
Saccharibacteria_genera_incertae_sedis.15
Streptococcus.86
Saccharibacteria_genera_incertae_sedis.42
Streptococcus.38872
Actinomyces.27
Streptococcus.73896
Rothia.18624
Streptococcus.16
Veillonella.25
Leptotrichia.119
Saccharibacteria_genera_incertae_sedis.14
Granulicatella.26475
Actinomyces.16789
Streptococcus.56148
Corynebacterium.28
Solobacterium.30
Lachnoanaerobaculum.109
Rothia.15862
Olsenella.58
Actinomyces.28501
Streptococcus.24054
Actinomyces.24289
Eubacterium.19523
Acinetobacter.202
Streptococcus.29458
Oribacterium.28644
Accumulated abundance rank
Rank Stability Index (in %)
Rank Variability
Differences Variability
RV (50 most abundant)
DV (50 most abundant)
Time samples

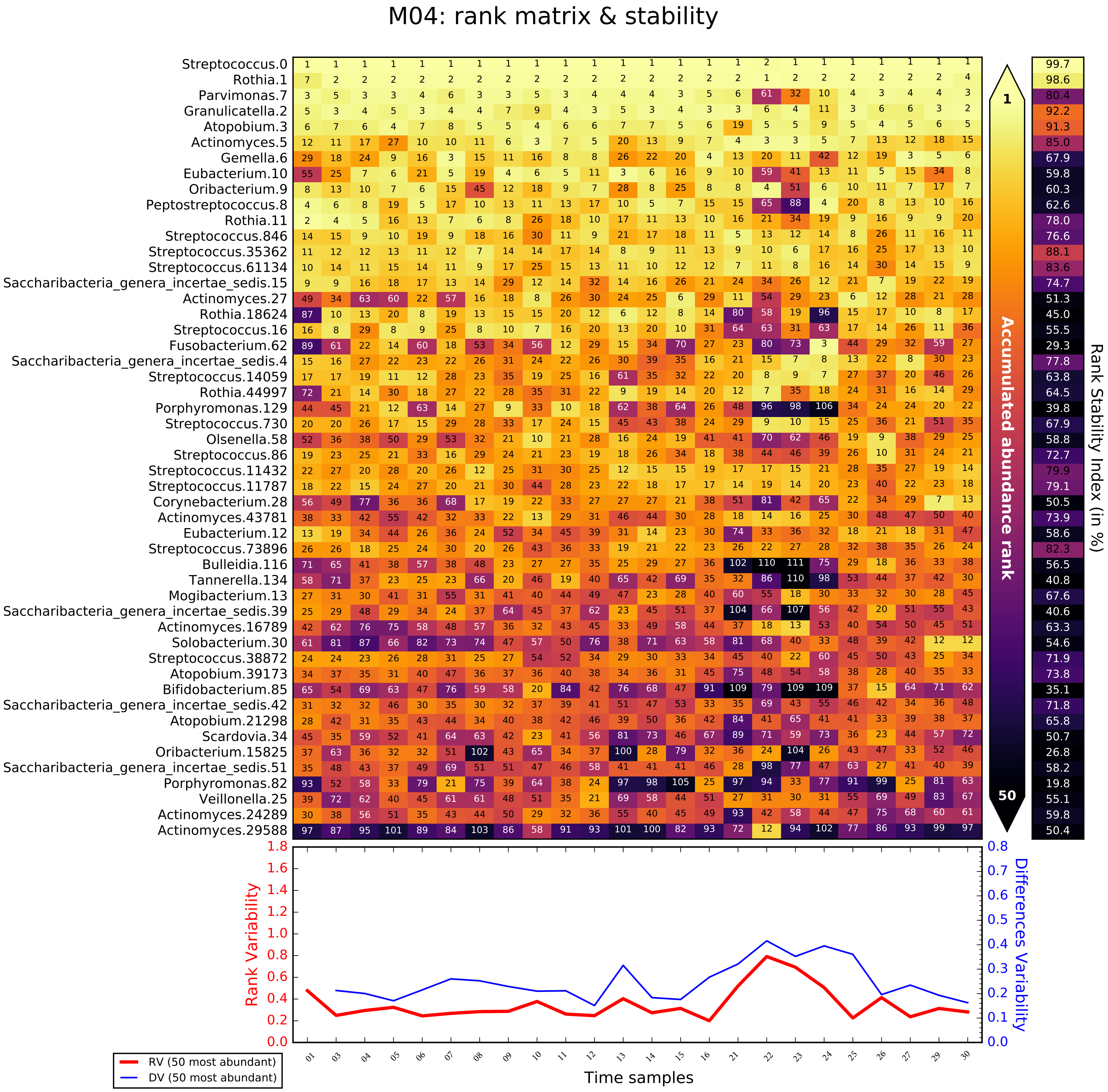
M04: rank matrix & stability
Streptococcus.0
Rothia.1
Parvimonas.7
Granulicatella.2
Atopobium.3
Actinomyces.5
Gemella.6
Eubacterium.10
Oribacterium.9
Peptostreptococcus.8
Rothia.11
Streptococcus.846
Streptococcus.35362
Streptococcus.61134
Saccharibacteria_genera_incertae_sedis.15
Actinomyces.27
Rothia.18624
Streptococcus.16
Fusobacterium.62
Saccharibacteria_genera_incertae_sedis.4
Streptococcus.14059
Rothia.44997
Porphyromonas.129
Streptococcus.730
Olsenella.58
Streptococcus.86
Streptococcus.11432
Streptococcus.11787
Corynebacterium.28
Actinomyces.43781
Eubacterium.12
Streptococcus.73896
Bulleidia.116
Tannerella.134
Mogibacterium.13
Saccharibacteria_genera_incertae_sedis.39
Actinomyces.16789
Solobacterium.30
Streptococcus.38872
Atopobium.39173
Bifidobacterium.85
Saccharibacteria_genera_incertae_sedis.42
Atopobium.21298
Scardovia.34
Oribacterium.15825
Saccharibacteria_genera_incertae_sedis.51
Porphyromonas.82
Veillonella.25
Actinomyces.24289
Actinomyces.29588
Accumulated abundance rank
1
50
Rank Stability Index (in %)
99.7
98.6
80.4
92.2
91.3
85.0
67.9
59.8
60.3
62.6
78.0
76.6
88.1
83.6
74.7
51.3
45.0
55.5
29.3
77.8
63.8
64.5
39.8
67.9
58.8
72.7
79.9
79.1
50.5
73.9
58.6
82.3
56.5
40.8
67.6
40.6
63.3
54.6
71.9
73.8
35.1
71.8
65.8
50.7
26.8
58.2
19.8
55.1
59.8
50.4
Rank Variability
Differences Variability
Time samples
01 03 04 05 06 07 08 09 10 11 12 13 14 15 16 21 22 23 24 25 26 27 29 30
RV (50 most abundant)
DV (50 most abundant)

M05: rank matrix & stability

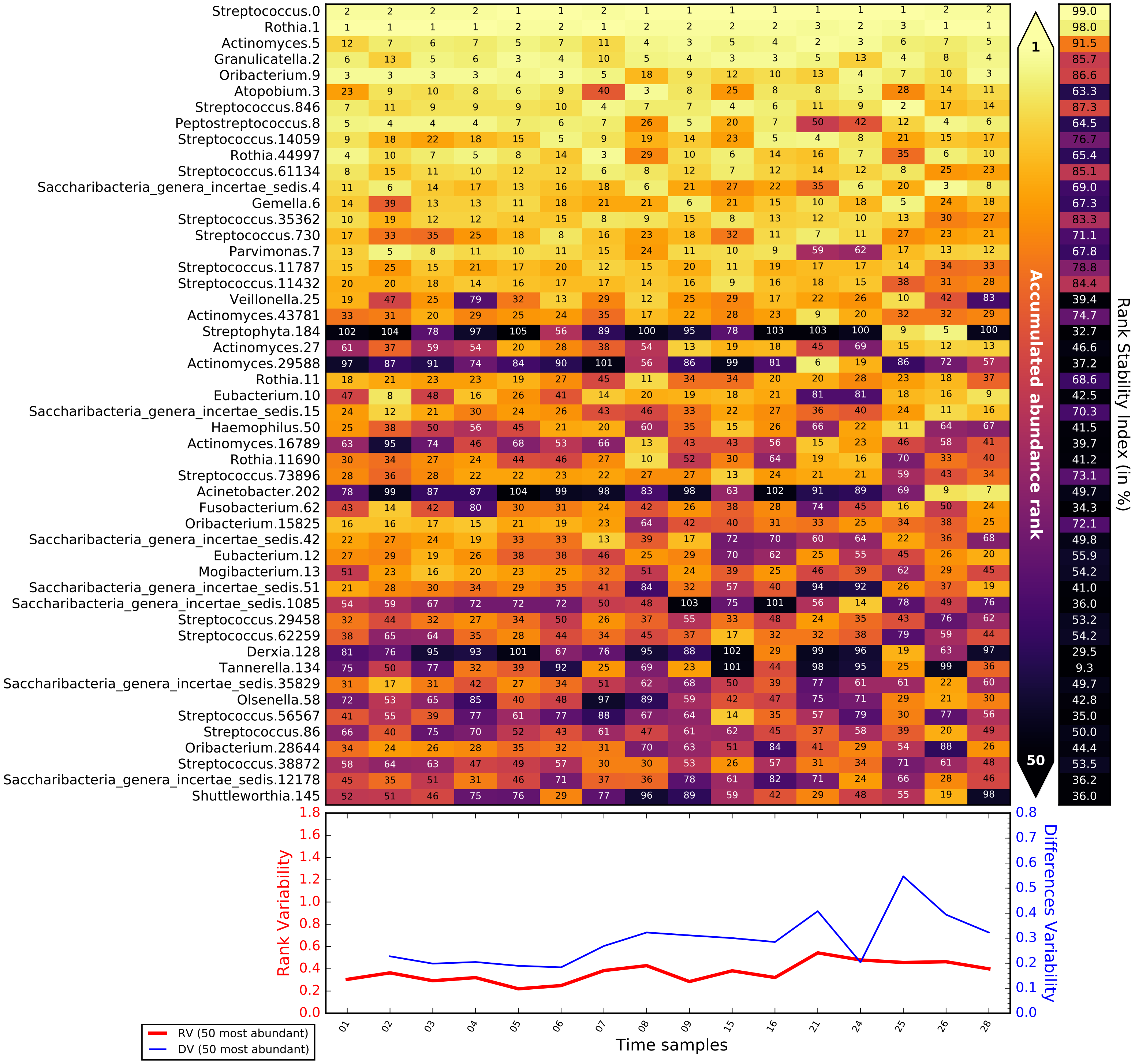

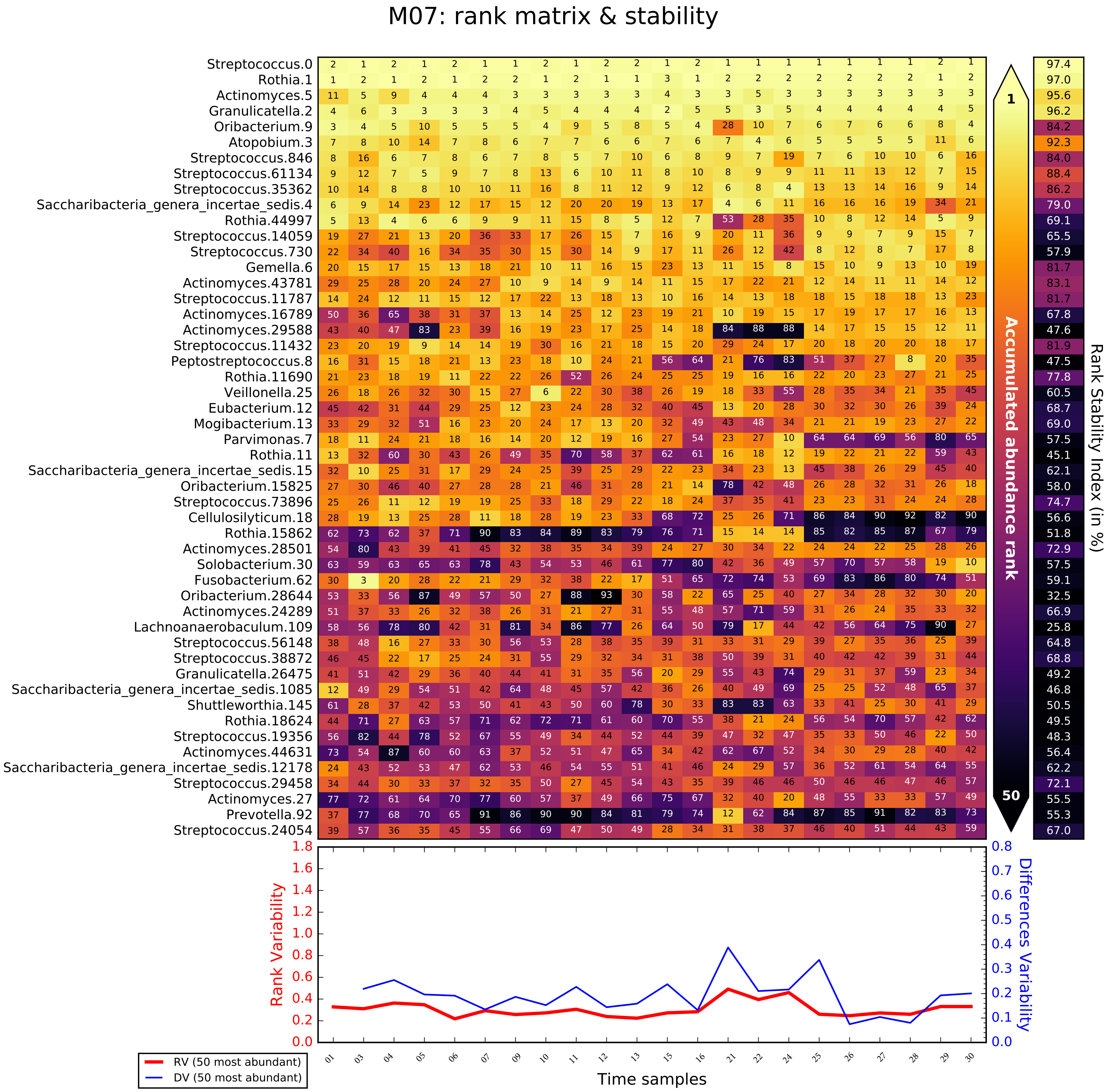
M07: rank matrix & stability
Streptococcus.0
Rothia.1
Actinomyces.5
Granulicatella.2
Oribacterium.9
Atopobium.3
Streptococcus.846
Streptococcus.61134
Streptococcus.35362
Saccharibacteria_genera_incertae_sedis.4
Rothia.44997
Streptococcus.14059
Streptococcus.730
Gemella.6
Actinomyces.43781
Streptococcus.11787
Actinomyces.16789
Actinomyces.29588
Streptococcus.11432
Peptostreptococcus.8
Rothia.11690
Veillonella.25
Eubacterium.12
Mogibacterium.13
Parvimonas.7
Rothia.11
Saccharibacteria_genera_incertae_sedis.15
Oribacterium.15825
Streptococcus.73896
Cellulosilyticum.18
Rothia.15862
Actinomyces.28501
Solobacterium.30
Fusobacterium.62
Oribacterium.28644
Actinomyces.24289
Lachnoanaerobaculum.109
Streptococcus.56148
Streptococcus.38872
Granulicatella.26475
Saccharibacteria_genera_incertae_sedis.1085
Shuttleworthia.145
Rothia.18624
Streptococcus.19356
Actinomyces.44631
Saccharibacteria_genera_incertae_sedis.12178
Streptococcus.29458
Actinomyces.27
Prevotella.92
Streptococcus.24054
Accumulated abundance rank
1
50
Rank Stability Index (in %)
97.4
97.0
95.6
96.2
84.2
92.3
84.0
88.4
86.2
79.0
69.1
65.5
57.9
81.7
83.1
81.7
67.8
47.6
81.9
47.5
77.8
60.5
68.7
69.0
57.5
45.1
62.1
58.0
74.7
56.6
51.8
72.9
57.5
59.1
32.5
66.9
25.8
64.8
68.8
49.2
46.8
50.5
49.5
48.3
56.4
62.2
72.1
55.5
55.3
67.0
Rank Variability
Differences Variability
Time samples
01 03 04 05 06 07 09 10 11 12 13 15 16 21 22 24 25 26 27 28 29 30
RV (50 most abundant)
DV (50 most abundant)

M08: rank matrix & stability

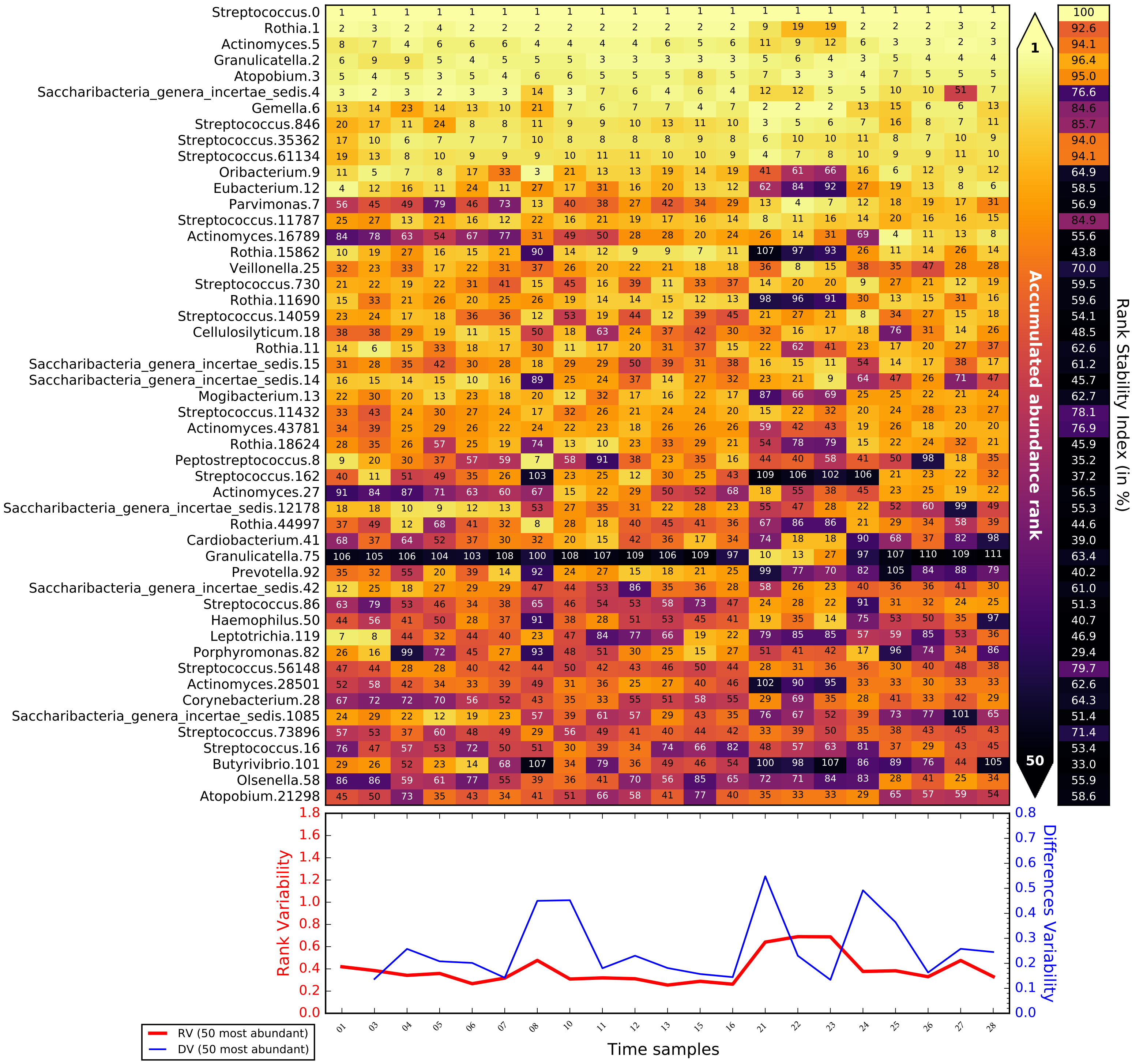

M12: rank matrix & stability

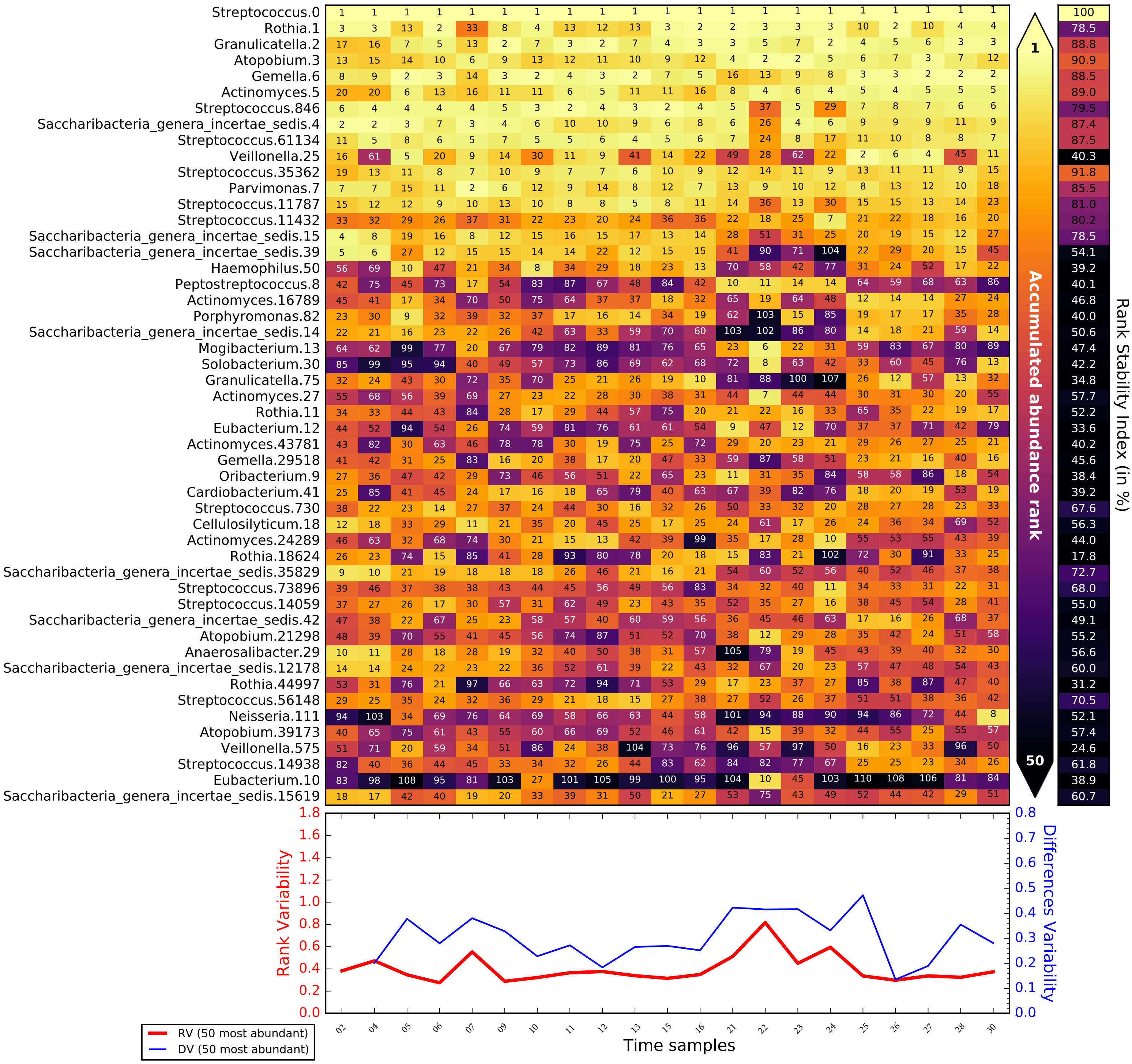

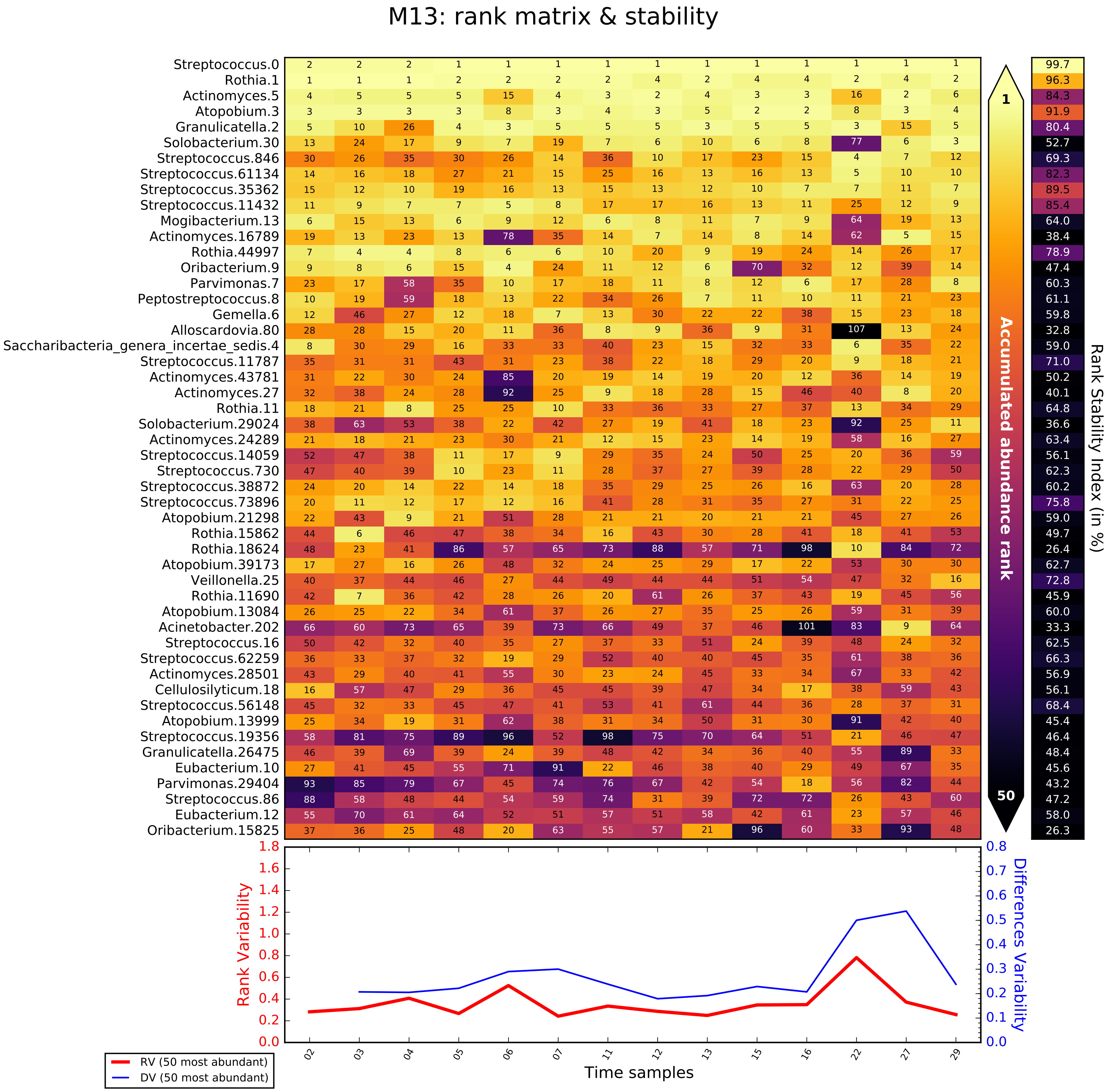
M13: rank matrix & stability
Streptococcus.0 2 2 2 1 1 1 1 1 1 1 1 1 1 1 99.7
Rothia.1 1 1 1 2 2 2 2 4 2 4 4 2 4 2 96.3
Actinomyces.5 4 5 5 5 15 4 3 2 4 3 3 16 2 6 84.3
Atopobium.3 3 3 3 3 8 3 4 3 5 2 2 8 3 4 91.9
Granulicatella.2 5 10 26 4 3 5 5 5 3 5 5 3 15 5 80.4
Solobacterium.30 13 24 17 9 7 19 7 6 10 6 8 77 6 3 52.7
Streptococcus.846 30 26 35 30 26 14 36 10 17 23 15 4 7 12 69.3
Streptococcus.61134 14 16 18 27 21 15 25 16 13 16 13 5 10 10 82.3
Streptococcus.35362 15 12 10 19 16 13 15 13 12 10 7 7 11 7 89.5
Streptococcus.11432 11 9 7 7 5 8 17 17 16 13 11 25 12 9 85.4
Mogibacterium.13 6 15 13 6 9 12 6 8 11 7 9 64 19 13 64.0
Actinomyces.16789 19 13 23 13 78 35 14 7 14 8 14 62 5 15 38.4
Rothia.44997 7 4 4 8 6 6 10 20 9 19 24 14 26 17 78.9
Oribacterium.9 9 8 6 15 4 24 11 12 6 70 32 12 39 14 47.4
Parvimonas.7 23 17 58 35 10 17 18 11 8 12 6 17 28 8 60.3
Peptostreptococcus.8 10 19 59 18 13 22 34 26 7 11 10 11 21 23 61.1
Gemella.6 12 46 27 12 18 7 13 30 22 22 38 15 23 18 59.8
Alloscardovia.80 28 28 15 20 11 36 8 9 36 9 31 107 13 24 32.8
Saccharibacteria_genera_incertae_sedis.4 8 30 29 16 33 33 40 23 15 32 33 6 35 22 59.0
Streptococcus.11787 35 31 31 43 31 23 38 22 18 29 20 9 18 21 71.0
Actinomyces.43781 31 22 30 24 85 20 19 14 19 20 12 36 14 19 50.2
Actinomyces.27 32 38 24 28 92 25 9 18 28 15 46 40 8 20 40.1
Rothia.11 18 21 8 25 25 10 33 36 33 27 37 13 34 29 64.8
Solobacterium.29024 38 63 53 38 22 42 27 19 41 18 23 92 25 11 36.6
Actinomyces.24289 21 18 21 23 30 21 12 15 23 14 19 58 16 27 63.4
Streptococcus.14059 52 47 38 11 17 9 29 35 24 50 25 20 36 59 56.1
Streptococcus.730 47 40 39 10 23 11 28 37 27 39 28 22 29 50 62.3
Streptococcus.38872 24 20 14 22 14 18 35 29 25 26 16 63 20 28 60.2
Streptococcus.73896 20 11 12 17 12 16 41 28 31 35 27 31 22 25 75.8
Atopobium.21298 22 43 9 21 51 28 21 21 20 21 21 45 27 26 59.0
Rothia.15862 44 6 46 47 38 34 16 43 30 28 41 18 41 53 49.7
Rothia.18624 48 23 41 86 57 65 73 88 57 71 98 10 84 72 26.4
Atopobium.39173 17 27 16 26 48 32 24 25 29 17 22 53 30 30 62.7
Veillonella.25 40 37 44 46 27 44 49 44 44 51 54 47 32 16 72.8
Rothia.11690 42 7 36 42 28 26 20 61 26 37 43 19 45 56 45.9
Atopobium.13084 26 25 22 34 61 37 26 27 35 25 26 59 31 39 60.0
Acinetobacter.202 66 60 73 65 39 73 66 49 37 46 101 83 9 64 33.3
Streptococcus.16 50 42 32 40 35 27 37 33 51 24 39 48 24 32 62.5
Streptococcus.62259 36 33 37 32 19 29 52 40 40 45 35 61 38 36 66.3
Actinomyces.28501 43 29 40 41 55 30 23 24 45 33 34 67 33 42 56.9
Cellulosilyticum.18 16 57 47 29 36 45 45 39 47 34 17 38 59 43 56.1
Streptococcus.56148 45 32 33 45 47 41 53 41 61 44 36 28 37 31 68.4
Atopobium.13999 25 34 19 31 62 38 31 34 50 31 30 91 42 40 45.4
Streptococcus.19356 58 81 75 89 96 52 98 75 70 64 51 21 46 47 46.4
Granulicatella.26475 46 39 69 39 24 39 48 42 34 36 40 55 89 33 48.4
Eubacterium.10 27 41 45 55 71 91 22 46 38 40 29 49 67 35 45.6
Parvimonas.29404 93 85 79 67 45 74 76 67 42 54 18 56 82 44 43.2
Streptococcus.86 88 58 48 44 54 59 74 31 39 72 72 26 43 60 47.2
Eubacterium.12 55 70 61 64 52 51 57 51 58 42 61 23 57 46 58.0
Oribacterium.15825 37 36 25 48 20 63 55 57 21 96 60 33 93 48 26.3
1
Accumulated abundance rank
50
Rank Stability Index (in %)
Rank Variability
1.8 1.6 1.4 1.2 1.0 0.8 0.6 0.4 0.2 0.0
Differences Variability
0.8 0.7 0.6 0.5 0.4 0.3 0.2 0.1 0.0
02 03 04 05 06 07 11 12 13 15 16 22 27 29
Time samples
RV (50 most abundant)
DV (50 most abundant)

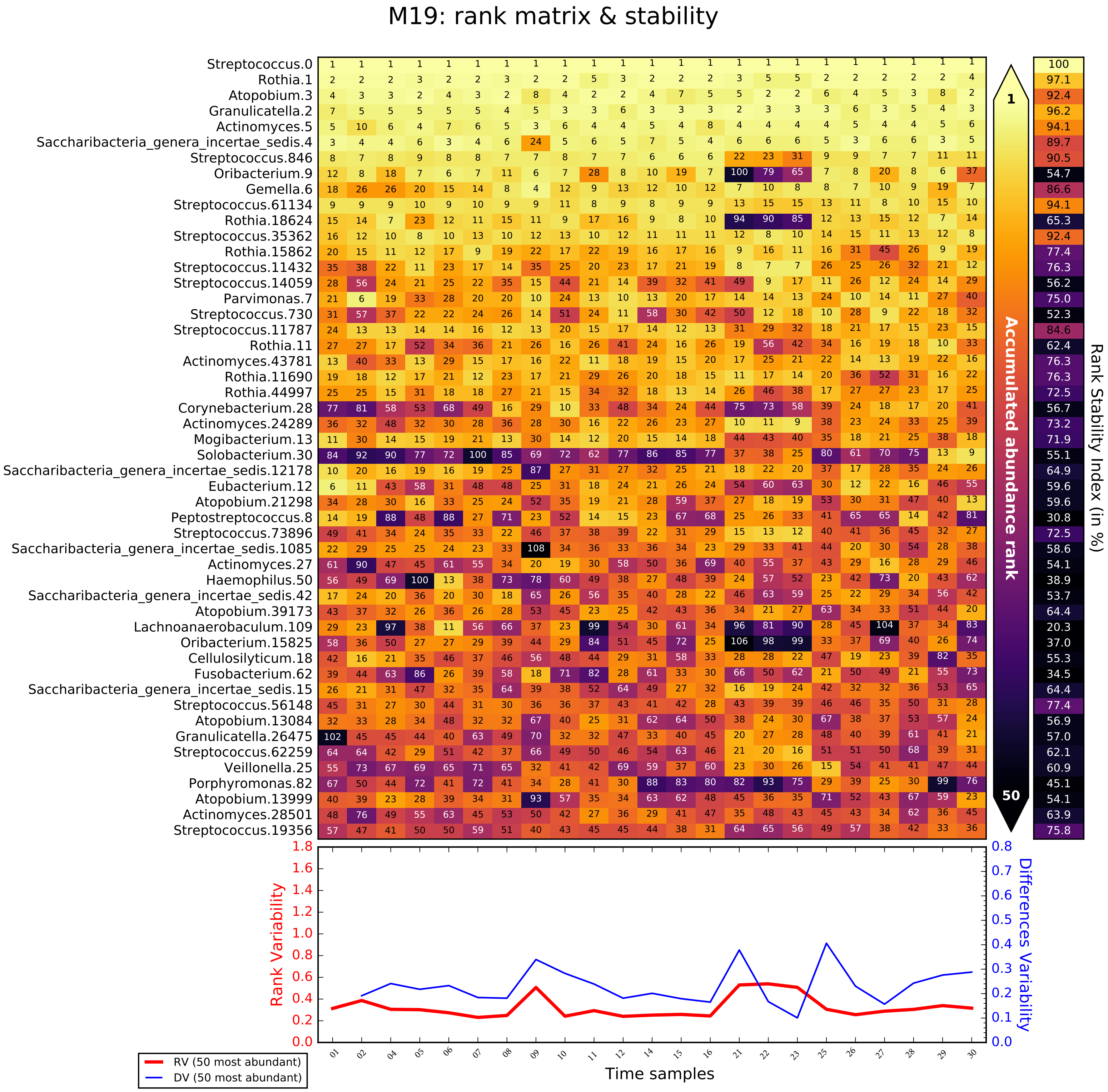
M19: rank matrix & stability
Streptococcus.0
Rothia.1
Atopobium.3
Granulicatella.2
Actinomyces.5
Saccharibacteria_genera_incertae_sedis.4
Streptococcus.846
Oribacterium.9
Gemella.6
Streptococcus.61134
Rothia.18624
Streptococcus.35362
Rothia.15862
Streptococcus.11432
Streptococcus.14059
Parvimonas.7
Streptococcus.730
Streptococcus.11787
Rothia.11
Actinomyces.43781
Rothia.11690
Rothia.44997
Corynebacterium.28
Actinomyces.24289
Mogibacterium.13
Solobacterium.30
Saccharibacteria_genera_incertae_sedis.12178
Eubacterium.12
Atopobium.21298
Peptostreptococcus.8
Streptococcus.73896
Saccharibacteria_genera_incertae_sedis.1085
Actinomyces.27
Haemophilus.50
Saccharibacteria_genera_incertae_sedis.42
Atopobium.39173
Lachnoanaerobaculum.109
Oribacterium.15825
Cellulosilyticum.18
Fusobacterium.62
Saccharibacteria_genera_incertae_sedis.15
Streptococcus.56148
Atopobium.13084
Granulicatella.26475
Streptococcus.62259
Veillonella.25
Porphyromonas.82
Atopobium.13999
Actinomyces.28501
Streptococcus.19356
Accumulated abundance rank
1
50
Rank Stability Index (in %)
100
97.1
92.4
96.2
94.1
89.7
90.5
54.7
86.6
94.1
65.3
92.4
77.4
76.3
56.2
75.0
52.3
84.6
62.4
76.3
76.3
72.5
56.7
73.2
71.9
55.1
64.9
59.6
59.6
30.8
72.5
58.6
54.1
38.9
53.7
64.4
20.3
37.0
55.3
34.5
64.4
77.4
56.9
57.0
62.1
60.9
45.1
54.1
63.9
75.8
Rank Variability
Differences Variability
RV (50 most abundant)
DV (50 most abundant)
Time samples
01
02
04
05
06
07
08
09
10
11
12
14
15
16
21
22
23
25
26
27
28
29
30

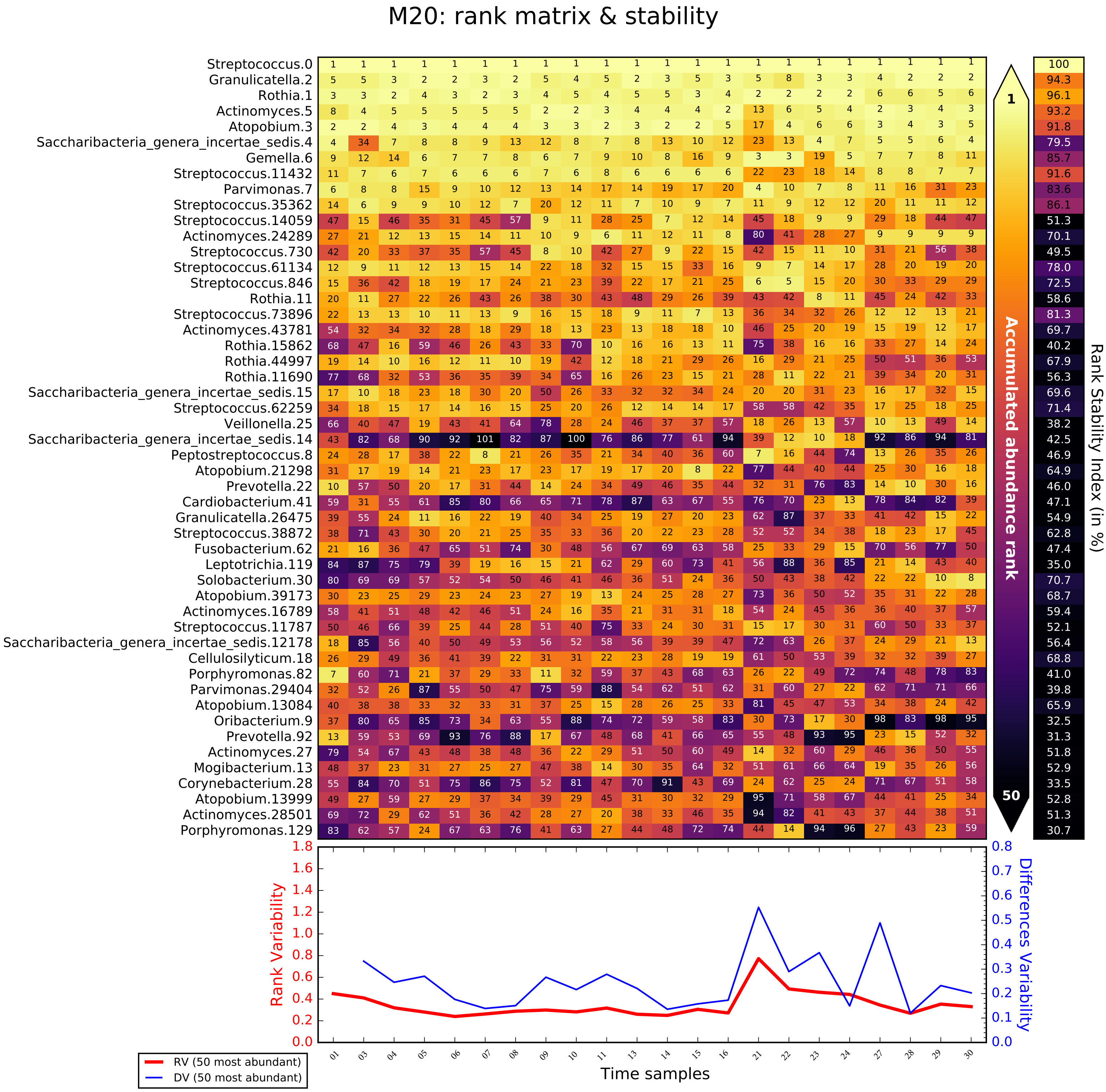

M20: rank matrix & stability
Streptococcus.0
Granulicatella.2
Rothia.1
Actinomyces.5
Atopobium.3
Saccharibacteria_genera_incertae_sedis.4
Gemella.6
Streptococcus.11432
Parvimonas.7
Streptococcus.35362
Streptococcus.14059
Actinomyces.24289
Streptococcus.730
Streptococcus.61134
Streptococcus.846
Rothia.11
Streptococcus.73896
Actinomyces.43781
Rothia.15862
Rothia.44997
Rothia.11690
Saccharibacteria_genera_incertae_sedis.15
Streptococcus.62259
Veillonella.25
Saccharibacteria_genera_incertae_sedis.14
Peptostreptococcus.8
Atopobium.21298
Prevotella.22
Cardiobacterium.41
Granulicatella.26475
Streptococcus.38872
Fusobacterium.62
Leptotrichia.119
Solobacterium.30
Atopobium.39173
Actinomyces.16789
Streptococcus.11787
Saccharibacteria_genera_incertae_sedis.12178
Cellulosilyticum.18
Porphyromonas.82
Parvimonas.29404
Atopobium.13084
Oribacterium.9
Prevotella.92
Actinomyces.27
Mogibacterium.13
Corynebacterium.28
Atopobium.13999
Actinomyces.28501
Porphyromonas.129
Accumulated abundance rank
1
50
Rank Stability Index (in %)
100
94.3
96.1
93.2
91.8
79.5
85.7
91.6
83.6
86.1
51.3
70.1
49.5
78.0
72.5
58.6
81.3
69.7
40.2
67.9
56.3
69.6
71.4
38.2
42.5
46.9
64.9
46.0
47.1
54.9
62.8
47.4
35.0
70.7
68.7
59.4
52.1
56.4
68.8
41.0
39.8
65.9
32.5
31.3
51.8
52.9
33.5
52.8
51.3
30.7
Rank Variability
Differences Variability
Time samples
01 03 04 05 06 07 08 09 10 11 13 14 15 16 21 22 23 24 27 28 29 30
RV (50 most abundant)
DV (50 most abundant)

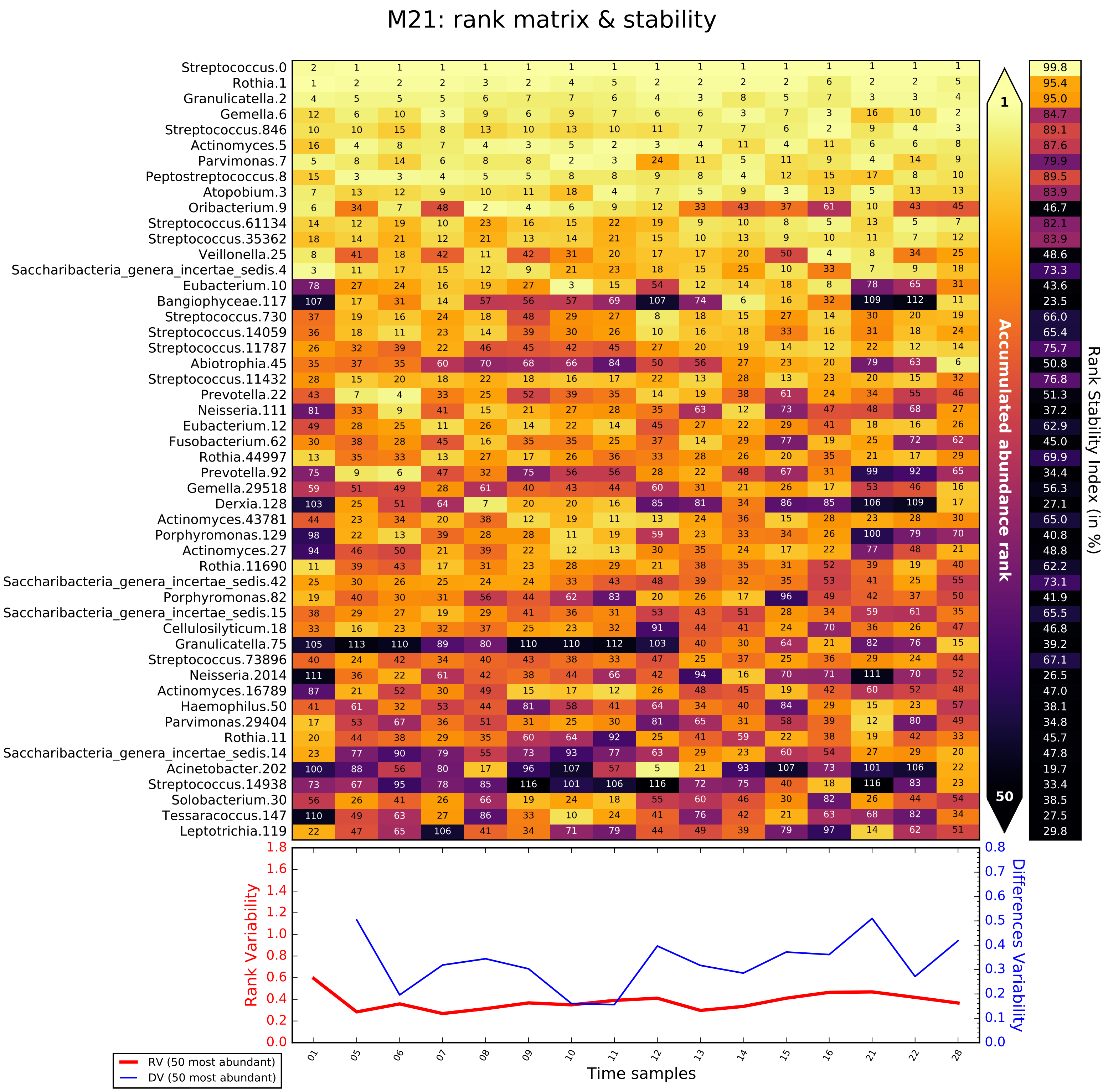
M21: rank matrix & stability
Streptococcus.0
Rothia.1
Granulicatella.2
Gemella.6
Streptococcus.846
Actinomyces.5
Parvimonas.7
Peptostreptococcus.8
Atopobium.3
Oribacterium.9
Streptococcus.61134
Streptococcus.35362
Veillonella.25
Saccharibacteria_genera_incertae_sedis.4
Eubacterium.10
Bangiophyceae.117
Streptococcus.730
Streptococcus.14059
Streptococcus.11787
Abiotrophia.45
Streptococcus.11432
Prevotella.22
Neisseria.111
Eubacterium.12
Fusobacterium.62
Rothia.44997
Prevotella.92
Gemella.29518
Derxia.128
Actinomyces.43781
Porphyromonas.129
Actinomyces.27
Rothia.11690
Saccharibacteria_genera_incertae_sedis.42
Porphyromonas.82
Saccharibacteria_genera_incertae_sedis.15
Cellulosilyticum.18
Granulicatella.75
Streptococcus.73896
Neisseria.2014
Actinomyces.16789
Haemophilus.50
Parvimonas.29404
Rothia.11
Saccharibacteria_genera_incertae_sedis.14
Acinetobacter.202
Streptococcus.14938
Solobacterium.30
Tessaracoccus.147
Leptotrichia.119
Accumulated abundance rank
1
50
Rank Stability Index (in %)
99.8
95.4
95.0
84.7
89.1
87.6
79.9
89.5
83.9
46.7
82.1
83.9
48.6
73.3
43.6
23.5
66.0
65.4
75.7
50.8
76.8
51.3
37.2
62.9
45.0
69.9
34.4
56.3
27.1
65.0
40.8
48.8
62.2
73.1
41.9
65.5
46.8
39.2
67.1
26.5
47.0
38.1
34.8
45.7
47.8
19.7
33.4
38.5
27.5
29.8
Rank Variability
Differences Variability
RV (50 most abundant)
DV (50 most abundant)
Time samples
01 05 06 07 08 09 10 11 12 13 14 15 16 21 22 28

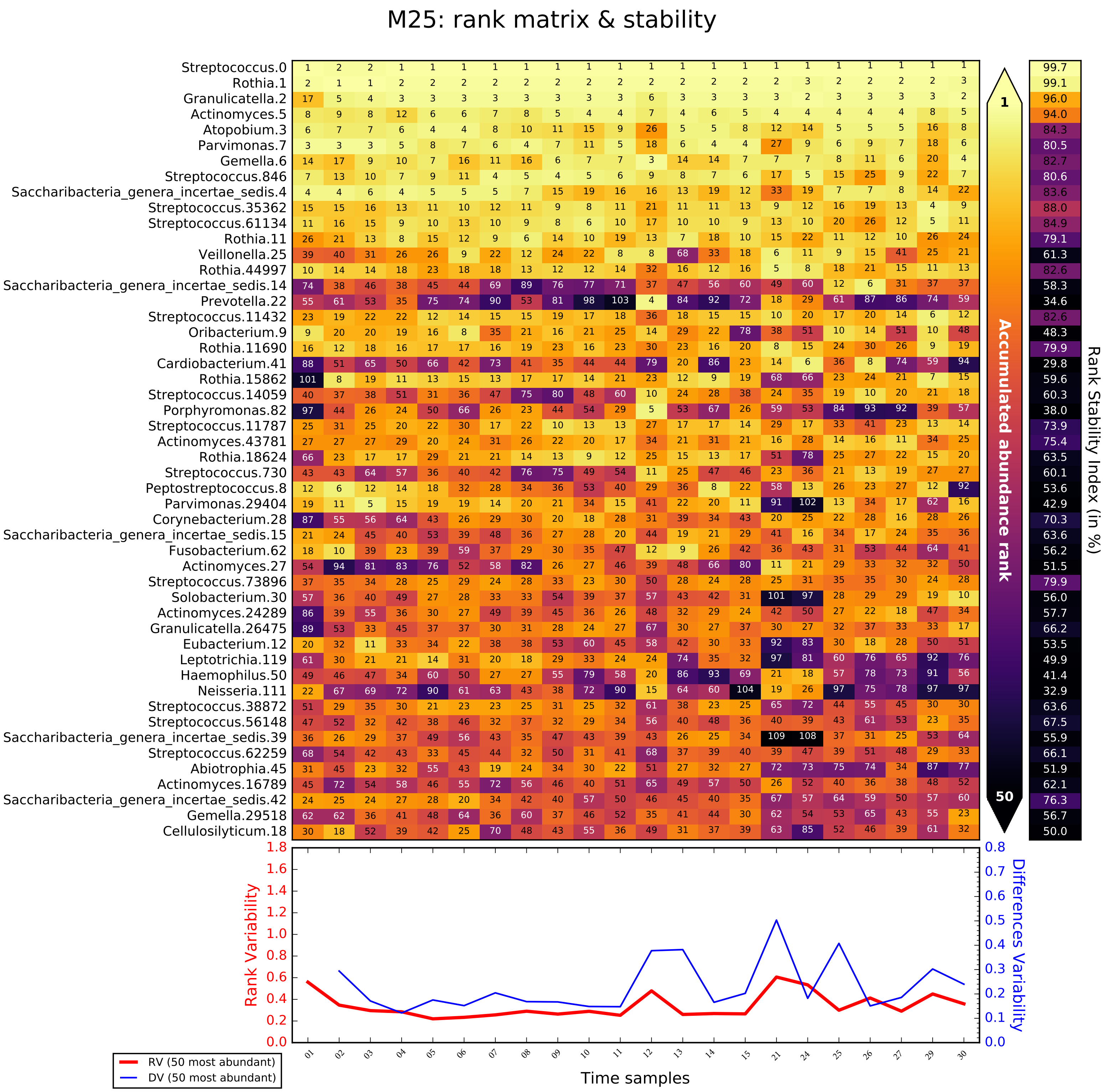

M25: rank matrix & stability
Streptococcus.0
Rothia.1
Granulicatella.2
Actinomyces.5
Atopobium.3
Parvimonas.7
Gemella.6
Streptococcus.846
Saccharibacteria_genera_incertae_sedis.4
Streptococcus.35362
Streptococcus.61134
Rothia.11
Veillonella.25
Rothia.44997
Saccharibacteria_genera_incertae_sedis.14
Prevotella.22
Streptococcus.11432
Oribacterium.9
Rothia.11690
Cardiobacterium.41
Rothia.15862
Streptococcus.14059
Porphyromonas.82
Streptococcus.11787
Actinomyces.43781
Rothia.18624
Streptococcus.730
Peptostreptococcus.8
Parvimonas.29404
Corynebacterium.28
Saccharibacteria_genera_incertae_sedis.15
Fusobacterium.62
Actinomyces.27
Streptococcus.73896
Solobacterium.30
Actinomyces.24289
Granulicatella.26475
Eubacterium.12
Leptotrichia.119
Haemophilus.50
Neisseria.111
Streptococcus.38872
Streptococcus.56148
Saccharibacteria_genera_incertae_sedis.39
Streptococcus.62259
Abiotrophia.45
Actinomyces.16789
Saccharibacteria_genera_incertae_sedis.42
Gemella.29518
Cellulosilyticum.18
1
Accumulated abundance rank
50
Rank Stability Index (in %)
99.7
99.1
96.0
94.0
84.3
80.5
82.7
80.6
83.6
88.0
84.9
79.1
61.3
82.6
58.3
34.6
82.6
48.3
79.9
29.8
59.6
60.3
38.0
73.9
75.4
63.5
60.1
53.6
42.9
70.3
63.6
56.2
51.5
79.9
56.0
57.7
66.2
53.5
49.9
41.4
32.9
63.6
67.5
55.9
66.1
51.9
62.1
76.3
56.7
50.0
Rank Variability
Differences Variability
01 02 03 04 05 06 07 08 09 10 11 12 13 14 15 21 24 25 26 27 29 30
Time samples
RV (50 most abundant)
DV (50 most abundant)

Supplementary Figure S6:

Boxplots showing values of AGE, AOPP, FRAS, TAC and TBARS for each volunteer. The box represents middle 50% of scores which fall within the inter-quartile range, while median marks the mid-point.

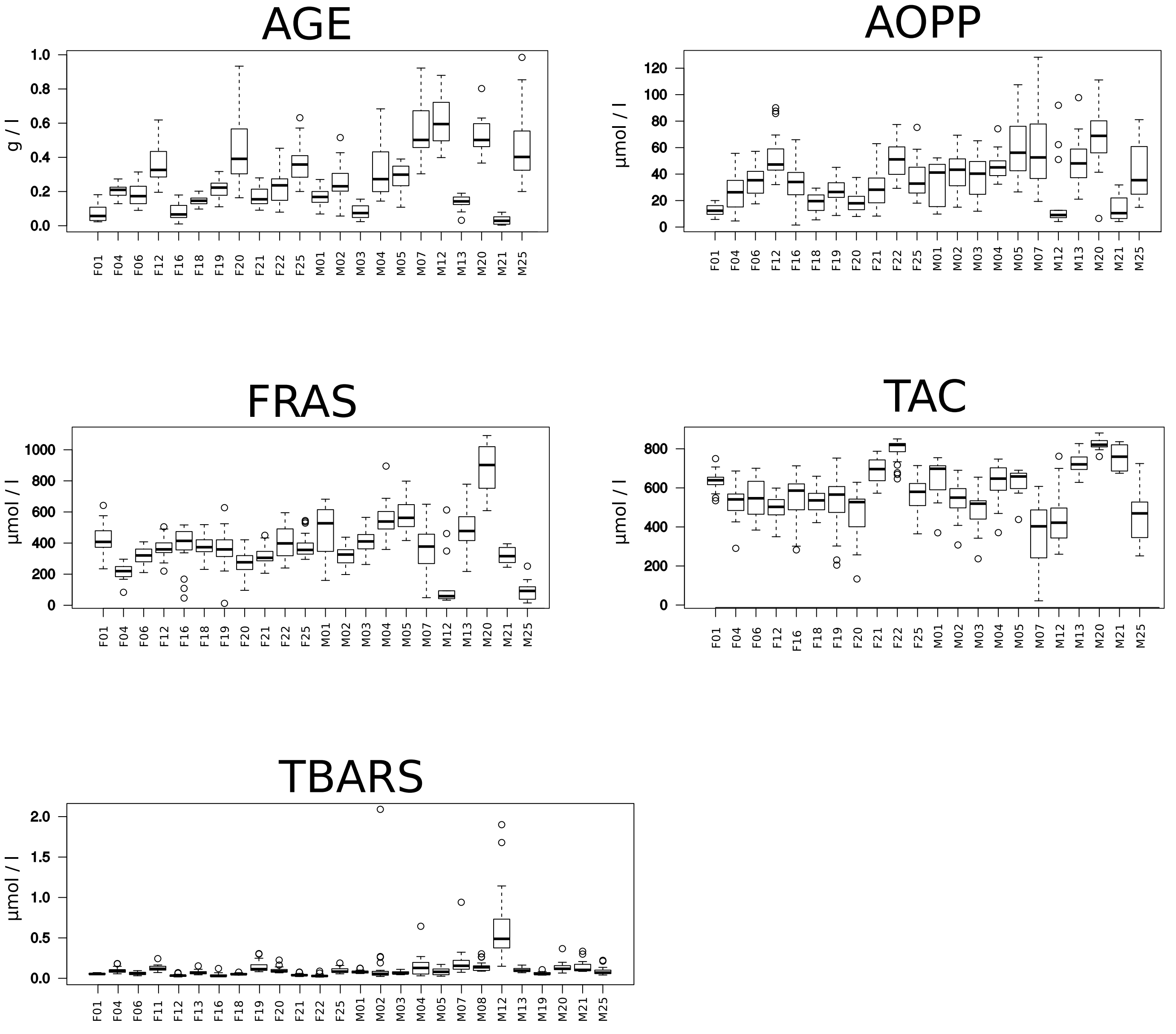

Supplementary Figure S7:

Correlation plots summarizing Pearson's and Spearman's correlations on intra-individual level. Both temporalily direct and lagged correlations were calculated. The color bar on the right of each panel indicates in how many individuals a negative (red) or a positive (blue) correlation was detected.

Pearson's correlations

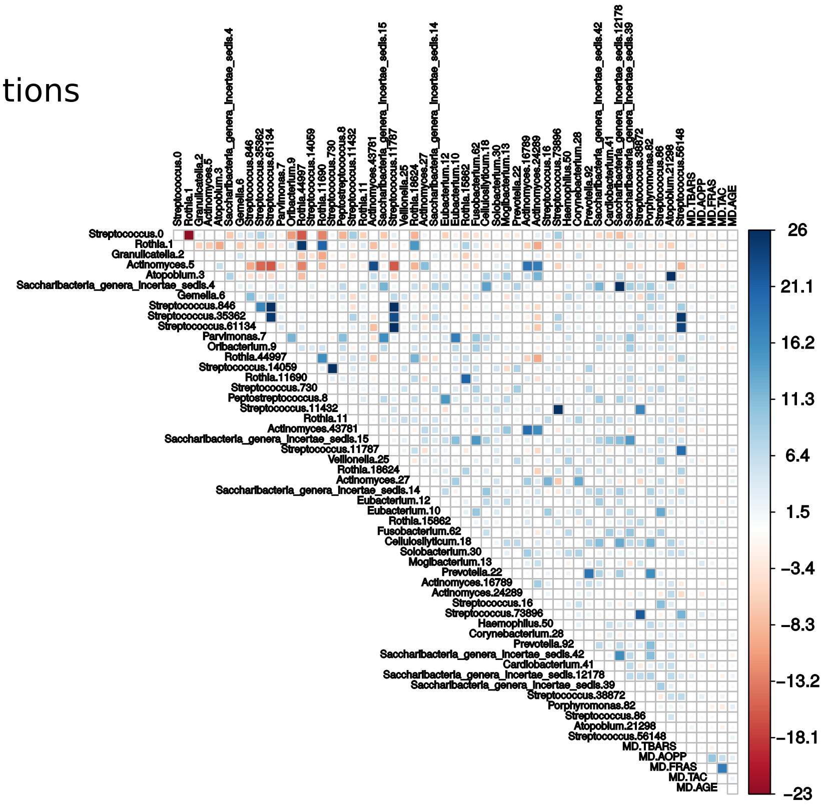

Spearman's correlations

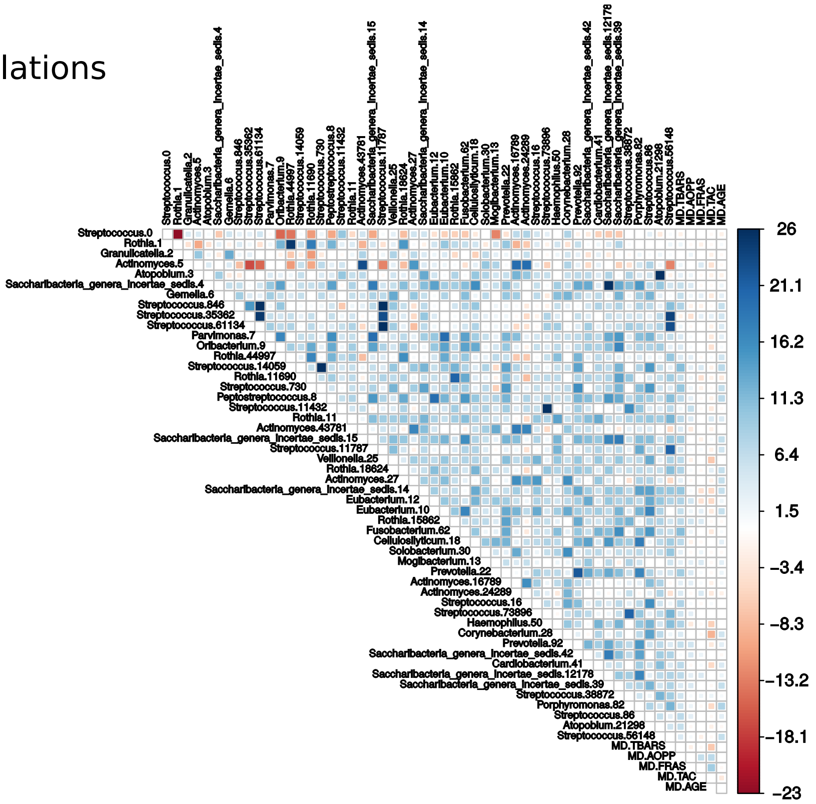

Supplementary Figure S8:

Community interaction plots between the 15 most prevalent OTUs per sample based on the Lotka-Volterra model. This dynamic model of ecological interactions in temporal samples series counts with the situation in which the OTU #1 interacts with OTU #2, but the OTU #2 does not have to interact necessarily with OTU #1. In each case, the blue color represents negative interaction between two OTUs, whereas orange represents positive interactions. The diagonal in the matrix represents the auto-interaction of each OTU. The interaction values were normalized at a logarithmic scale for representative purposes.

F01

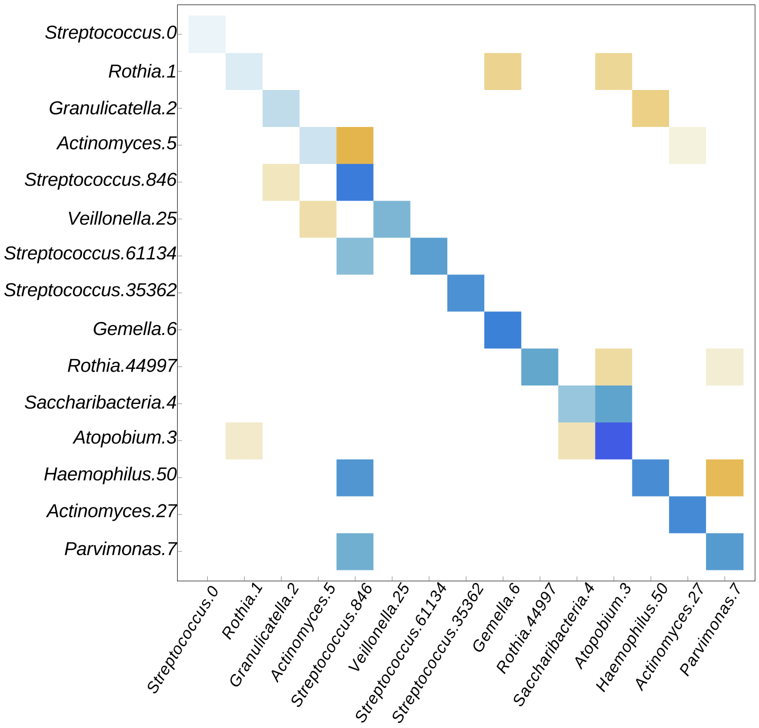

F04

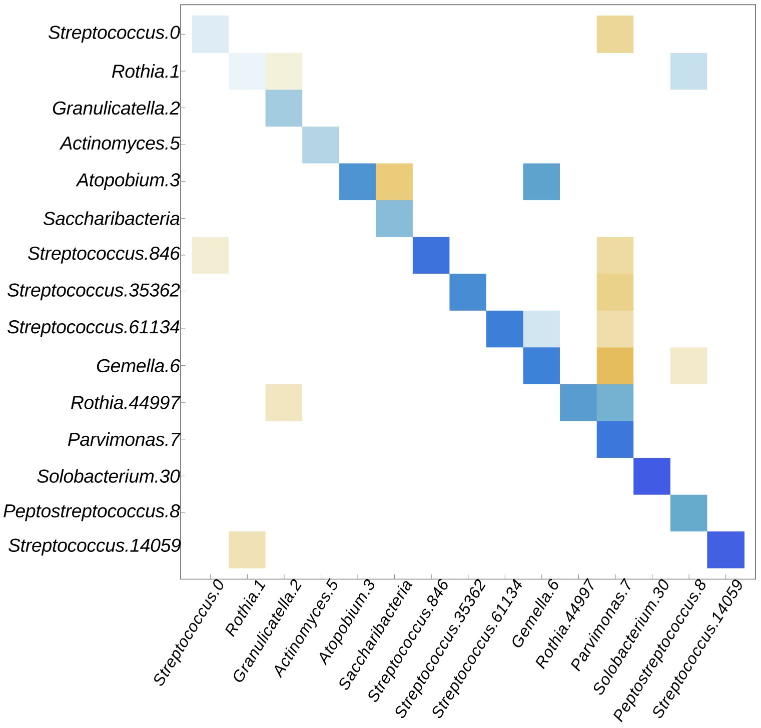

F06

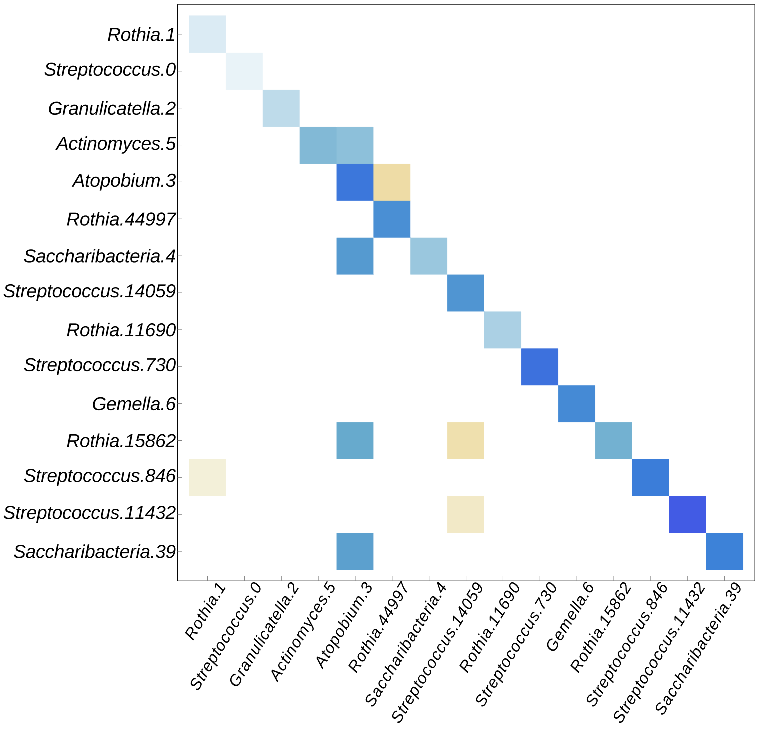

F11

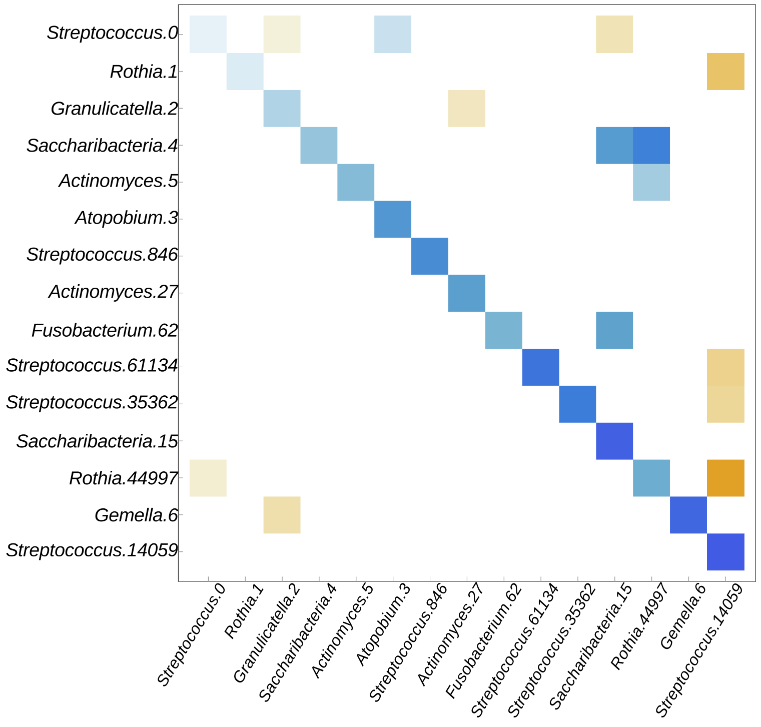

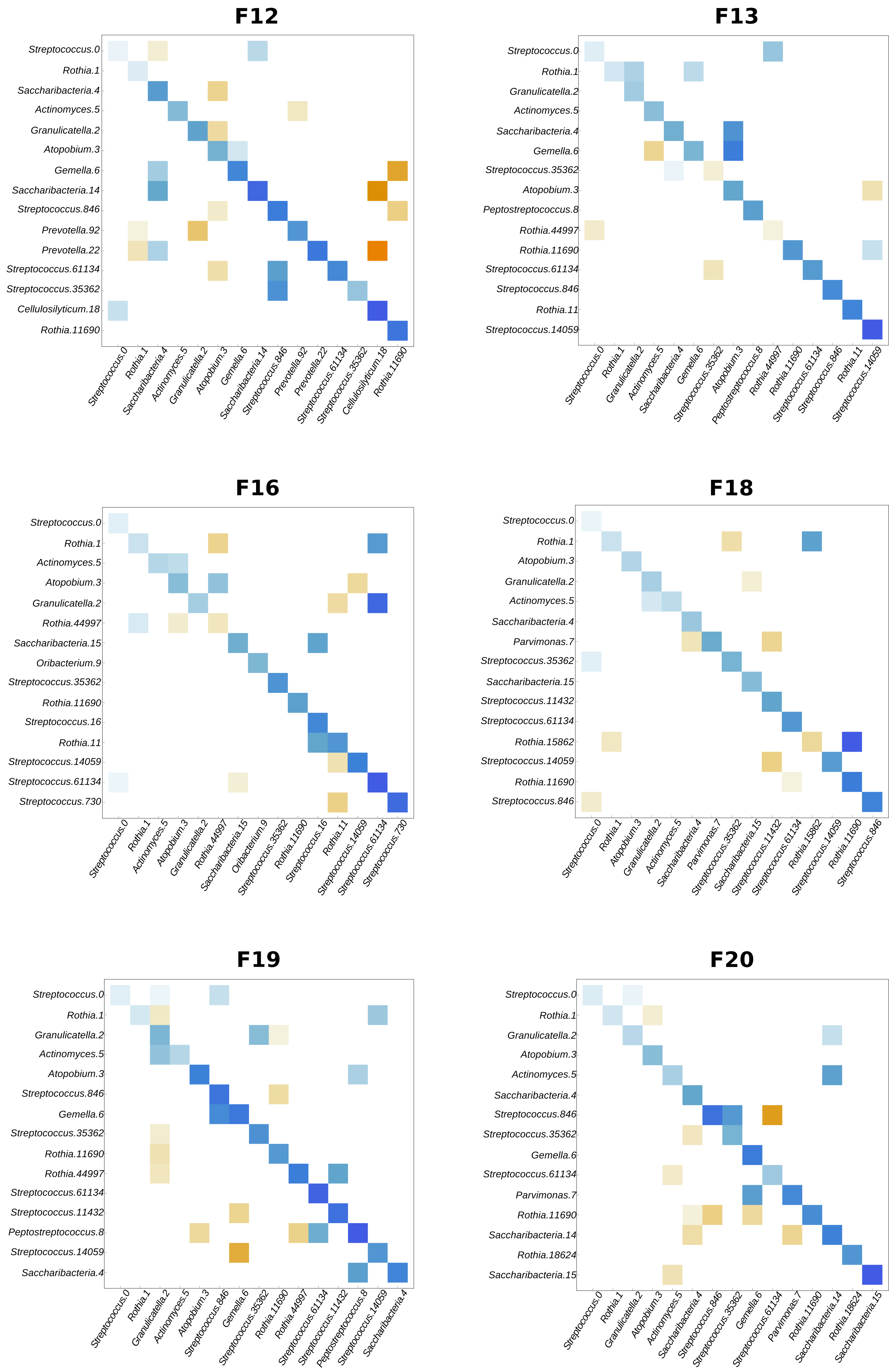
F12
Streptococcus.0
Rothia.1
Saccharibacteria.4
Actinomyces.5
Granulicatella.2
Atopobium.3
Gemella.6
Saccharibacteria.14
Streptococcus.846
Prevotella.92
Prevotella.22
Streptococcus.61134
Streptococcus.35362
Cellulosilyticum.18
Rothia.11690
F13
Streptococcus.0
Rothia.1
Granulicatella.2
Actinomyces.5
Saccharibacteria.4
Gemella.6
Streptococcus.35362
Atopobium.3
Peptostreptococcus.8
Rothia.44997
Rothia.11690
Streptococcus.61134
Streptococcus.846
Rothia.11
Streptococcus.14059
F16
Streptococcus.0
Rothia.1
Actinomyces.5
Atopobium.3
Granulicatella.2
Rothia.44997
Saccharibacteria.15
Oribacterium.9
Streptococcus.35362
Rothia.11690
Streptococcus.16
Rothia.11
Streptococcus.14059
Streptococcus.61134
Streptococcus.730
F18
Streptococcus.0
Rothia.1
Atopobium.3
Granulicatella.2
Actinomyces.5
Saccharibacteria.4
Parvimonas.7
Streptococcus.35362
Saccharibacteria.15
Streptococcus.11432
Streptococcus.61134
Rothia.15862
Streptococcus.14059
Rothia.11690
Streptococcus.846
F19
Streptococcus.0
Rothia.1
Granulicatella.2
Actinomyces.5
Atopobium.3
Streptococcus.846
Gemella.6
Streptococcus.35362
Rothia.11690
Rothia.44997
Streptococcus.61134
Streptococcus.11432
Peptostreptococcus.8
Streptococcus.14059
Saccharibacteria.4
F20
Streptococcus.0
Rothia.1
Granulicatella.2
Atopobium.3
Actinomyces.5
Saccharibacteria.4
Streptococcus.846
Streptococcus.35362
Gemella.6
Streptococcus.61134
Parvimonas.7
Rothia.11690
Saccharibacteria.14
Rothia.18624
Saccharibacteria.15

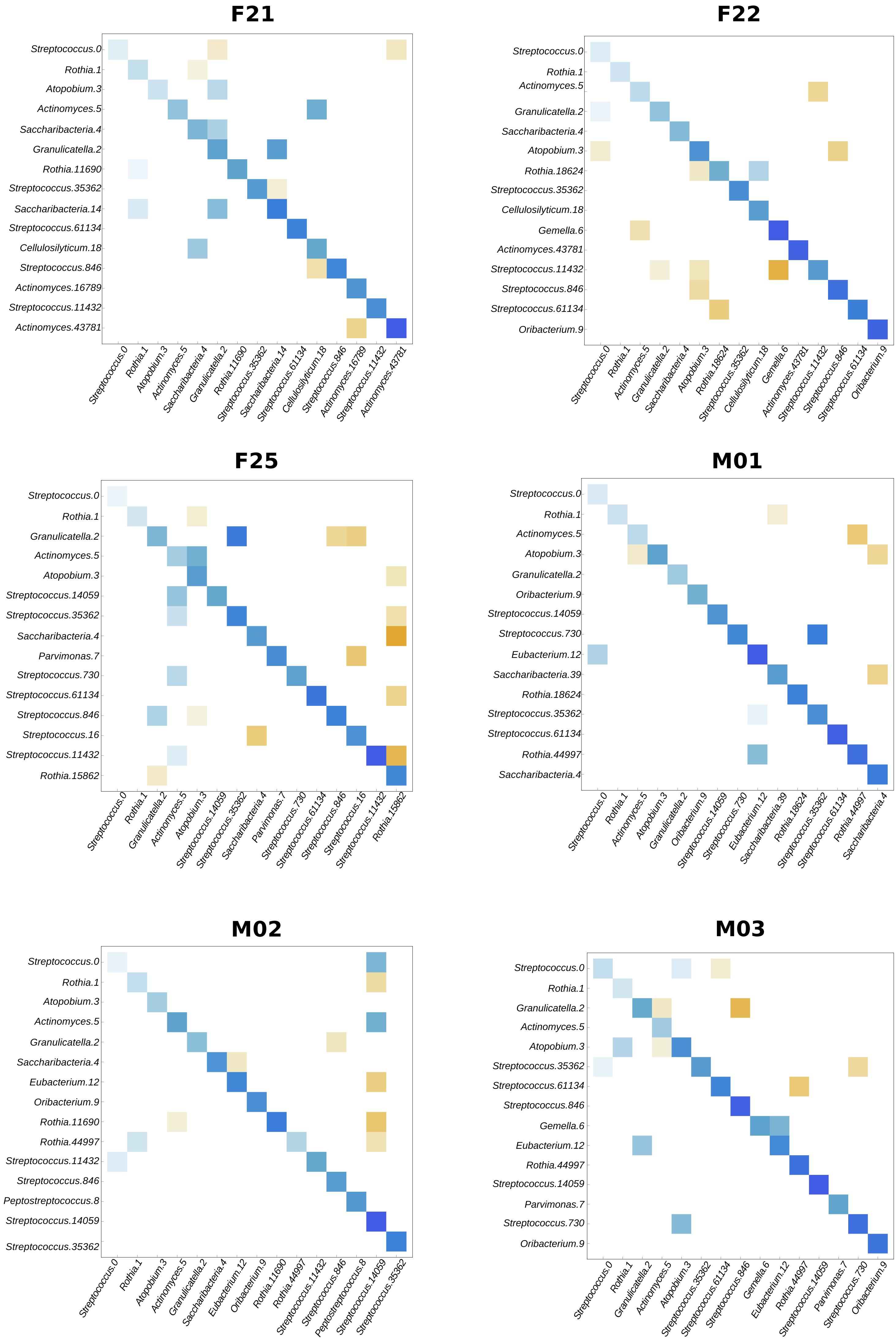
F21
Streptococcus.0
Rothia.1
Atopobium.3
Actinomyces.5
Saccharibacteria.4
Granulicatella.2
Rothia.11690
Streptococcus.35362
Saccharibacteria.14
Streptococcus.61134
Cellulosilyticum.18
Streptococcus.846
Actinomyces.16789
Streptococcus.11432
Actinomyces.43781
F22
Streptococcus.0
Rothia.1
Actinomyces.5
Granulicatella.2
Saccharibacteria.4
Atopobium.3
Rothia.18624
Streptococcus.35362
Cellulosilyticum.18
Gemella.6
Actinomyces.43781
Streptococcus.11432
Streptococcus.846
Streptococcus.61134
Oribacterium.9
F25
Streptococcus.0
Rothia.1
Granulicatella.2
Actinomyces.5
Atopobium.3
Streptococcus.14059
Streptococcus.35362
Saccharibacteria.4
Parvimonas.7
Streptococcus.730
Streptococcus.61134
Streptococcus.846
Streptococcus.16
Streptococcus.11432
Rothia.15862
M01
Streptococcus.0
Rothia.1
Actinomyces.5
Atopobium.3
Granulicatella.2
Oribacterium.9
Streptococcus.14059
Streptococcus.730
Eubacterium.12
Saccharibacteria.39
Rothia.18624
Streptococcus.35362
Streptococcus.61134
Rothia.44997
Saccharibacteria.4
M02
Streptococcus.0
Rothia.1
Atopobium.3
Actinomyces.5
Granulicatella.2
Saccharibacteria.4
Eubacterium.12
Oribacterium.9
Rothia.11690
Rothia.44997
Streptococcus.11432
Streptococcus.846
Peptostreptococcus.8
Streptococcus.14059
Streptococcus.35362
M03
Streptococcus.0
Rothia.1
Granulicatella.2
Actinomyces.5
Atopobium.3
Streptococcus.35362
Streptococcus.61134
Streptococcus.846
Gemella.6
Eubacterium.12
Rothia.44997
Streptococcus.14059
Parvimonas.7
Streptococcus.730
Oribacterium.9

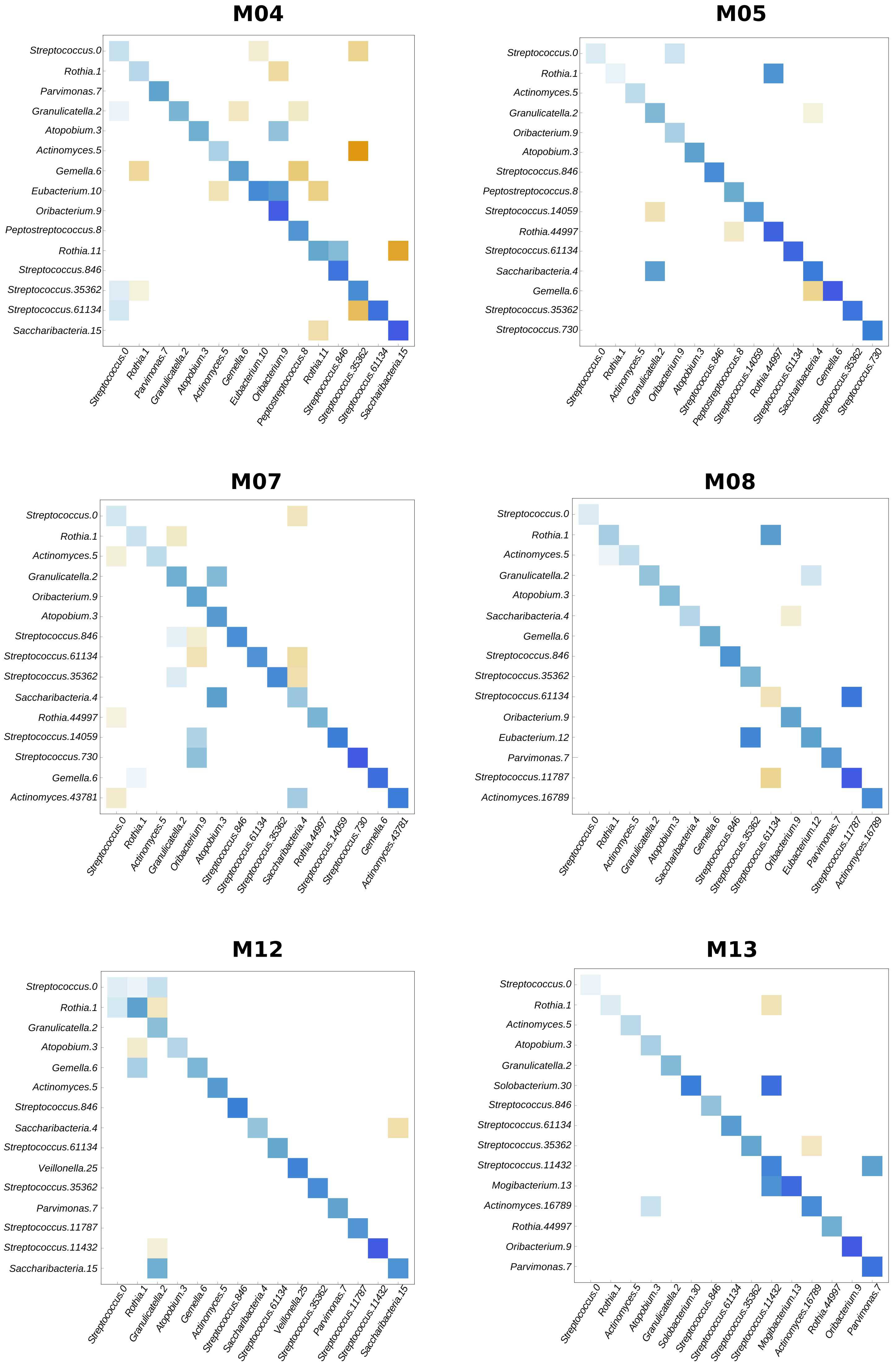
M04
Streptococcus.0
Rothia.1
Parvimonas.7
Granulicatella.2
Atopobium.3
Actinomyces.5
Gemella.6
Eubacterium.10
Oribacterium.9
Peptostreptococcus.8
Rothia.11
Streptococcus.846
Streptococcus.35362
Streptococcus.61134
Saccharibacteria.15
M05
Streptococcus.0
Rothia.1
Actinomyces.5
Granulicatella.2
Oribacterium.9
Atopobium.3
Streptococcus.846
Peptostreptococcus.8
Streptococcus.14059
Rothia.44997
Streptococcus.61134
Saccharibacteria.4
Gemella.6
Streptococcus.35362
Streptococcus.730
M07
Streptococcus.0
Rothia.1
Actinomyces.5
Granulicatella.2
Oribacterium.9
Atopobium.3
Streptococcus.846
Streptococcus.61134
Streptococcus.35362
Saccharibacteria.4
Rothia.44997
Streptococcus.14059
Streptococcus.730
Gemella.6
Actinomyces.43781
M08
Streptococcus.0
Rothia.1
Actinomyces.5
Granulicatella.2
Atopobium.3
Saccharibacteria.4
Gemella.6
Streptococcus.846
Streptococcus.35362
Streptococcus.61134
Oribacterium.9
Eubacterium.12
Parvimonas.7
Streptococcus.11787
Actinomyces.16789
M12
Streptococcus.0
Rothia.1
Granulicatella.2
Atopobium.3
Gemella.6
Actinomyces.5
Streptococcus.846
Saccharibacteria.4
Streptococcus.61134
Veillonella.25
Streptococcus.35362
Parvimonas.7
Streptococcus.11787
Streptococcus.11432
Saccharibacteria.15
M13
Streptococcus.0
Rothia.1
Actinomyces.5
Atopobium.3
Granulicatella.2
Solobacterium.30
Streptococcus.846
Streptococcus.61134
Streptococcus.35362
Streptococcus.11432
Mogibacterium.13
Actinomyces.16789
Rothia.44997
Oribacterium.9
Parvimonas.7

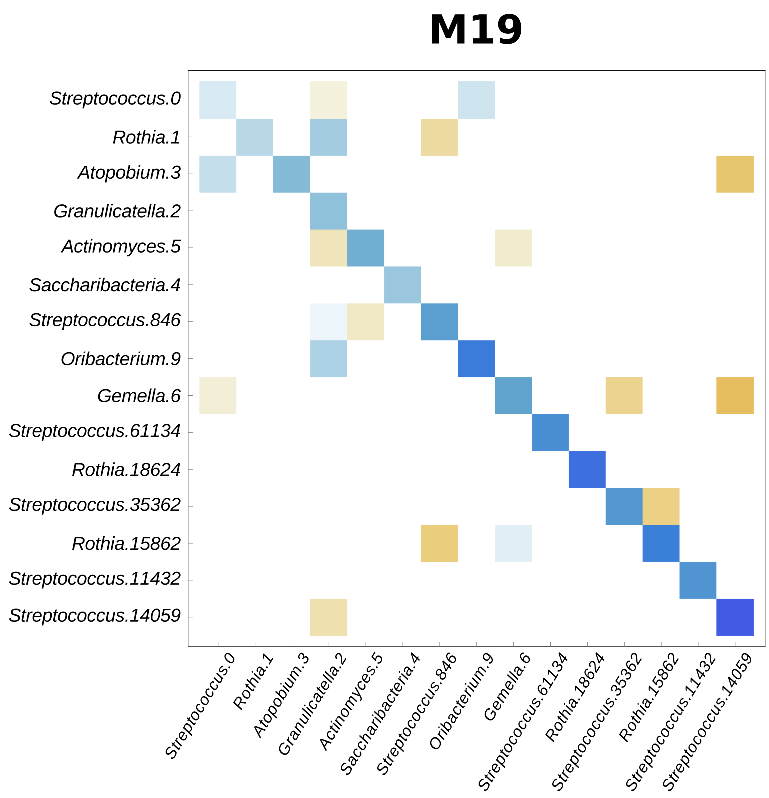
M19
Streptococcus.0
Rothia.1
Atopobium.3
Granulicatella.2
Actinomyces.5
Saccharibacteria.4
Streptococcus.846
Oribacterium.9
Gemella.6
Streptococcus.61134
Rothia.18624
Streptococcus.35362
Rothia.15862
Streptococcus.11432
Streptococcus.14059
Streptococcus.0
Rothia.1
Atopobium.3
Granulicatella.2
Actinomyces.5
Saccharibacteria.4
Streptococcus.846
Oribacterium.9
Gemella.6
Streptococcus.61134
Rothia.18624
Streptococcus.35362
Rothia.15862
Streptococcus.11432
Streptococcus.14059

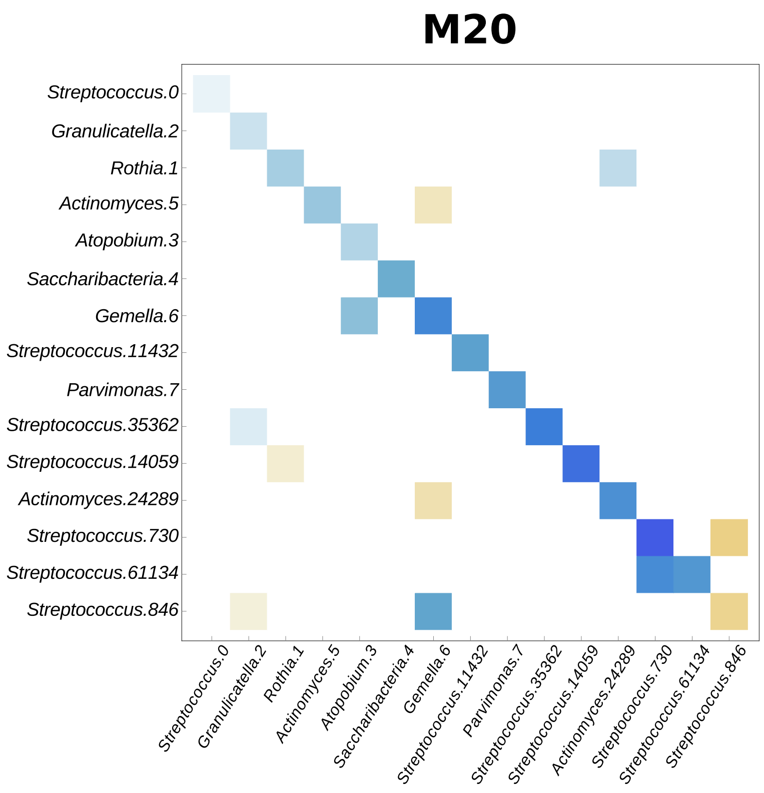
M20
Streptococcus.0
Granulicatella.2
Rothia.1
Actinomyces.5
Atopobium.3
Saccharibacteria.4
Gemella.6
Streptococcus.11432
Parvimonas.7
Streptococcus.35362
Streptococcus.14059
Actinomyces.24289
Streptococcus.730
Streptococcus.61134
Streptococcus.846
Streptococcus.0
Granulicatella.2
Rothia.1
Actinomyces.5
Atopobium.3
Saccharibacteria.4
Gemella.6
Streptococcus.11432
Parvimonas.7
Streptococcus.35362
Streptococcus.14059
Actinomyces.24289
Streptococcus.730
Streptococcus.61134
Streptococcus.846

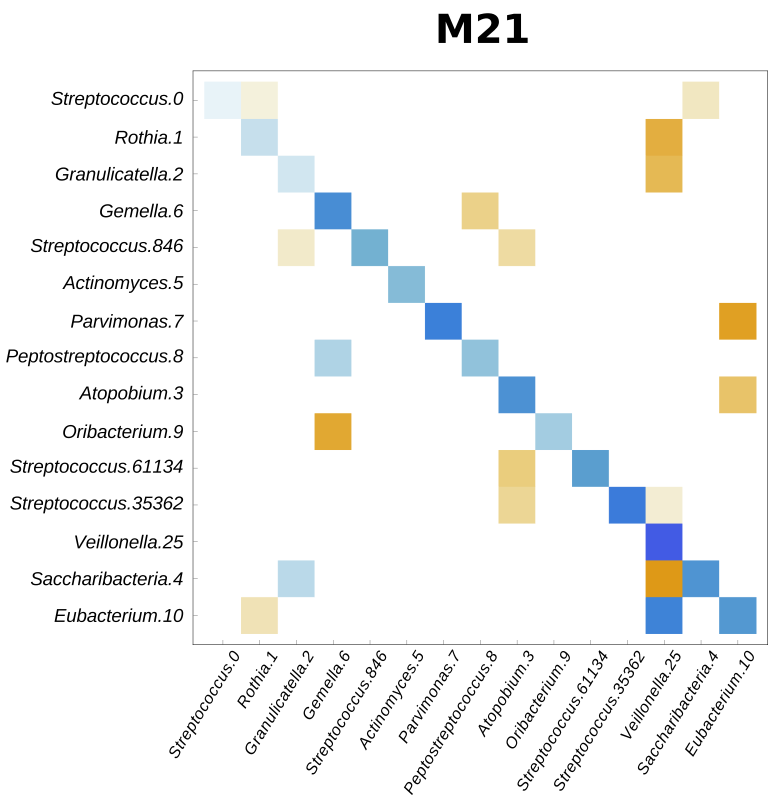
M21
Streptococcus.0
Rothia.1
Granulicatella.2
Gemella.6
Streptococcus.846
Actinomyces.5
Parvimonas.7
Peptostreptococcus.8
Atopobium.3
Oribacterium.9
Streptococcus.61134
Streptococcus.35362
Veillonella.25
Saccharibacteria.4
Eubacterium.10
Streptococcus.0
Rothia.1
Granulicatella.2
Gemella.6
Streptococcus.846
Actinomyces.5
Parvimonas.7
Peptostreptococcus.8
Atopobium.3
Oribacterium.9
Streptococcus.61134
Streptococcus.35362
Veillonella.25
Saccharibacteria.4
Eubacterium.10

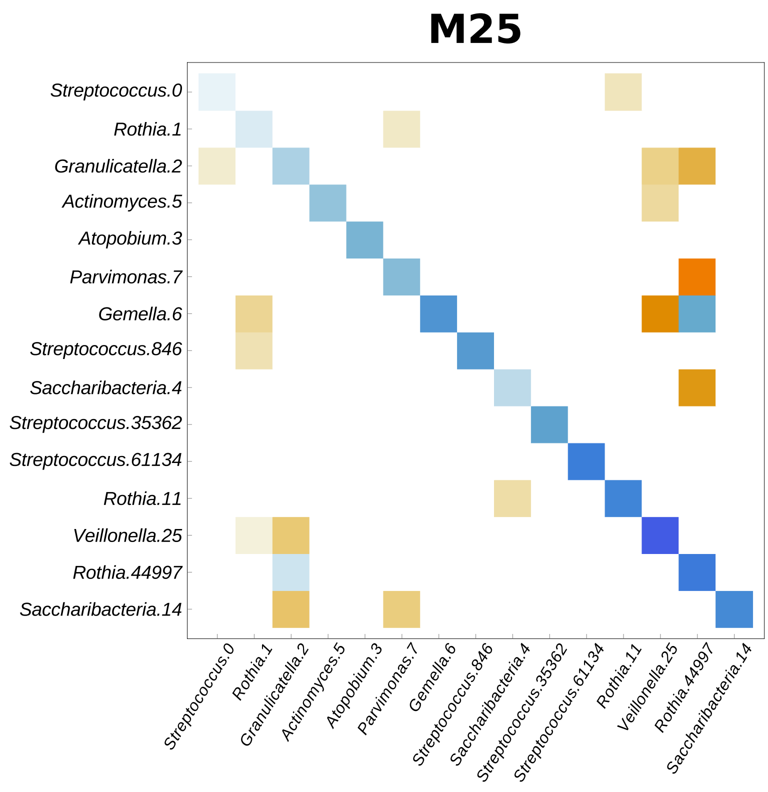
M25
Streptococcus.0
Rothia.1
Granulicatella.2
Actinomyces.5
Atopobium.3
Parvimonas.7
Gemella.6
Streptococcus.846
Saccharibacteria.4
Streptococcus.35362
Streptococcus.61134
Rothia.11
Veillonella.25
Rothia.44997
Saccharibacteria.14
Streptococcus.0
Rothia.1
Granulicatella.2
Actinomyces.5
Atopobium.3
Parvimonas.7
Gemella.6
Streptococcus.846
Saccharibacteria.4
Streptococcus.35362
Streptococcus.61134
Rothia.11
Veillonella.25
Rothia.44997
Saccharibacteria.14
